# ‘One-pot’ Synthesis of Dihydrobenzo[4,5][1,3]oxazino[2,3-*a*]isoquinolines via a Silver(I)-Catalyzed Cascade Approach

**DOI:** 10.3390/molecules18010814

**Published:** 2013-01-11

**Authors:** Baifeng Jiang, Yu Zhou, Qingya Kong, Hualiang Jiang, Hong Liu, Jian Li

**Affiliations:** 1 Shanghai Key Laboratory of New Drug Design, School of Pharmacy, East China University of Science and Technology, 130 Mei Long Road, Shanghai 200237, China; 2 State Key Laboratory of Drug Research, Shanghai Institute of Materia Medica, Chinese Academy of Sciences, 555 Zu Chong Zhi Road, Shanghai 201203, China

**Keywords:** silver, one-pot, cascade transformation, isoquinolines, heterocyclic molecule

## Abstract

An efficient approach for the synthesis of biologically interesting fused tetracyclic isoquinolines in high yields and with a broad substrate scope has been developed. The strategy features an AgNO_3_ catalyzed ‘one-pot’ cascade process involving formation of two new C–N bonds and one new C–O bond.

## 1. Introduction

Fused isoquinolines are widely distributed in alkaloids and biologically important synthetic substances. They exhibit a broad spectrum of biological properties, such as antitumor activity [1,2], TC-PTP and PTP1B inhibitory activities [3], and HIV inhibitory potencies [4,5]. Therefore, the development of new synthetic strategies for the efficient preparation of fused isoquinolines is in considerable demand. Compared to the stepwise approach, cascade reactions represent an attractive strategy in synthesis because multiple bond-forming and -cleaving events can be combined into a single reaction operation. Recently, transition-metal-catalyzed cascade processes have received significant attention [6,7,8,9,10,11,12,13,14,15,16,17,18,19]. Among these transition metals, silver salts have long been believed to have low catalytic efficiency, and most commonly served as either co-catalysts or weak Lewis acids [20]. However, in recent years they have been extensively employed to activate alkyne, alkene, and allene functionalities under mild conditions and at low catalyst loadings [21,22,23,24,25,26,27,28,29,30,31,32,33,34].

**Scheme 1:**
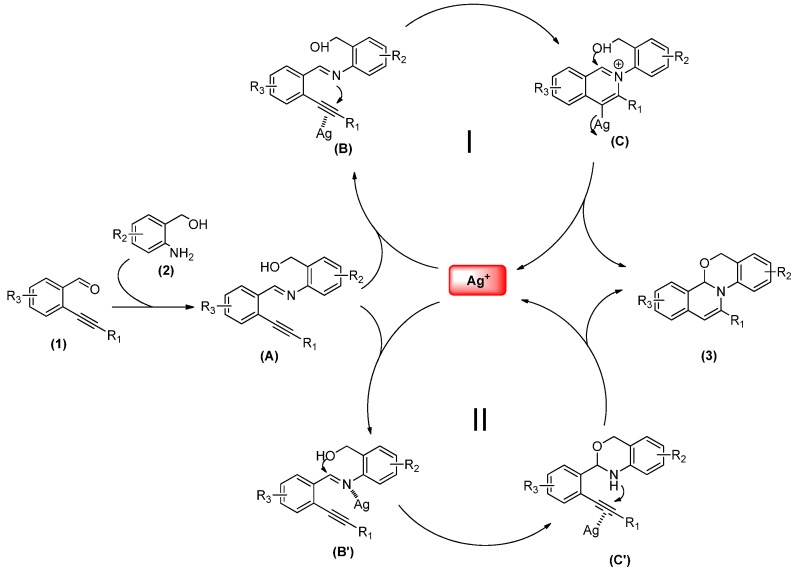
A plausible mechanism.

In our ongoing efforts for the development of the efficient synthetic methods for the construction of potential bioactive fused polycyclic compounds through exploration of new catalytic cascade strategies [35,36,37,38,39,40,41], we envisioned that the direct assembly of nitrogen-containing tetracyclic isoquinoline structures **3** could be realized in a ‘one-pot’ operation from readily available and functional 2-substituted-ethynyl benzaldehydes **1** with 2-aminoarylmethanols **2** (Scheme 1). In this design, the condensation of the starting materials **1** and **2** would be primed for the formation of the Ag-complex (**B** or **B′**) in the presence of Ag salts as catalyst. The resulting species would be subject to a cascade nucleophilic attack to give the target scaffold **3**. Successful execution of the proposal would lead to highly functionalized isoquinolines, which are particularly attractive for further elaboration in diversity oriented synthesis. Recently, some progress has been made via tandem nucleophilic addition and cyclization to give fused tetracyclic isoquinolines by using *o*-alkynylbenzaldehyde as the starting material in the presence of various Lewis acid catalysts, such as AuCl [17], In(OTf)_3_ [18], Yb(OTf)_3_ [13], CuI [16], AgNO_3_ [28,29], and Ph_3_PAuMe/chiral Brønsted acids [15]. However, to the best of our knowledge, there is no report involving the synthesis of dihydrobenzo[4,5][1,3]oxazino[2,3-*a*]isoquinolines **3** via a AgNO_3_-catalyzed one-pot domino process. Herein, we wish to disclose our recent results in this area.

## 2. Results and Discussion

To fulfill the hypothesis, we carried out the experiments using 2-(phenylethynyl)benzaldehyde (**1a**) and (2-aminophenyl)methanol (**2a**) as model substrates (as shown in Table 1). In our previous studies, we found that some gold-complex and/or silver salts were highly efficient catalysts for cascade reactions involving the activation of alkynyl groups [35,36,37,38,39,40,41]. Therefore, several gold catalysts, including AuCl(PPh_3_), AuCl, (acetonitrile)[(2-biphenyl)di-*tert*-butylphosphine]gold(I) hexafluoro-antimonate (Au catalyst I), and 2-dicyclohexylphosphino-2′,4′,6′-triisopropyl-biphenyl gold(I) bis(trifluoromethanesulfonyl)imide (Au catalyst II) were firstly investigated in a sealed tube using dry toluene as the solvent at 100 °C for 3 h. Disappointingly, the desirable cascade products were obtained in only 28%–75% yields (Table 1, entries 1–4). Different silver salts, such as AgOOCCF_3_, AgNO_3_, AgBF_4_ and AgOTf, were also subsequently screened, and AgNO_3_ was proved to be the most effective one for this transformation (Table 1, entries 5–8), and the product **3Aa** could be obtained in 93% yield (Table 1, entry 6). It is apparent that the solvent has a significant influence on the yield of this reaction (Table 1, entries 9–14). Toluene was found to be the optimal solvent for this transformation (Table 1, entries 6, 9–14), although when DMSO, CH_3_CN and 1,4-dioxane were used instead of toluene, the desired product **3Aa** can also obtained with good yields (Table 1, entries 12–14). The yield of the product has no significant difference when we decrease the reaction temperature to 80 °C in toluene (Table 1, entry 15). However, when DMSO, CH_3_CN and 1,4-dioxane were used, decreasing temperature appears to have a negative impact on the yield of product (Table 1, entries 16–18). We further decreased reaction temperature to 50 °C and room temperature, but these changes adversely affected the product yield, and product **3Aa** was obtained in only 65% and 30% yield, respectively (Table 1, entries 19–20). 

Furthermore, the amount of starting material **2a** was also screened, and the results demonstrated that the same excellent yield for desired product **3Aa** were observed in toluene at 80 °C for 3 h when 1.2 equiv. of **2a** was used (Table 1, entry 21).

To explore the scope and limitation of this cascade reaction, we surveyed the diversity of the starting materials by the structural variations of both 2-substituted-ethynyl benzaldehydes (**1A**–**H**) and 2-aminoarylmethanols (**2a**–**j**). As shown in Table 2, notably, the corresponding fused tetracyclic isoquinolines products **3Aa**–**Bj** were efficiently produced in moderate to excellent yields (45%–94%). The nature of the 2-aminoarylmethanol and the substituents attached to the triple bond of benzaldehydes has a major impact on the yield of the transformation. When 2-phenylethynyl-benzaldehyde (**1A**) was treated with different substituted 2-aminoarylmethanols **2a**–**e**, excellent yields were achieved (Table 2, entries 1–5). Moreover, when introducing substituent groups such as fluoro, chloro, or methyl groups onto the phenyl ring in the 2-phenylethynylbenzaldehydes, most of the desired products were obtained with high yields (Table 2, entries 6–22). Nevertheless, some substituted 2-aminoarylmethanols with bromo or methyl groups (Table 2, entries 8–10, 17–18 and 22), especially 6-methyl-2-aminophenylmethanol (Table 2, entry 10), result in a decrease in the yield of the target products, presumably due to the influence of electronic and/or steric effects. Subsequently, we prepared substrates **1F** and **1G** by introducing methyl and fluoro groups in the 4-position and 5-postion of 2-phenylethynylbenzaldehyde (**1A**), respectively. Results of further investigations demonstrated that all tested substrates were tolerated in this cascade transformation, giving good to excellent yields (Table 2, entries 23–30). However, a relatively lower 55% yield of product **3Gh** was obtained in the reaction of 2-phenylethynyl-4-methylbenzaldehyde (**1G**) with 3-aminonaphthalenylmethanol (**2h**). We speculate that the electronic effect of naphthalene ring was responsible for the decrease in the yield. Furthermore, an alkyl (*n*-hexyl) group at the R^1^ position was also tolerated, and excellent yields were obtained (Table 2, entries 31–32). Finally, a *N*-containing heterocyclic substrate (2-amino-pyridinylmethanol, **2j**) was investigated in this cascade transformation, but only moderate yields were obtained (Table 2, entries 33–34). In view of these findings, this cascade strategy could serve as a general approach for the preparation of fused tetracyclic isoquinoline complex molecular architectures.

**Figure 1:**
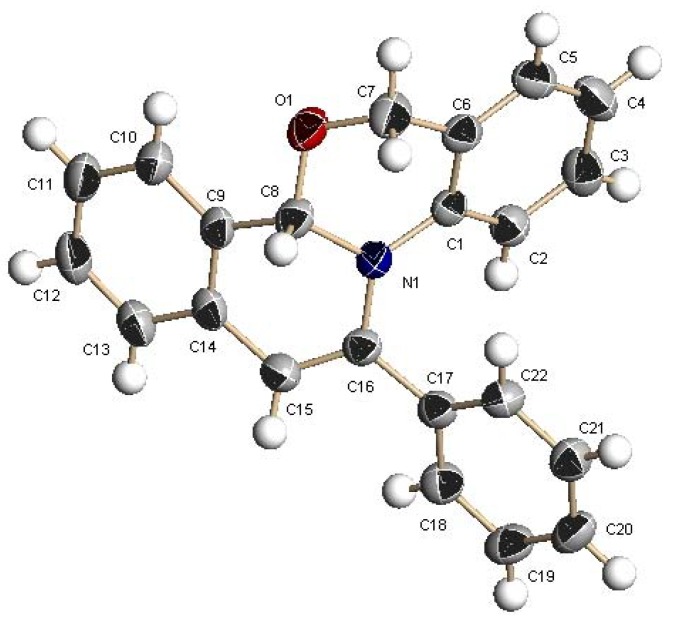
X-ray crystallographic structure of **3Aa**.

Scheme 1 depicts a plausible mechanism for this cascade transformation. The condensation of the starting materials **1** and **2** generates the key imine intermediate **A**, which subsequently can be converted into the final product **3** via two possible catalytic pathways (I and II). In pathway I [17,42], the imine intermediate **A** is activated by AgNO_3_ to form the π-Ag complex **B**, and futher generates the *N*-aryl imine cation **C** through an intramolecular nucleophilic addition, which is subject to a subsequent nucleophilic attack and proton transformation to afford the target scaffold **3**. In the conceivable alternative pathway II [17,28], the aminal intermediate **C′** is probably formed by an intramolecular nucleophilic addition of iminoalkyne Ag-complex **B′**. Then, the Ag-mediated intramolecular hydroamination reaction of **C′** and subsequent protonation results in the formation of the target product **3**. The target product **3Aa** was further characterized by X-ray crystallography (Figure 1, see Supporting Information for details).

## 3. Experimental 

### 3.1. General

2-Aminoarylmethanols **2a**–**j** and the two 2-substituted-ethynyl benzaldehydes **1A** and **2B** are commercially available starting materials. The five remaining 2-substituted-ethynyl benzaldehydes **1C**–**H** were prepared as indicated in the following methods. Commercially available reagents and solvents were used without further purification. Column chromatography was carried out on silica gel. ^1^H and ^13^C-NMR spectra were obtained on Varian Mercury-300, Varian Mercury-400 and Varian Mercury-500 spectrometers (TMS as IS). Chemical shifts were reported in parts per million (ppm, δ) downfield from tetramethylsilane. Proton coupling patterns are described as singlet (s), doublet (d), triplet (t), quartet (q), multipet (m) and broad (br). Low- and high-resolution mass spectra (LRMS and HRMS) were measured on a Finnigan MAT 95 spectrometer.

### 3.2. General Procedure for Synthesis of 2-Substituted-ethynyl Benzaldehyde Derivatives ***1C**–**H*** *(**1C*** as an Example*)*

To a solution of 2-bromobenzaldehyde (0.37 g), PdCl_2_(PPh_3_)_2_ (28 mg), and CuI (3.8 mg) in of Et_3_N (20 mL) was added phenylacetylene (0.2 g). The resulting mixture was heated under a nitrogen atmosphere at 60 °C for 4 h. After the reaction was completed, the reaction mixture was concentrated under reduced pressure, and the resulting residue was purified by flash column chromatography (PE/EA = 50/1, v/v, as an eluent) to give 2-((4-chlorophenyl)ethynyl)benzaldehyde (**1C**) in 90% yield. ^1^H-NMR (500 MHz, CDCl_3_) δ 10.61 (s, 1H), 7.94 (d, *J* = 7.8 Hz, 1H), 7.63 (d, *J* = 7.6 Hz, 1H), 7.58 (t, *J* = 7.5 Hz, 1H), 7.47 (m, 3H), 7.35 (d, *J* = 8.4 Hz, 2H). ^13^C-NMR (125 MHz, CDCl_3_) δ 191.47, 135.84, 135.19, 133.84, 133.25, 132.91, 128.93, 128.86, 127.46, 126.41, 120.83, 95.09, 85.91. LRMS (ESI) *m/z* 241 [M+H]^+^; HRMS (ESI) *m/z* calcd C_15_H_9_ClONa [M+Na]^+^ 263.0240, found 263.0243.

*2-(p-Tolylethynyl)benzaldehyde* (**1D**). ^1^H-NMR (400 MHz, CDCl_3_) δ 10.68 (s, 1H), 7.97 (d, *J* = 7.2 Hz, 1H), 7.66 (d, *J* = 7.2 Hz, 1H), 7.62 (m, 1H), 7.49 (m, 3H), 7.23 (d, *J* = 8.0 Hz, 2H), 2.42 (s, 3H). ^13^C-NMR (125 MHz, CDCl_3_) δ 191.87, 139.42, 135.77, 133.81, 133.17, 131.62, 129.34, 128.45, 127.21, 119.27, 96.69, 84.35, 21.63. LRMS (ESI) *m/z* 221 [M+H]^+^; HRMS (ESI) *m/z* calcd C_16_H_12_ONa [M+Na]^+^ 243.0786, found 243.0784.

*2-(m-Tolylethynyl)benzaldehyde* (**1E**). ^1^H-NMR (500 MHz, CDCl_3_) δ 10.66 (s, 1H), 7.95 (d, *J* = 7.8 Hz, 1H), 7.91 (d, *J* = 9.7 Hz, 1H), 7.64 (d, *J* = 7.5 Hz, 1H), 7.59 (td, *J* = 7.6, 1.1 Hz, 1H), 7.45 (t, *J* = 7.6 Hz, 1H), 7.44–7.35 (m, 3H), 7.29 (d, *J* = 7.6 Hz, 1H), 7.21 (d, *J* = 7.7 Hz, 1H), 2.38 (s, 3H). ^13^C-NMR (125 MHz, CDCl_3_) δ 191.78, 138.19, 135.70, 133.73, 133.11, 132.16, 129.92, 128.67, 128.45, 128.34, 127.15, 126.96, 122.02, 96.52, 84.44, 21.15. LRMS (ESI) *m/z* 221 [M+H]^+^; HRMS (ESI) *m/z* calcd C_16_H_12_ONa [M+Na]^+^ 243.0786, found 243.0791.

*5-Fluoro-2-(phenylethynyl)benzaldehyde* (**1F**). ^1^H-NMR (400 MHz, CDCl_3_) δ 10.63 (s, 1H), 7.71–7.62 (m, 2H), 7.61–7.53 (m, 2H), 7.43–7.41 (m, 3H), 7.38–7.28 (m, 1H). ^13^C-NMR (125 MHz, CDCl_3_) δ 190.52, 162.41 (d, *J* = 252.7 Hz), 137.78 (d, *J* = 6.6 Hz), 135.28 (d, *J* = 7.6 Hz), 131.67, 129.21, 128.60, 123.04 (d, *J* = 3.3 Hz), 122.13, 121.42 (d, *J* = 22.8 Hz), 113.73 (d, *J* = 23.0 Hz), 96.06, 83.84. LRMS (ESI) *m/z* 225 [M+H]^+^; HRMS (ESI) *m/z* calcd C_15_H_9_ONaF [M+Na]^+^ 247.0535, found 247.0529.

*4-Methyl-2-(phenylethynyl)benzaldehyde* (**1G**). ^1^H-NMR (400 MHz, CDCl_3_) δ 10.62 (s, 1H), 7.88 (d, *J* = 8.0 Hz, 1H), 7.64–7.55 (m, 2H), 7.49 (s, 1H), 7.46–7.36 (m, 3H), 7.30 (s, 1H), 2.45 (s, 3H). ^13^C-NMR (125 MHz, CDCl_3_) δ 191.43, 144.91, 133.69, 133.62, 131.70, 129.72, 129.03, 128.55, 127.36, 126.91, 122.45, 95.87, 85.13, 21.67. LRMS (ESI) *m/z* 221 [M+H]^+^; HRMS (ESI) *m/z* calcd C_16_H_12_ONa [M+Na]^+^ 243.0786, found 243.0786.

*2-(Oct-1-yn-1-yl)benzaldehyde* (**1H**). ^1^H-NMR (400 MHz, CDCl_3_) δ 10.56 (s, 1H), 7.91 (d, *J* = 8.0 Hz, 1H), 7.65–7.47 (m, 2H), 7.47–7.33 (m, 1H), 2.50 (t, *J* = 7.1 Hz, 2H), 1.73–1.59 (m, 2H), 1.54–1.44 (m, 2H), 1.42–1.31 (m, 4H), 0.93 (t, *J* = 6.8 Hz, 3H). ^13^C-NMR (125 MHz, CDCl_3_) δ 192.21, 135.96, 133.70, 133.29, 127.99, 127.84, 126.88, 98.24, 76.32, 31.32, 28.67, 28.50, 22.56, 19.61, 14.07. LRMS (ESI) *m/z* 215 [M+H]^+^; HRMS (ESI) *m/z* calcd C_15_H_18_ONa [M+Na]^+^ 237.1255, found 237.1259.

### 3.3. General Procedure for Synthesis of Dihydrobenzo [4,5][1,3]oxazino[2,3-a]isoquinolines *(**3Aa*** as an Example*)*

To a solution of 2-(phenylethynyl)benzaldehyde (**1A**, 0.1 mmol) in dry toluene (2 mL) were added (2-aminophenyl)methanol (**2a**, 0.12 mmol) and AgNO_3_ catalyst (5 mol%). Then, the reaction vial was sealed and the mixture was heated to 80 °C for 3 h. Afterwards, the cooled mixture was concentrated under reduced pressure, and the resulting reside was purified by flash column chromatography (PE/EA = 30/1, v/v, as an eluent) to afford the desired product **3Aa** [17] in 94% yield. ^1^H-NMR (400 MHz, CDCl_3_) *δ* 7.43 (d, *J* = 7.6 Hz, 1H), 7.34 (t, *J* = 7.2 Hz, 1H), 7.25–7.19 (m, 7H), 7.06 (d, *J* =7.6 Hz, 1H), 6.93 (t, *J* = 7.6 Hz, 1H), 6.80 (t, *J* = 8.4 Hz, 1H), 6.24 (d, *J* = 8.0 Hz, 1H), 6.10 (s, 1H), 5.97 (s, 1H), 5.27 (d, *J* = 14.4 Hz, 1H), 5.11 (d, *J* = 14.8 Hz, 1H). ^13^C-NMR (100 MHz, CDCl_3_) *δ* 140.75, 139.82, 136.90, 132.32, 128.96, 128.56, 128.41, 127.95, 127.82, 126.75, 125.97, 125.73, 124.66, 124.29, 123.74, 122.51, 105.63, 84.96, 69.99. LRMS (EI) *m/z* 311 (M^+^); HRMS (EI) *m/z* calcd C_22_H_17_NO (M^+^) 311.1310, found 311.1303. 

*8-Fluoro-12-phenyl-4b,6-dihydrobenzo[4,5][1,3]oxazino[2,3-a]isoquinoline* (**3Ab**). ^1^H-NMR (400 MHz, CDCl_3_) δ 7.40 (d, *J* = 7.2 Hz, 1H), 7.34 (t, *J* = 7.6Hz, 1H), 7.26–7.22 (m, 4H), 7.20–7.17 (m, 3H), 6.77 (dd, *J* = 8.4, 2.4 Hz, 1H), 6.51 (td, *J* = 8.8, 2.8 Hz, 1H), 6.19 (dd, *J* = 8.8, 4.8 Hz, 1H), 6.04 (s, 1H), 5.91 (s, 1H), 5.24 (d, *J* = 15.2 Hz, 1H), 5.07 (d, *J* = 15.2 Hz, 1H). ^13^C-NMR (100 MHz, CDCl_3_) δ 158.60 (d, *J* = 242.0 Hz), 140.87, 136.70, 136.01, 132.17, 130.13 (d, *J* = 7.4 Hz), 129.17, 128.74, 127.98, 127.89, 127.15, 125.93, 125.60 (d, *J* = 7.8 Hz), 124.34, 112.81 (d, *J* = 22.8 Hz), 111.15 (d, *J* = 22.9 Hz), 104.87, 84.98, 67.69. LRMS (EI) m/z 329 (M^+^); HRMS (EI) m/z calcd C_22_H_16_FNO (M^+^) 329.1216, found 329.1213.

*8-Chloro-12-phenyl-4b,6-dihydrobenzo[4,5][1,3]oxazino[2,3-a]isoquinoline* (**3Ac**). ^1^H-NMR (400 MHz, CDCl_3_) *δ* 7.41 (d, *J* = 7.6 Hz, 1H), 7.33 (td, *J* = 7.6, 1.2 Hz, 1H), 7.29–7.17 (m, 6H), 7.04 (d, *J* = 2.4 Hz, 1H), 6.75 (dd, *J* = 8.8, 2.4 Hz, 1H), 6.13 (d, *J* = 8.8 Hz, 1H), 6.05 (s, 1H), 6.0 (s, 1H), 5.19 (d, *J* = 14.4 Hz, 1H), 5.04 (d, *J* = 14.4 Hz, 1H). ^13^C-NMR (100 MHz, CDCl_3_) *δ* 140.19, 138.24, 136.43, 132.10, 129.55, 129.05, 128.46, 128.16, 128.05, 127.54, 126.80, 126.50, 126.24, 126.00, 124.61, 124.51, 124.40, 106.49, 85.05, 67.44. LRMS (EI) *m/z* 345 (M^+^); HRMS (EI) *m/z* calcd C_22_H_16_ClNO (M^+^) 345.0920, found 345.0919.

*9-Chloro-12-phenyl-4b,6-dihydrobenzo[4,5][1,3] oxazino[2,3-a]isoquinoline* (**3Ad**). ^1^H-NMR (300 MHz, CDCl_3_) *δ* 7.43 (d, *J* = 7.2 Hz, 1H), 7.36–7.26 (m, 6H), 7.23–7.18 (m, 2H), 6.97 (d, *J* = 8.1 Hz, 1H), 6.84 (dd, *J* = 8.1, 1.8 Hz, 1H), 6.16 (d, *J* =1.5 Hz, 1H), 6.11 (s, 1H), 6.07 (s, 1H), 5.16 (d, *J* = 14.1 Hz, 1H), 5.04 (d, *J* = 14.1 Hz, 1H). ^13^C-NMR (100 MHz, CDCl_3_) *δ* 139.88, 139.37, 135.60, 131.61, 131.20, 128.49, 127.85, 127.78, 127.29, 125.99, 125.70, 125.50, 125.06, 123.94, 122.11, 121.49, 107.48, 84.64, 66.85. LRMS (EI) *m/z* 345 (M^+^); HRMS (EI) *m/z* calcd C_22_H_16_ClNO (M^+^) 345.0920, found 345.0916.

*8-Bromo-12-phenyl-4b,6-dihydrobenzo[4,5][1,3] oxazino[2,3-a]isoquinoline* (**3Ae**). ^1^H-NMR (400 MHz, CDCl_3_) *δ* 7.41 (d, *J* = 8.0 Hz, 1H), 7.32 (td, *J* = 7.6, 1.2 Hz, 1H), 7.21–7.28 (m, 6H), 7.19–7.17 (m, 2H), 6.88 (dd, *J* = 8.4, 2.0 Hz,1H), 6.07 (s, 1H), 6.05 (s, 1H), 6.01(s, 1H), 5.18 (d, *J* = 14.8 Hz, 1H), 5.03 (d, *J* = 14.8 Hz, 1H). ^13^C-NMR (100 MHz, CDCl_3_) *δ* 140.07, 138.69, 136.38, 132.11, 129.89, 129.04, 128.93, 128.42, 128.21, 128.09, 127.55, 126.98, 126.39, 126.32, 124.71, 124.43, 115.04, 106.83, 85.07, 67.33. LRMS (EI) *m/z* 389 (M^+^); HRMS (EI) *m/z* calcd C_22_H_16_BrNO (M^+^) 389.0415, found 389.0410.

*8-Fluoro-12-(4-fluorophenyl)-4b,6-dihydrobenzo[4,5][1,3]oxazino[2,3-a]isoquinoline* (**3Bb**). ^1^H-NMR (400 MHz, CDCl_3_) *δ* 7.43 (d, *J* = 7.6 Hz, 1H), 7.37 (t, *J* = 7.2 Hz, 1H), 7.25–7.30 (m, 1H), 7.15–7.23 (m, 3H), 6.97 (t, *J* = 8.4 Hz, 2H), 6.8 (dd, *J* = 8.4, 2.4 Hz, 1H), 6.57 (td, *J* = 8.8, 2.4 Hz, 1H), 6.21 (dd, *J* = 8.8, 4.8 Hz, 1H), 6.05 (s, 1H), 5.90 (s, 1H), 5.27 (d, *J* = 15.2 Hz, 1H), 5.09 (d, *J* = 15.2 Hz, 1H). ^13^C-NMR (100 MHz, CDCl_3_) *δ* 162.39 (d, *J* = 246.6 Hz), 158.75 (d, *J* = 242.7 Hz), 139.96, 135.91, 132.84, 132.09, 130.57 (d, *J* = 8.0 Hz), 130.36 (d, *J* = 7.1 Hz), 129.27, 127.22, 126.11, 126.04, 125.71 (d, *J* = 8.1 Hz), 124.40, 115.09 (d, *J* = 21.4 Hz), 113.00 (d, *J* = 23.0 Hz), 111.34(d, *J* = 22.8 Hz), 105.09, 85.07, 67.74. LRMS (EI) *m/z* 347 (M^+^); HRMS (EI) *m/z* calcd C_22_H_15_F_2_NO (M^+^) 347.1122, found 347.1116.

*9-Chloro-12-(4-fluorophenyl)-4b,6-dihydrobenzo[4,5][1,3]oxazino[2,3-a]isoquinoline* (**3Bd**). ^1^H-NMR (400 MHz, CDCl_3_) *δ* 7.45 (d, *J* = 7.6 Hz, 1H), 7.36 (td, *J* = 7.6, 1.2 Hz,1H), 731–7.25 (m, 3H), 7.22 (d, *J* = 7.6 Hz, 1H), 7.05–6.98 (m, 3H), 6.89 (dd, *J* = 8.0, 2.0 Hz, 1H), 6.18 (d, *J* = 1.6 Hz, 1H), 6.10 (s, 1H), 6.07 (s, 1H), 5.16 (d, *J* = 14.4 Hz, 1H), 5.05 (d, *J* = 14.4 Hz, 1H). ^13^C-NMR (100 MHz, CDCl_3_) *δ* 162.63 (d, *J* = 246.8 Hz), 140.26, 138.91, 132.24 (d, *J* = 3.4 Hz), 132.00, 131.88, 130.05 (d, *J* = 8.2 Hz), 129.02, 127.76, 126.66, 126.26, 126.01, 125.75, 124.52, 122.67, 122.10, 115.40 (d, *J* = 21.6 Hz), 108.06, 85.08, 67.37. LRMS (EI) *m/z* 363 (M^+^); HRMS (EI) *m/z* calcd C_22_H_15_ClFNO (M^+^) 363.0826, found 363.0831.

*8-Bromo-12-(4-fluorophenyl)-4b,6-dihydrobenzo[4,5][1,3]oxazino[2,3-a]isoquinoline* (**3Be**). ^1^H-NMR (400 MHz, CDCl_3_) *δ* 7.45 (d, *J* = 7.6 Hz, 1H), 7.36 (td, *J* = 7.6, 1.2 Hz, 1H), 7.31–7.25 (m, 3H), 7.22 (d, *J* = 7.6 Hz, 1H), 7.05–6.98 (m, 3H), 6.89 (dd, *J* = 8.0, 2.0 Hz,1H), 6.18 (d, *J* = 1.6 Hz, 1H), 6.10 (s, 1H), 6.07 (s, 1H), 5.16 (d, *J* = 14.4 Hz, 1H), 5.05 (d, *J* = 14.4Hz, 1H). ^13^C-NMR (100 MHz, CDCl_3_) *δ* 162.49 (d, *J* = 246.8 Hz), 139.13, 138.57, 132.51 (d, *J* = 3.2 Hz), 132.01, 130.27, 130.19, 130.1, 129.09 (d, *J* = 6.4 Hz), 127.72, 126.99, 126.49, 126.46, 124.80, 124.47, 115.42 115.26 (d, *J* = 21.6 Hz), 106.91, 85.11, 67.35. LRMS (EI) *m/z* 407 (M^+^); HRMS (EI) *m/z* calcd C_22_H_15_BrFNO (M^+^) 407.0321, found 407.0320.

*12-(4-Fluorophenyl)-8-methyl-4b,6-dihydrobenzo[4,5][1,3]oxazino[2,3-a]isoquinoline* (**3Bf**). ^1^H-NMR (400 MHz, CDCl_3_) *δ* 7.40 (d, *J* = 7.6 Hz, 1H), 7.33 (t, *J* = 7.2 Hz, 1H), 7.26–7.14 (m, 4H), 6.93 (t, *J* = 8.8 Hz, 2H), 6.86 (s, 1H), 6.63 (d, *J* = 8.0 Hz, 1H), 6.12 (d, *J* = 8.4 Hz, 1H), 6.05 (s, 1H), 5.84 (s, 1H), 5.25 (d, *J* = 14.8 Hz, 1H), 5.06 (d, *J* = 14.8 Hz, 1H), 2.25 (s, 3H). ^13^C-NMR (100 MHz, CDCl_3_) *δ* 162.26 (d, *J* = 246.1 Hz), 140.12, 137.29, 133.14 (d, *J* = 3.7 Hz), 132.69, 132.20, 130.48 (d, *J* = 8.3 Hz), 129.06, 128.55, 127.19, 126.48, 126.10, 125.84, 125.16, 124.21, 124.07, 114.88 (d, *J* = 21.4 Hz), 104.47, 84.93, 68.05, 20.81. LRMS (EI) *m/z* 343 (M^+^); HRMS (EI) *m/z* calcd C_23_H_18_FNO (M^+^) 343.1372, found 343.1375.

*12-(4-Fluorophenyl)-10-methyl-4b,6-dihydrobenzo[4,5][1,3]oxazino[2,3-a]isoquinoline* (**3Bg**). ^1^H-NMR (400 MHz, CDCl_3_) *δ* 7.38–7.36 (m, 2H), 7.26–7.20 (m, 2H), 7.10–6.92 (m, 4H), 6.87–6.77 (m, 3H), 5.91–5.90 (m, 2H), 5.30–5.17 (m, 2H), 1.67 (s, 3H). ^13^C-NMR (125 MHz, CDCl_3_) *δ* 162.21 (d, *J* = 246.3 Hz), 141.92, 140.45, 133.85, 133.35, 132.50, 130.99, 130.16 (d, *J* = 8.0 Hz), 129.36, 128.50, 127.80, 125.66 (d, *J* = 33.1 Hz), 125.67, 124.41, 122.70, 114.53, 114.36, 104.47, 85.02, 67.75, 17.34. LRMS (EI) *m/z* 343 (M^+^); HRMS (EI) *m/z* calcd C_23_H_18_FNO (M^+^) 343.1372, found 343.1368.

*12-(4-Chlorophenyl)-4b,6-dihydrobenzo[4,5][1,3]oxazino[2,3-a]isoquinoline* (**3Ca**). ^1^H-NMR (400 MHz, CDCl_3_) *δ* 7.42 (d, *J* = 7.6 Hz, 1H), 7.33 (t, *J* = 7.2 Hz, 1H), 7.27–7.14 (m, 6H), 7.06 (d, *J* = 7.2 Hz, 1H), 6.95 (t, *J* = 7.6 Hz, 1H), 6.84 (t, *J* = 7.6 Hz, 1H), 6.22 (d, *J* = 8.4 Hz, 1H), 6.08 (s, 1H), 5.95 (s, 1H), 5.26 (d, *J* = 14.4 Hz, 1H), 5.09 (d, *J* = 14.8 Hz, 1H). ^13^C-NMR (100 MHz, CDCl3) *δ* 139.52, 135.38, 133.71, 132.04, 129.81, 129.02, 128.44, 128.22, 126.76, 126.23, 125.95, 124.81, 124.37, 123.67, 122.73, 106.07, 84.91, 67.97. LRMS (EI) *m/z* 345 (M^+^); HRMS (EI) *m/z* calcd C_22_H_16_ClNO (M^+^) 345.0920, found 345.0890.

*8-Chloro-12-(4-chlorophenyl)-4b,6-dihydrobenzo[4,5][1,3]oxazino[2,3-a]isoquinoline* (**3Cc**). ^1^H-NMR (400 MHz, CDCl_3_) *δ* 7.41 (d, *J* = 7.6 Hz, 1H), 7.34 (td, *J* = 7.2, 1.2 Hz, 1H), 7.28–7.23 (m, 3H), 7.20–7.14 (m, 3H), 7.06 (d, *J* = 2.0 Hz, 1H), 6.80 (dd, *J* = 8.8, 2.4 Hz, 1H), 6.12 (d, *J* = 8.4 Hz, 1H), 6.04 (s, 1H), 5.99 (s, 1H), 5.18 (d, *J* = 14.8 Hz, 1H), 5.04 (d, *J* = 14.8 Hz, 1H). ^13^C-NMR (100 MHz, CDCl3) *δ*138.99, 137.96, 134.93, 133.99, 131.85, 129.71, 129.62, 129.12, 128.45, 127.80, 126.92, 126.54, 126.51, 126.24, 124.79, 124.51, 124.42, 107.02, 85.05, 67.45. LRMS (EI) *m/z* 379 (M^+^); HRMS (EI) *m/z* calcd C_22_H_15_Cl_2_NO (M^+^) 379.0531, found 379.0503.

*9-Chloro-12-(4-chlorophenyl)-4b,6-dihydrobenzo[4,5][1,3]oxazino[2,3-a]isoquinoline* (**3Cd**). ^1^H-NMR (400 MHz, CDCl_3_) *δ* 7.42 (d, *J* = 7.6 Hz, 1H), 7.33 (td, *J* = 7.2, 1.2 Hz, 1H), 7.29–7.25 (m, 3H), 7.23–7.17 (m, 3H), 6.97 (d, *J* = 8.0 Hz, 1H), 6.86 (dd, *J* = 8.0, 2.0 Hz, 1H), 6.15 (d, *J* = 1.6 Hz, 1H), 6.11 (s, 1H), 6.04 (s, 1H), 5.12 (d, *J* = 14.4 Hz, 1H), 5.02 (d, *J* = 14.4 Hz, 1H). ^13^C-NMR (100 MHz, CDCl_3_) *δ* 140.08, 138.61, 134.56, 134.15, 131.96 131.82, 129.37, 128.96, 128.57, 127.94, 126.82, 126.08, 125.83, 125.74, 124.57, 122.35, 122.00, 108.72, 84.99, 67.26. LRMS (EI) *m/z* 379 (M^+^); HRMS (EI) *m/z* calcd C_22_H_15_Cl_2_NO (M^+^) 379.0531, found 379.0508.

*12-(p-Tolyl)-4b,6-dihydrobenzo[4,5][1,3]oxazino[2,3-a]isoquinoline* (**3Da**). ^1^H-NMR (400 MHz, CDCl_3_) *δ* 7.41 (d, *J* = 7.6, 1H), 7.31 (td, *J* = 7.6, 1.2 Hz, 1H), 7.23 (td, *J* = 7.2, 1.2 Hz, 1H), 7.17 (d, *J* = 7.6 Hz, 1H), 7.12 (d, *J* = 8.0, 2H), 7.05 (m, 3H), 6.92 (td, *J* = 7.6, 1.2 Hz, 1H), 6.81 (td, *J* = 8.4, 1.2 Hz, 1H), 6.25 (d, *J* = 8.0, 1H), 6.08 (s, 1H), 5.94 (s, 1H), 5.25 (d, *J* = 14.8, 1H), 5.09 (d, *J* = 14.8, 1H), 2.33 (s, 3H). ^13^C-NMR (100 MHz, CDCl3) *δ* 140.77, 139.87, 137.69, 133.96, 132.44, 128.91, 128.66, 128.41, 128.35, 126.71, 126.63, 125.85, 125.77, 124.62, 124.20, 123.74, 122.37, 105.42, 84.98, 67.96, 21.26. LRMS (EI) *m/z* 325 (M^+^); HRMS (EI) *m/z* calcd C_23_H_19_NO (M^+^) 325.1467, found 325.1472.

*8-Chloro-12-(p-tolyl)-4b,6-dihydrobenzo[4,5][1,3]oxazino[2,3-a]isoquinoline* (**3Dc**). ^1^H-NMR (300 MHz, CDCl_3_) *δ* 7.41 (d, *J* = 10.0 Hz, 1H), 7.32 (td, *J* = 10.0, 1.6 Hz, 1H), 7.26–7.04 (m, 7H), 6.77 (dd, *J* = 10.6, 3.2 Hz, 1H), 6.16 (d, *J* = 10.6 Hz, 1H), 6.05 (s, 1H), 5.99 (s, 1H), 5.18 (d, *J* = 19.6 Hz, 1H), 5.04 (d, *J* = 19.2 Hz, 1H), 2.35 (s, 3H). ^13^C-NMR (100 MHz, CDCl3) *δ* 140.27, 138.36, 138.04, 133.56, 132.31, 129.53, 129.03, 128.92, 128.36, 127.42, 127.00, 126.41, 126.17, 126.11, 124.61, 124.53, 124.37, 106.37, 85.17, 67.45, 21.30. LRMS (EI) *m/z* 359 (M^+^); HRMS (EI) *m/z* calcd C_23_H_18_ClNO (M^+^) 359.1077, found 359.1078.

*9-Chloro-12-(p-tolyl)-4b,6-dihydrobenzo[4,5][1,3]oxazino[2,3-a]isoquinoline* (**3Dd**). ^1^H-NMR (400 MHz, CDCl_3_) *δ* 7.42 (dd, *J* = 8.8, 0.8 Hz, 1H), 7.32 (td, *J* = 7.6, 1.2 Hz, 1H), 7.25 (td, *J* = 7.2, 1.2 Hz, 1H), 7.20–7.16 (m, 3H), 7.10 (d, *J* = 8.0 Hz, 2H), 6.96 (d, *J* = 8.0 Hz, 1H), 6.83 (dd, *J* = 8.0, 1.6 Hz, 1H), 6.18 (d, *J* = 2.0 Hz, 1H), 6.11 (s, 1H), 6.05 (s, 1H), 5.13 (d, *J* = 14.0 Hz, 1H), 5.02 (d, *J* = 14.0 Hz, 1H), 2.35 (s, 3H). ^13^C-NMR (100 MHz, CDCl_3_) *δ* 140.42, 139.90, 138.22, 133.13, 132.25, 131.75, 129.00, 128.85, 128.03, 127.90, 126.38, 125.98, 125.74, 125.52, 124.38, 122.49, 121.64, 107.79, 85.04, 67.25, 21.27. LRMS (EI) *m/z* 359 (M^+^); HRMS (EI) *m/z* calcd C_23_H_18_ClNO (M^+^) 359.1077, found 359.1053.

*8-Bromo-12-(p-tolyl)-4b,6-dihydrobenzo[4,5][1,3]oxazino[2,3-a]isoquinoline* (**3De**). ^1^H-NMR (400 MHz, CDCl_3_) *δ* 7.42 (d, *J* = 7.6 Hz, 1H), 7.32 (td, *J* = 7.6, 1.2 Hz, 1H), 7.24 (td, *J* = 7.2, 1.2 Hz, 1H), 7.86 (d, *J* = 8.0 Hz, 2H), 7.14 (d, *J* = 8.0 Hz, 2H), 7.08 (d, *J* = 8.0 Hz, 2H), 6.91 (dd, *J* = 8.4, 2.0 Hz, 1H), 6.10 (d, *J* = 8.8 Hz, 1H), 6.04 (s, 1H), 6.02 (s, 1H), 5.17 (d, *J* = 14.4 Hz, 1H), 5.03 (d, *J* = 14.8 Hz, 1H), 2.35 (s, 3H). ^13^C-NMR (100 MHz, CDCl_3_) *δ* 140.07, 138.72, 138.00, 133.44, 132.25, 129.80, 128.95, 128.91, 128.25, 127.47, 127.10, 126.23, 126.17, 124.66, 124.32, 114.83, 106.64, 85.09, 67.25, 21.25. LRMS (EI) *m/z* 403 (M^+^); HRMS (EI) *m/z* calcd C_23_H_18_BrNO (M^+^) 403.0572, found 403.0558.

*8-Methyl-12-(p-tolyl)-4b,6-dihydrobenzo[4,5][1,3]oxazino[2,3-a]isoquinoline* (**3Df**). ^1^H-NMR (400 MHz, CDCl_3_) *δ* 7.40 (d, *J* = 7.6 Hz, 1H), 7.32 (td, *J* = 7.6, 0.8 Hz, 1H), 7.22 (td, *J* = 7.6, 1.2 Hz, 1H), 7.17 (d, *J* = 7.6 Hz, 1H), 7.11 (d, *J* = 7.6 Hz, 2H), 7.05 (d, *J* = 8.0 Hz, 2H), 6.86 (s, 1H), 6.62 (d, *J* = 7.2 Hz, 1H), 6.17 (d, *J* = 8.0 Hz, 1H), 6.06 (s, 1H), 5.87 (s, 1H), 5.25 (d, *J* = 14.8 Hz, 1H), 5.07 (d, *J* = 14.8 Hz, 1H), 2.34 (s, 3H), 2.25 (s, 3H). ^13^C-NMR (100 MHz, CDCl_3_) *δ* 141.07, 137.56, 137.53, 134.15, 132.48, 132.24, 128.93, 128.56, 128.36, 127.01, 126.43, 126.23, 125.58, 125.01, 124.12, 123.95, 104.29, 84.98, 68.01, 21.25, 20.83. LRMS (EI) *m/z* 339 (M^+^); HRMS (EI) *m/z* calcd C_24_H_21_NO (M^+^) 339.1623, found 339.1609.

*14-(p-Tolyl)-4b,6-dihydronaphtho[2′,3′:4,5][1,3]oxazino[2,3-a]isoquinoline* (**3Dh**). ^1^H-NMR (400 MHz, CDCl_3_) *δ* 7.70–7.66 (m, 1H), 7.54 (s, 1H), 7.47 (d, *J* = 7.6 Hz, 1H), 7.34–7.30 (m, 3H), 7.28–7.21 (m, 5H), 7.06 (d, *J* = 7.6 Hz, 2H), 6.53 (s, 1H), 6.36 (s, 1H), 6.20 (s, 1H), 5.25 (d, *J* = 13.6 Hz, 1H), 5.23 (d, *J* = 13.6 Hz, 1H), 2.30 (s, 3H). ^13^C-NMR (100 MHz, CDCl_3_) *δ* 140.13, 137.98, 136.77, 133.25, 132.84, 132.49, 129.39, 129.02, 128.59, 128.45, 128.07, 127.74, 127.15 , 126.80, 126.61, 125.61, 124.50, 124.20, 123.94, 123.06, 117.77, 109.94, 85.75, 67.43, 21.20. LRMS (EI) *m/z* 375 (M^+^); HRMS (EI) *m/z* calcd C_27_H_21_NO (M^+^) 375.1623, found 375.1621.

*12-(m-Tolyl)-4b,6-dihydrobenzo[4,5][1,3]oxazino[2,3-a]isoquinoline* (**3Ea**). ^1^H-NMR (400 MHz, CDCl_3_) *δ* 7.43 (d, *J* = 7.2 Hz, 1H), 7.34 (t, *J* = 7.2 Hz, 1H), 7.26–7.18 (m, 2H), 7.14–7.04 (m, 4H), 6.99–6.91 (m, 2H), 6.82 (t, *J* = 7.6 Hz, 1H), 6.26 (d, *J* = 8.0 Hz, 1H), 6.10 (s, 1H), 5.97 (s, 1H), 5.27 (d, *J* = 14.4 Hz, 1H), 5.11 (d, *J* = 14.4 Hz, 1H), 2.30 (s, 3H). ^13^C-NMR (100 MHz, CDCl_3_) *δ* 140.89, 139.82, 137.60, 136.77, 132.36, 129.12, 128.90, 128.54, 128.28, 127.69, 126.70, 125.85, 125.72, 125.65, 124.58, 124.21, 123.69, 122.42, 105.44, 84.93, 67.93, 21.34. LRMS (EI) *m/z* 325 (M^+^); HRMS (EI) *m/z* calcd C_23_H_19_NO (M^+^) 325.1467, found 325.1468.

*8-Chloro-12-(m-tolyl)-4b,6-dihydrobenzo[4,5][1,3]oxazino[2,3-a]isoquinoline* (3Ec). ^1^H-NMR (400 MHz, CDCl_3_) *δ* 7.41 (d, *J* = 7.6 Hz, 1H), 7.33 (t, *J* = 7.2 Hz, 1H), 7.26–7.10 (m, 5H), 7.04 (s, 1H), 6.96 (d, *J* = 6.8 Hz, 1H), 6.76 (dd, *J* = 8.4, 1.2 Hz, 1H), 6.15 (d, *J* = 8.8 Hz, 1H), 6.05 (s, 1H), 5.99 (s, 1H), 5.19 (d, *J* = 14.4 Hz, 1H), 5.04 (d, *J* = 14.8 Hz, 1H), 2.31 (s, 3H). ^13^C-NMR (100 MHz, CDCl_3_) *δ* 140.35, 138.30, 137.90, 136.37, 132.19, 129.42, 129.00, 128.82, 127.94, 127.40, 126.90, 126.43, 126.18, 125.99, 125.65, 124.55, 124.43, 124.36, 106.46, 85.09, 67.42, 21.41. LRMS (EI) *m/z* 359 (M^+^); HRMS (EI) *m/z* calcd C_23_H_18_ClNO (M^+^) 359.1077, found 359.1063.

*8-Bromo-12-(m-tolyl)-4b,6-dihydrobenzo[4,5][1,3]oxazino[2,3-a]isoquinoline* (**3Ee**). ^1^H-NMR (400 MHz, CDCl3) *δ* 7.41 (d, *J* = 7.6 Hz, 1H), 7.33 (td, *J* = 7.2, 1.2 Hz,1H), 7.25 (td, *J* = 7.6, 1.2 Hz,1H), 7.20–7.08 (m, 5H), 6.97 (d, *J* = 7.2Hz, 1H), 6.90 (dd, *J* = 8.8, 2.0 Hz, 1H), 6.09 (d, *J* = 8.8 Hz, 1H), 6.05 (s, 1H), 6.02 (s, 1H), 5.18 (d, *J* = 14.8 Hz, 1H), 5.04 (d, *J* = 15.6 Hz, 1H), 2.3 (s, 3H). ^13^C-NMR (100 MHz, CDCl3) *δ* 140.20, 138.73, 137.93, 136.31, 132.18, 129.71, 128.98, 128.91, 128.85, 127.94, 127.48, 127.08, 126.30, 126.23, 125.59, 24.60, 124.37, 114.87, 106.80, 85.08, 67.27, 21.42. LRMS (EI) *m/z* 403 (M^+^); HRMS (EI) *m/z* calcd C_23_H_18_BrNO (M^+^) 403.0572, found 403.0576.

*3-Fluoro-12-phenyl-4b,6-dihydrobenzo[4,5][1,3]oxazino[2,3-a]isoquinoline* (**3Fa**). ^1^H-NMR (400 MHz, CDCl_3_) *δ* 7.29–7.27 (m, 5H), 7.20–7.16 (m, 2H), 7.10–7.02 (m, 2H), 6.93 (t, *J* = 7.6 Hz, 1H), 6.82 (t, *J* = 7.6 Hz, 1H), 6.24 (d, *J* = 8.0 Hz, 1H), 6.09 (s, 1H), 6.04 (s, 1H), 5.24 (d, *J* = 14.4 Hz, 1H), 5.10 (d, *J* = 14.4 Hz, 1H). ^13^C-NMR (100 MHz, CDCl_3_) *δ* 161.49 (d, *J* = 243.5 Hz), 140.01, 139.38, 136.64, 129.23, 129.13 (d, *J* = 7.6 Hz), 128.73, 128.38, 128.11, 127.99, 126.13, 125. 86 (d, *J* = 7.9 Hz), 124.70, 123.18, 122.24, 116.09 (d, *J* = 22.0 Hz), 113.16 (d, *J* = 22.6 Hz), 105.86, 84.67, 67.86. LRMS (EI) *m/z* 329 (M^+^); HRMS (EI) *m/z* calcd C_22_H_16_FNO (M^+^) 329.1216, found 329.1210.

*8-Chloro-3-fluoro-12-phenyl-4b,6-dihydrobenzo[4,5][1,3]oxazino[2,3-a]isoquinoline* (**3Fc**). ^1^H-NMR (400 MHz, CDCl_3_) *δ* 7.30–7.23 (m, 5H), 7.18–7.13 (m, 2H), 7.06–7.00 (m, 2H), 6.76 (dd, *J* = 8.8, 2.4 Hz, 1H), 6.11 (d, *J* = 8.8 Hz,1H), 6.06 (s, 1H), 6.02 (s, 1H), 5.14 (d, *J* = 14.4 Hz, 1H), 5.02 (d, *J* = 14.4 Hz, 1H). ^13^C-NMR (100 MHz, CDCl3) *δ* 162.80 (d, *J* = 244.2 Hz), 139.40, 137.77, 136.13, 129.25 (d, *J* = 7.5 Hz), 129.06, 128.48, 128.45, 128.28, 128.22, 127.20, 126.34, 125.98 (d, *J* = 7.7 Hz), 124.64, 123.85, 116.12 (d, *J* = 21.9 Hz), 112.96 (d, *J* = 22.9 Hz), 106.75, 84.71, 67.25. LRMS (EI) *m/z* 363 (M^+^); HRMS (EI) *m/z* calcd C_22_H_15_ClFNO (M^+^) 363.0826, found 363.0808.

*8-Bromo-3-fluoro-12-phenyl-4b,6-dihydrobenzo[4,5][1,3]oxazino[2,3-a]isoquinoline* (**3Fe**). ^1^H-NMR (400 MHz, CDCl_3_) *δ* 7.30–7.24 (m, 5H), 7.20 (d, *J* = 2.4Hz, 1H), 7.17–7.13 (m, 2H), 7.04 (td, *J* = 8.4, 2.4 Hz,1H), 6.90 (dd, *J* = 8.8, 2.4 Hz, 1H), 6.09 (s, 1H), 6.05 (d, *J* = 8.8 Hz, 1H), 6.02 (s, 1H), 5.13 (d, *J* = 14.0 Hz, 1H), 5.08–4.96 (d, *J* = 14.4 Hz, 1H). ^13^C-NMR (100 MHz, CDCl_3_) *δ* 161.64 (d, *J* = 244.1 Hz), 139.25, 138. 19, 136.06, 129.46 (d, *J* = 7.2 Hz), 129.38, 129.26, 128.42, 128.32, 128.22, 128.16, 127.54, 125.98 (d, *J* = 7.9 Hz), 124.02, 116.07 (d, *J* = 22.0 Hz), 114.62, 112.85 (d, *J* = 23.0 Hz), 107.12, 84.71, 67.10. LRMS (EI) *m/z* 407 (M^+^); HRMS (EI) *m/z* calcd C_22_H_15_BrFNO (M^+^) 407.0321, found 407.0316.

*3-Fluoro-8-methyl-12-phenyl-4b,6-dihydrobenzo[4,5][1,3]oxazino[2,3-a]isoquinoline* (**3Ff**). ^1^H-NMR (400 MHz, CDCl_3_) *δ* 7.26–7.19 (m, 5H), 7.16–7.11 (m, 2H), 7.02 (td, *J* = 8.4, 2.4 Hz, 1H), 6.86 (s, 1H), 6.60 (d, *J* = 8.4 Hz, 1H), 6.11 (d, *J* = 8.4 Hz, 1H), 6.03 (s, 1H), 5.91 (s, 1H), 5.21 (d, *J* =14.8 Hz, 1H), 5.05 (d, *J* = 14.8 Hz, 1H), 2.23 (s, 3H). ^13^C-NMR (100 MHz, CDCl3) *δ* 160.78 (d, *J* = 243.8 Hz), 140.30, 137.07, 136.80, 132.22, 128.86, 128.74, 128.54, 128.29 (d, *J* = 7.8 Hz), 127.97, 127.84, 126.66, 125.77 (d, *J* = 7.7 Hz), 125.10, 123.50, 116.18 (d, *J* = 22.8 Hz), 113.48 (d, *J* = 22.3 Hz), 104.42, 84.59, 67.94, 20.80. LRMS (EI) *m/z* 343 (M^+^); HRMS (EI) *m/z* calcd C_23_H_18_FNO (M^+^) 343.1372, found 343.1375.

*2-Methyl-12-phenyl-4b,6-dihydrobenzo[4,5][1,3]oxazino[2,3-a]isoquinoline* (**3Ga**). ^1^H-NMR (400 MHz, CDCl_3_) *δ* 7.31 (d, *J* = 8.0 Hz, 1H), 7.26–7.19 (m, 5H), 7.07–7.03 (m, 2H), 7.00 (s, 1H), 6.92 (t, *J* = 7.6Hz, 1H), 6.78 (t, *J* = 7.6Hz, 1H), 6.22 (d, *J* = 8.4 Hz, 1H), 6.07 (s, 1H), 5.90 (s, 1H), 5.26 (d, *J* =14.8 Hz,1H), 5.09 (d, *J* =14.4 Hz,1H), 2.36 (s, 3H). ^13^C-NMR (125 MHz, CDCl_3_) *δ* 140.81, 140.05, 138.74, 137.11, 132.24, 128.65, 128.52, 127.97, 127.79, 126.94, 126.78, 125.69, 124.79, 124.70, 124.12, 123.88, 122.54, 105.59, 85.00, 68.00, 21.40. LRMS (EI) *m/z* 325 (M^+^); HRMS (EI) *m/z* calcd C_23_H_19_NO (M^+^) 325.1467, found 325.1463

*8-Chloro-2-methyl-12-phenyl-4b,6-dihydrobenzo[4,5][1,3]oxazino[2,3-a]isoquinoline* (**3Gc**). ^1^H-NMR (400 MHz, CDCl_3_) *δ* 7.32–7.25 (m, 4H), 7.23–7.21 (m, 2H), 7.08 (d, *J* = 8.0 Hz, 1H), 7.04 (d, *J* = 1.6 Hz, 1H), 7.01 (s, 1H), 6.75 (dd, *J* = 8.8, 2.4 Hz, 1H), 6.13 (d, *J* = 8.8 Hz, 1H), 6.02 (s, 1H), 5.95 (s, 1H), 5.18 (d, *J* =14.8 Hz,1H), 5.04 (d, *J* =14.8 Hz,1H), 2.34 (s, 3H). ^13^C-NMR (125 MHz, CDCl_3_) *δ* 140.27, 138.87, 138.51, 136.68, 132.07, 129.69, 128.56, 128.21, 128.05, 127.60, 127.21, 126.58, 125.99, 124.95, 124.67, 124.23, 106.49, 85.14, 67.49, 21.41. LRMS (EI) *m/z* 359 (M^+^); HRMS (EI) *m/z* calcd C_23_H_18_ClNO (M^+^) 359.1077, found 359.1058.

*2,8-Dimethyl-12-phenyl-4b,6-dihydrobenzo[4,5][1,3]oxazino[2,3-a]isoquinoline* (**3Gf**). ^1^H-NMR (400 MHz, CDCl_3_) *δ* 7.31–7.28 (d, *J* = 8.0 Hz, 1H), 7.26–7.18 (m, 5H), 7.04 (d, *J* = 7.6 Hz, 1H), 6.99 (s, 1H), 6.85 (s, 1H), 6.59 (d, *J* = 7.6 Hz, 1H), 6.12 (d, *J* = 8.0 Hz, 1H), 6.04 (s, 1H), 5.81 (s, 1H), 5.25 (d, *J* = 14.8 Hz,1H), 5.06 (d, *J* = 14.8 Hz,1H), 2.36 (s, 3H), 2.24(s, 3H). ^13^C-NMR (125 MHz, CDCl_3_) *δ* 141.11, 138.76, 137.73, 137.30, 132.43, 132.29, 128.82, 128.52, 127.89, 127.69, 127.14, 126.73, 126.36, 125.10, 124.69, 124.08, 123.59, 104.46, 84.99, 68.06, 21.42, 20.87. LRMS (EI) *m/z* 339 (M^+^); HRMS (EI) *m/z* calcd C_24_H_21_NO (M^+^) 339.1623, found 339.1617.

*2-Methyl-14-phenyl-4b,6-dihydronaphtho[2′,3′:4,5][1,3]oxazino[2,3-a]isoquinoline* (**3Gh**). ^1^H-NMR (400 MHz, CDCl_3_) *δ* 7.68 (d, *J* = 8.4 Hz,1H), 7.54 (s, 1H), 7.42–7.36 (m, 3H), 7.27–7.25 (m, 6H), 7.10 (d, *J* = 7.6 Hz, 1H), 7.07 (s, 1H), 6.51 (s, 1H), 6.31 (s, 1H), 6.21 (s, 1H), 5.28 (d, *J* = 13.6 Hz, 1H), 5.22 (d, *J* = 14.0 Hz, 1H), 2.39 (s, 3H). ^13^C-NMR (125 MHz, CDCl_3_) *δ* 140.27, 138.34, 137.00, 136.43, 132.78, 132.32, 128.77, 128.33, 128.17, 128.10, 128.03, 127.60, 127.20, 126.88, 126.53, 125.76, 124.98, 124.87, 124.12, 123.19, 118.14, 109.99, 85.88, 67.63, 21.39. LRMS (EI) *m/z* 375 (M^+^); HRMS (EI) *m/z* calcd C_27_H_21_NO (M^+^) 375.1623, found 375.1617.

*12-Hexyl-4b,6-dihydrobenzo[4,5][1,3]oxazino[2,3-a]isoquinoline* (**3Ha**). ^1^H-NMR (400 MHz, CDCl_3_) *δ* 7.35– 7.31 (m, 3H), 7.27–7.09 (m, 5H), 5.83(s, 1H), 5.75(s, 1H), 5.23 (d, *J* = 15.6 Hz, 1H), 5.00 (d, *J* = 15.2 Hz, 1H), 2.57–2.36 (m, 2H), 1.46–1.31 (m, 2H), 1.25–1.13 (m, 6H), 0.85 (t, *J* = 6.4 Hz, 3H). ^13^C-NMR (100 MHz, CDCl_3_) *δ* 142.63, 140.91, 132.49, 130.91, 129.23, 127.63, 125.91, 125.31, 125.15, 125.00, 124.92, 124.85, 123.62, 100.42, 84.85, 67.74, 32.88, 31.52, 28.75, 27.57, 22.43, 14.02. LRMS (EI) *m/z* 319 (M^+^); HRMS (EI) *m/z* calcd C_22_H_25_NO (M^+^) 319.1936, found 319.1935.

*12-Hexyl-8-iodo-4b,6-dihydrobenzo[4,5][1,3]oxa-zino[2,3-a]isoquinoline* (**3Hi**). ^1^H-NMR (300 MHz, CDCl_3_) *δ* 7.54 (d, *J* =8.7 Hz, 1H), 7.43 (s, 1H), 7.34–7.27 (m, 2H), 7.19–7.10 (m, 2H), 6.92 (d, *J* = 8.4 Hz, 1H), 5.74 (s, 2H), 5.14 (d, *J* = 15.3 Hz,1H), 4.91 (d, *J* =15.9 Hz, 1H), 2.43–2.40 (m, 2H), 1.30–1.17 (m, 8H), 0.88–0.84 (m, 3H). ^13^C-NMR (125 MHz, CDCl_3_) *δ* 141.92, 140.66, 134.94, 134.27, 133.07, 132.26, 129.39, 127.60, 126.78, 125.19, 124.88, 123.77, 101.09, 84.83, 67.02, 32.73, 31.51, 28.74, 27.59, 22.46, 14.07. LRMS (EI) *m/z* 445 (M^+^); HRMS (EI) *m/z* calcd C_22_H_24_INO (M^+^) 445.0903, found 445.0905.

*6-Phenyl-11b,13-dihydropyrido[2′,3′:4,5][1,3]oxa-zino[2,3-a]isoquinoline* (**3Aj**). ^1^H-NMR (500 MHz, CDCl_3_) *δ* 7.81 (d, *J* = 5.5 Hz, 1H), 7.46 (d, *J* = 7.5 Hz, 1H), 7.36–7.28 (m, 6H), 7.26–7.19 (m, 3H), 6.71 (m, 1H), 6.34 (s, 1H), 6.23 (s, 1H), 5.09 (d, *J* = 14.0 Hz, 1H), 5.03 (d, *J* = 13.5 Hz, 1H). ^13^C-NMR (125 MHz, CDCl_3_) *δ* 150.45, 146.85, 139.24, 137.51, 132.44, 132.28, 129.63, 128.69, 128.04, 127.49, 127.06, 126.87, 124.91, 124.52, 121.24, 116.31, 111.25, 85.88, 66.45. LRMS (EI) *m/z* 312 (M^+^); HRMS (EI) *m/z* calcd C_21_H_16_N_2_O (M^+^) 312.1263, found 312.1260. 

*6-(4-Fluorophenyl)-11b,13-dihydropyrido[2′,3′:4,5][1,3]oxazino[2,3-a]isoquinoline* (**3Bj**). ^1^H-NMR (400 MHz, CDCl_3_) *δ* 7.81 (dd, *J* = 4.8, 1.6 Hz, 1H), 7.44 (d, *J* = 7.2 Hz, 1H), 7.34–7.26 (m, 4H), 7.25–7.22 (m, 1H), 7.18 (dd, *J* = 7.6, 1.2 Hz, 1H), 6.94 (t, *J* = 8.8 Hz, 2H), 6.74–6.70 (q, 1H), 6.26 (s, 1H), 6.20 (s, 1H), 5.06 (d, *J* = 14.0 Hz, 1H), 5.04–4.98 (d, *J* = 14.0 Hz, 1H). ^13^C-NMR (100 MHz, CDCl_3_) *δ* 150.19, 146.75, 138.22, 133.53, 132.39, 132.20, 129.38, 128.72, 128.65, 126.89, 124.70 (d, *J* = 25.6 Hz), 121.32, 116.46, 114.94 (d, *J* = 21.5 Hz), 110.90, 85.78, 66.37. LRMS (EI) *m/z* 330 (M^+^); HRMS (EI) *m/z* calcd _C21_H_15_FN_2_O (M^+^) 330.1168, found 330.1162.

## 4. Conclusions

In summary, an efficient strategy for the one-pot construction of dihydrobenzo[4,5][1,3]oxazino-[2,3-*a*]isoquinolines from simple starting substrates has been developed via an unprecedented Ag(I)-catalyzed *N*-aryliminium ion cyclization cascade. On the basis of a large assortment of bioactivities of fused isoquinolines, we believe that the new synthetic method will serve as valuable tool to efficiently construct new members fused isoquinoline derivatives in drug discovery, and will have potential biological applications.

## Figures and Tables

**Table 1:**
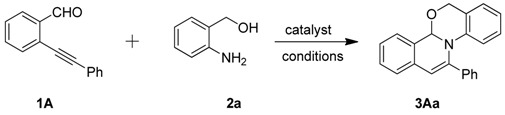
Optimization of the reaction conditions ^a^.

Entry	Catalyst system *^b^*	Solvent	Temp (°C)	Yield (%)
1	AuCl(PPh_3_)	Toluene	100	75
2	AuCl	Toluene	100	49
3	Au catalyst I	Toluene	100	58
4	Au catalyst II	Toluene	100	28
5	AgOOCCF_3_	Toluene	100	60
**6**	**AgNO_3_**	**Toluene**	**100**	**93**
7	AgBF_4_	Toluene	100	57
8	AgOTf	Toluene	100	92
9	AgNO_3_	MeOH	reflux	77
10	AgNO_3_	THF	reflux	60
11	AgNO_3_	DMF	100	71
12	AgNO_3_	DMSO	100	92
13	AgNO_3_	CH_3_CN	reflux	87
14	AgNO_3_	Dioxane	100	83
**15**	**AgNO_3_**	**Toluene**	**80**	**94**
16	AgNO_3_	DMSO	80	75
17	AgNO_3_	CH_3_CN	80	78
18	AgNO_3_	Dioxane	80	77
19	AgNO_3_	Toluene	50	65
20	AgNO_3_	Toluene	RT	30
**21**	**AgNO_3_**	**Toluene**	**80**	**94 ^c^**

^a^ **1A** (0.1 mmol), **2a** (0.2 mmol) and catalyst (5 mol%) in the specified solvent (2 mL) were reacted in a sealed vial; ^b^ Au catalyst I = (acetonitrile)[(2-biphenyl)di-*tert*-butylphosphine]gold (I) hexafluoroantimonate; Au catalyst II = 2-dicyclohexylphosphino-2′,4′,6′-triisopropyl-biphenyl gold(I) bis(trifluoromethane-sulfonyl)imide; ^c^ 1.2 eqv. of 2a was used.

**Table 2:**
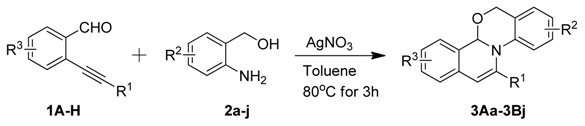
Silver-mediated one-pot domino synthesis of target compounds **3** ^a^.

Entry	Product		Yield (%)	Entry	Product		Yield (%)
1	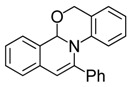	**3Aa**	94	18	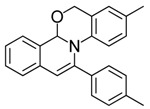	**3Df**	65
2	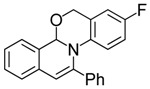	**3Ab**	92	19	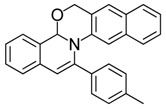	**3Dh**	70
3	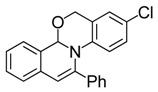	**3Ac**	85	20	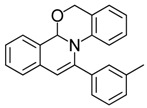	**3Ea**	87
4	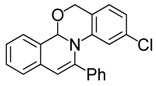	**3Ad**	81	21	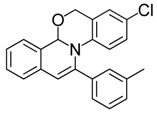	**3Ec**	85
5	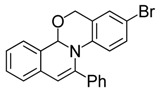	**3Ae**	77	22	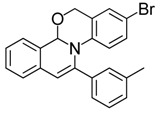	**3Ee**	68
6	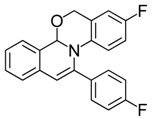	**3Bb**	85	23	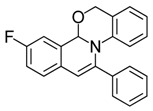	**3Fa**	90
7	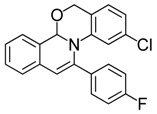	**3Bd**	82	24	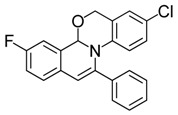	**3Fc**	70
8	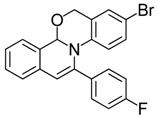	**3Be**	69	25	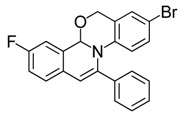	**3Fe**	85
9	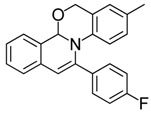	**3Bf**	68	26	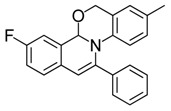	**3Ff**	45
10	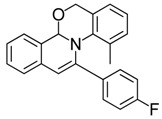	**3Bg**	65	27	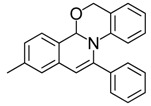	**3Ga**	90
11	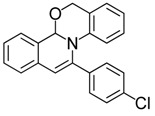	**3Ca**	88	28	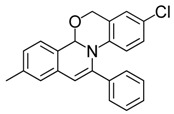	**3Gc**	77
12	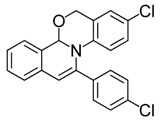	**3Cc**	82	29	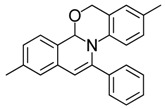	**3Gf**	85
13	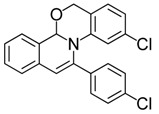	**3Cd**	80	30	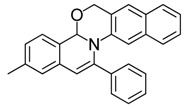	**3Gh**	55
14	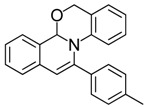	**3Da**	97	31	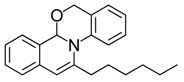	**3Ha**	82
15	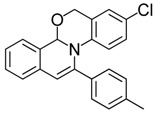	**3Dc**	76	32	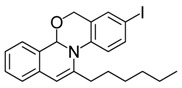	**3Hi**	80
16	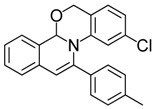	**3Dd**	80	33	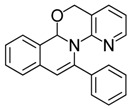	**3Aj**	47
17	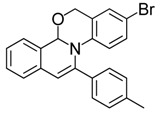	**3De**	75	34	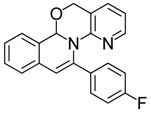	**3Bj**	45

^a^ **1A** (0.1 mmol), **2a** (0.12 mmol) and catalyst (5 mol%) in toluene (2 mL) were perfomed in a sealed vial.

## Supplementary Materials

Supplementary materials can be accessed at: http://www.mdpi.com/1420-3049/18/1/814/s1.
