# Supplementary material for: ‘One-pot’ Synthesis of Dihydrobenzo[4,5][1,3]oxazino[2,3-a]isoquinolines via a Silver(I)-Catalyzed Cascade Approach
Source: Molecules. 2013 Jan 11;18(1):814–31. doi: 10.3390/molecules18010814 (PMC6270568; doi:10.3390/molecules18010814)

Supplementary Information

Contents

The Details of the X-ray Crystallographic Structure of 3Aa………………S1

Copies of ^{1}H-NMR and ^{13}C-NMR of Compounds…………….………......S2

The Details of the X-ray Crystallographic Structure of 3Aa

Bond precision: C-C = 0.0023 A Wavelength = 0.71073

Cell:	a = 11.3144(10)	b = 5.7266(5)	c = 13.1302(11)
	alpha = 90	beta = 114.075(2)	gamma = 90
Temperature:	293 K		

	Calculated	Reported
Volume	776.74(12)	776.74(12)
Space group	P 21	P2(1)
Hall group	P 2yb	P 2yb
Moiety formula	C22 H17 N O	C22 H17 N O
Sum formula	C22 H17 N O	C22 H17 N O
Mr	311.37	311.37
Dx,g cm-3	1.331	1.331
Z	2	2
Mu (mm-1)	0.081	0.081
F000	328.0	328.0
F000'	328.13	
h,k,lmax	13,7,16	13,7,16
Nref	1682[3040]	2955
Tmin,Tmax	0.980,0.988	0.835,1.000
Tmin'	0.975	

Correction method = EMPIRICAL

Data completeness = 1.76/0.97 Theta(max) = 26.000

R(reflections) = 0.0315(2812) wR2(reflections) = 0.0841(2955)

S = 1.056 Npar = 218

CCDC 899938 contains the supplementary crystallographic data for this paper. These data can be also obtained free of charge from The Cambridge Crystallographic Data Centre via www.ccdc.cam.ac.uk/data_request/cif.

Copies of ^{1}H-NMR and ^{13}C-NMR of Compounds

12-Phenyl-4*b*,6-dihydrobenzo[4,5][1,3] oxazino[2,3-*a*]isoquinoline (3Aa)

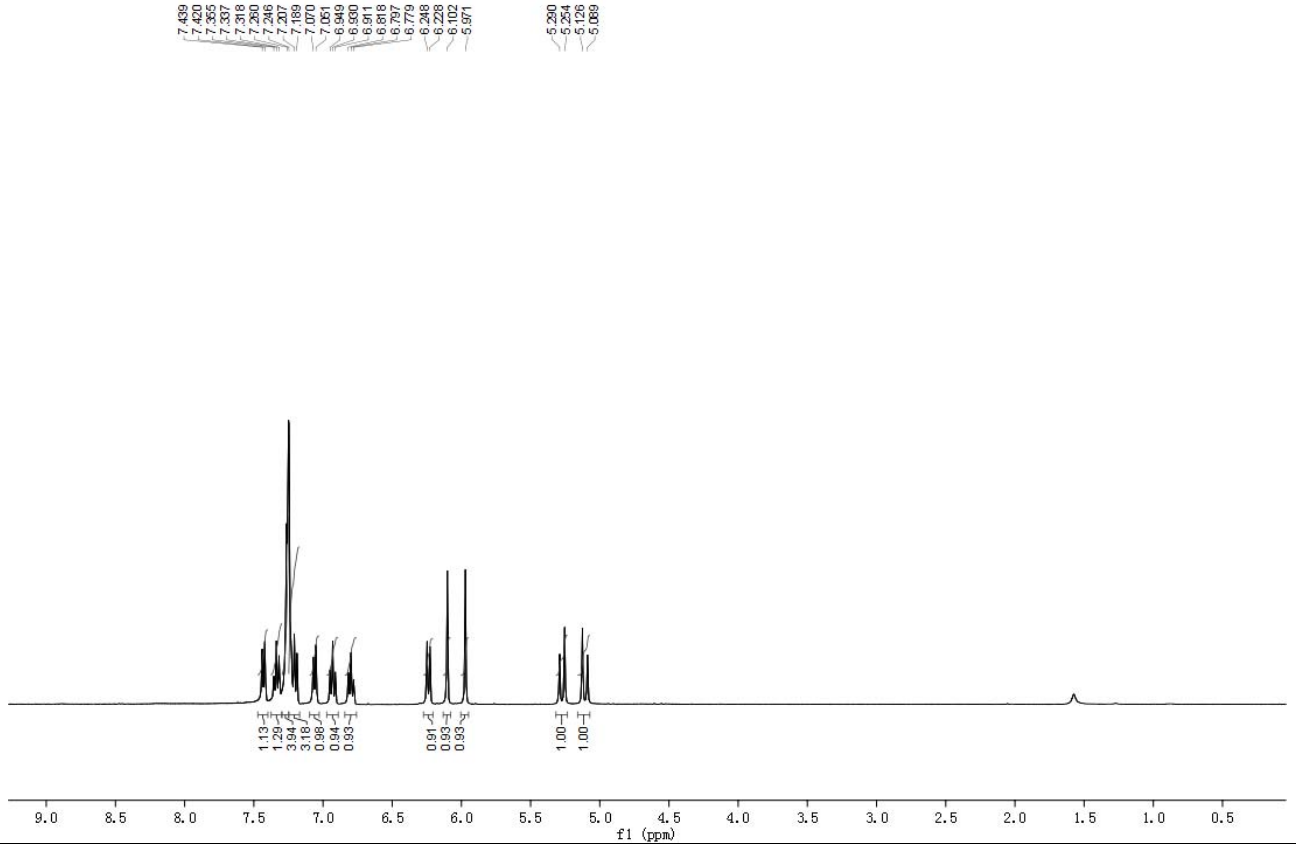

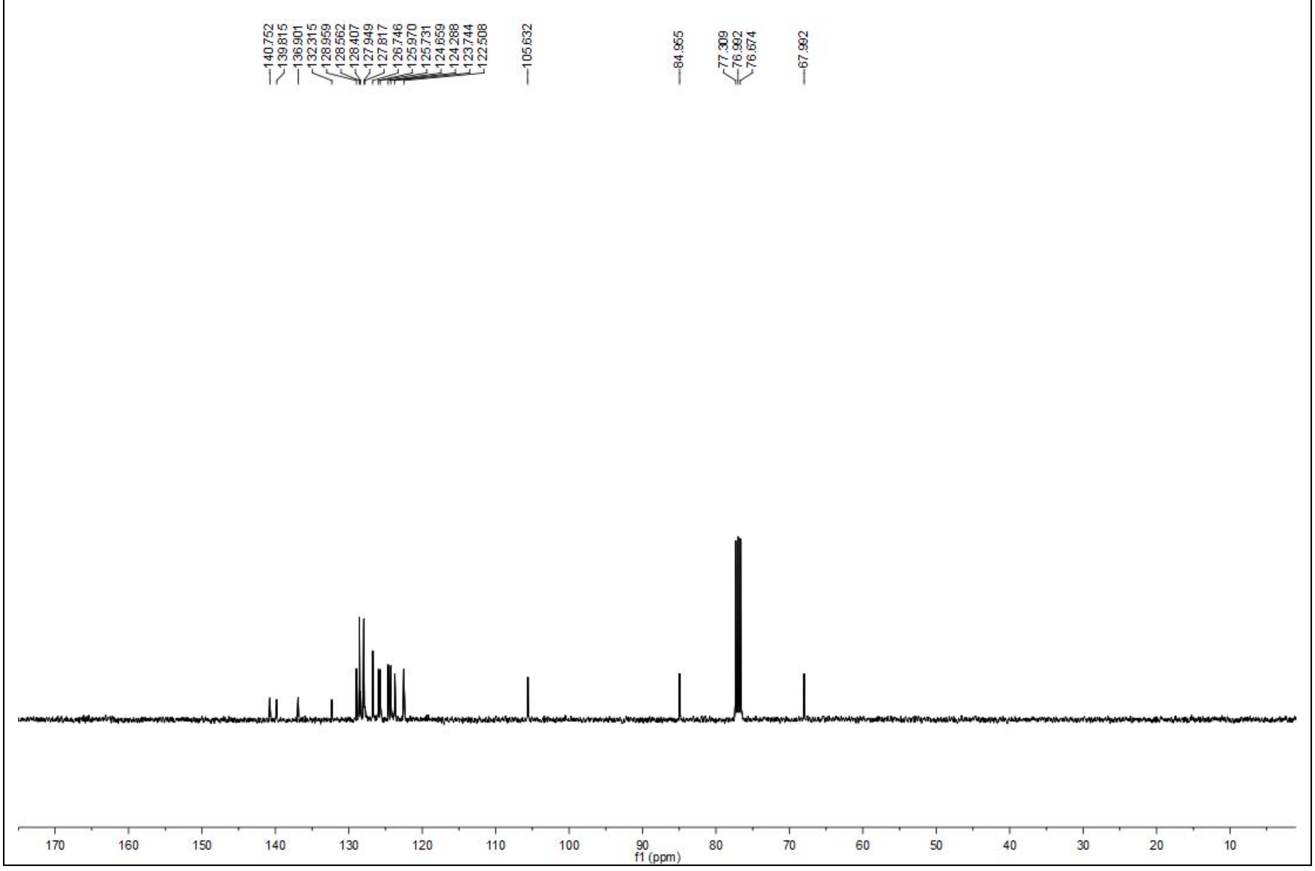

8-Fluoro-12-phenyl-4b,6-dihydrobenzo[4,5][1,3]oxazino[2,3-*a*]isoquinoline (3Ab)

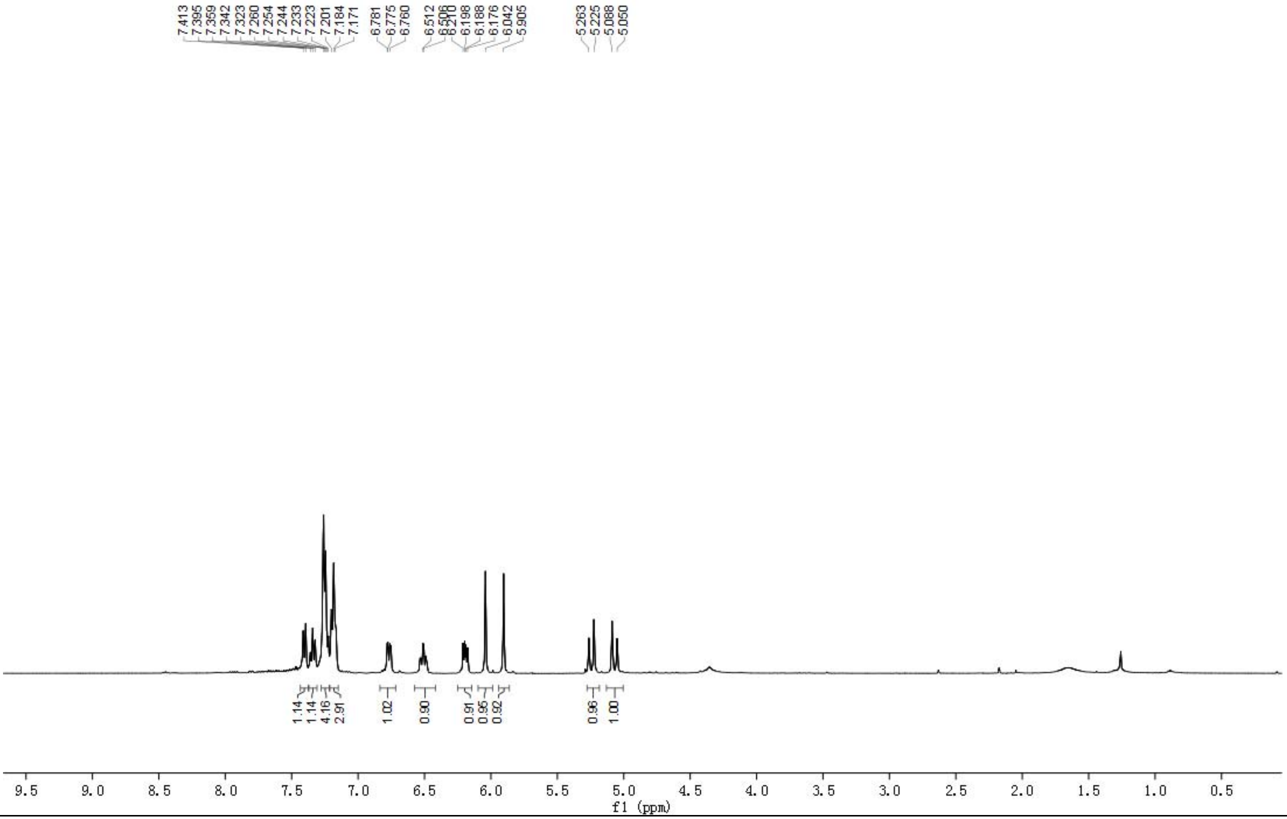

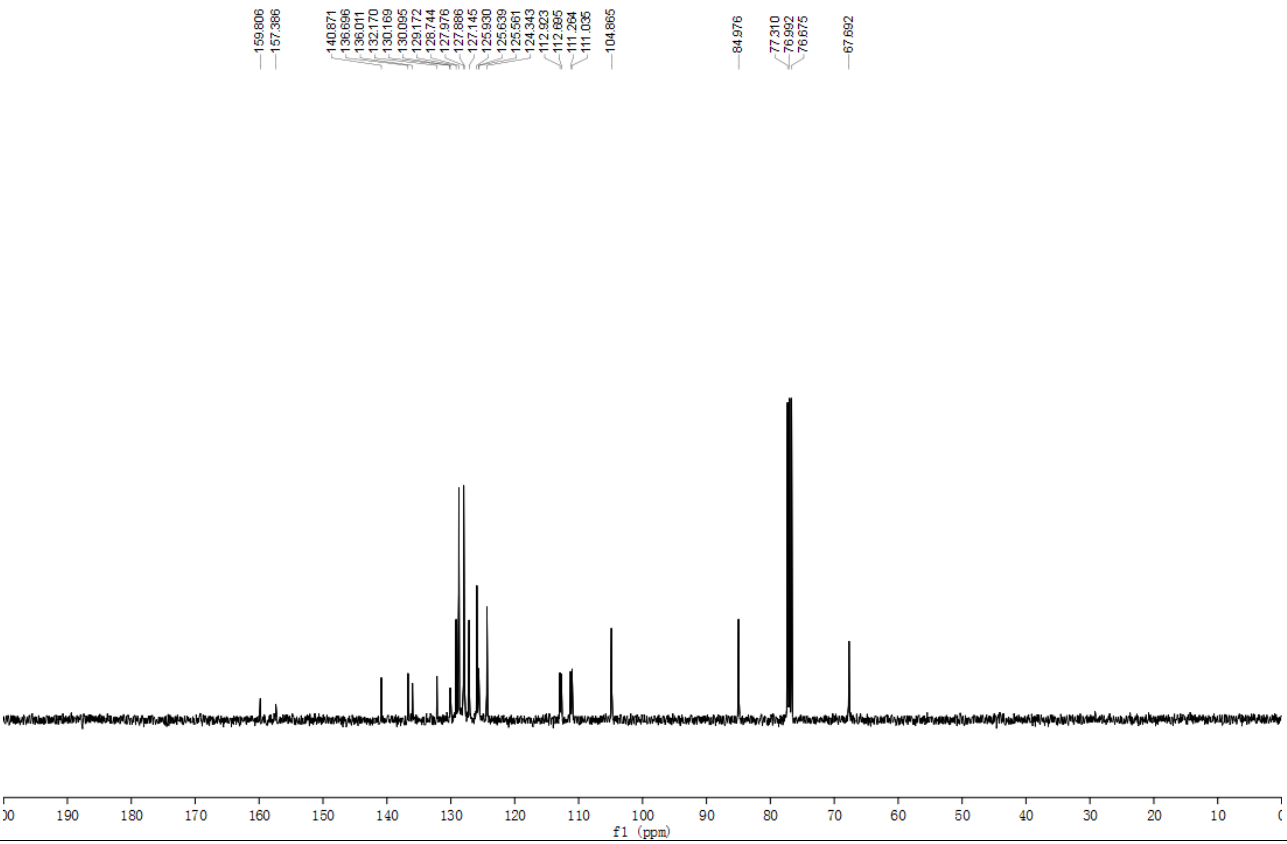

8-Chloro-12-phenyl-4b,6-dihydrobenzo[4,5][1,3]oxazino[2,3-*a*]isoquinoline (3Ac)

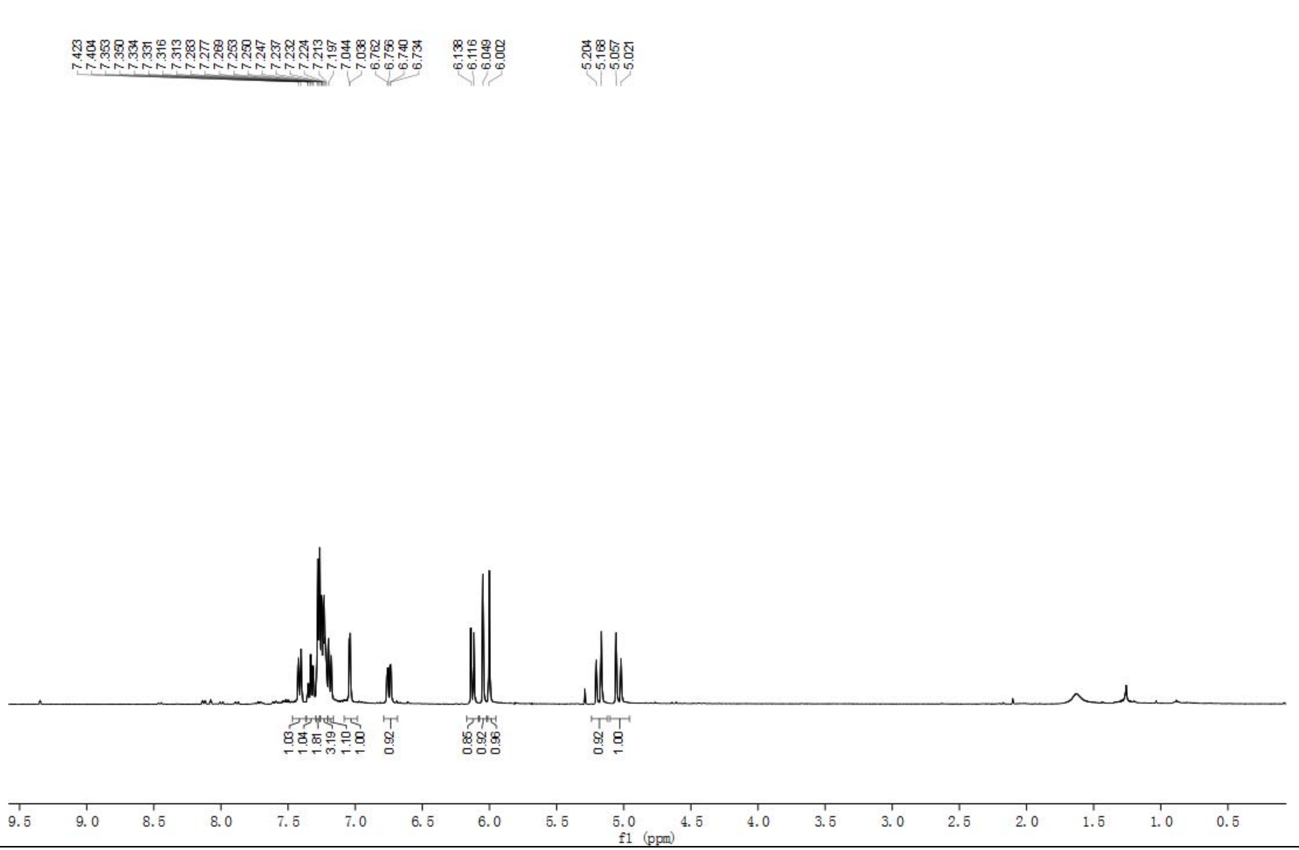

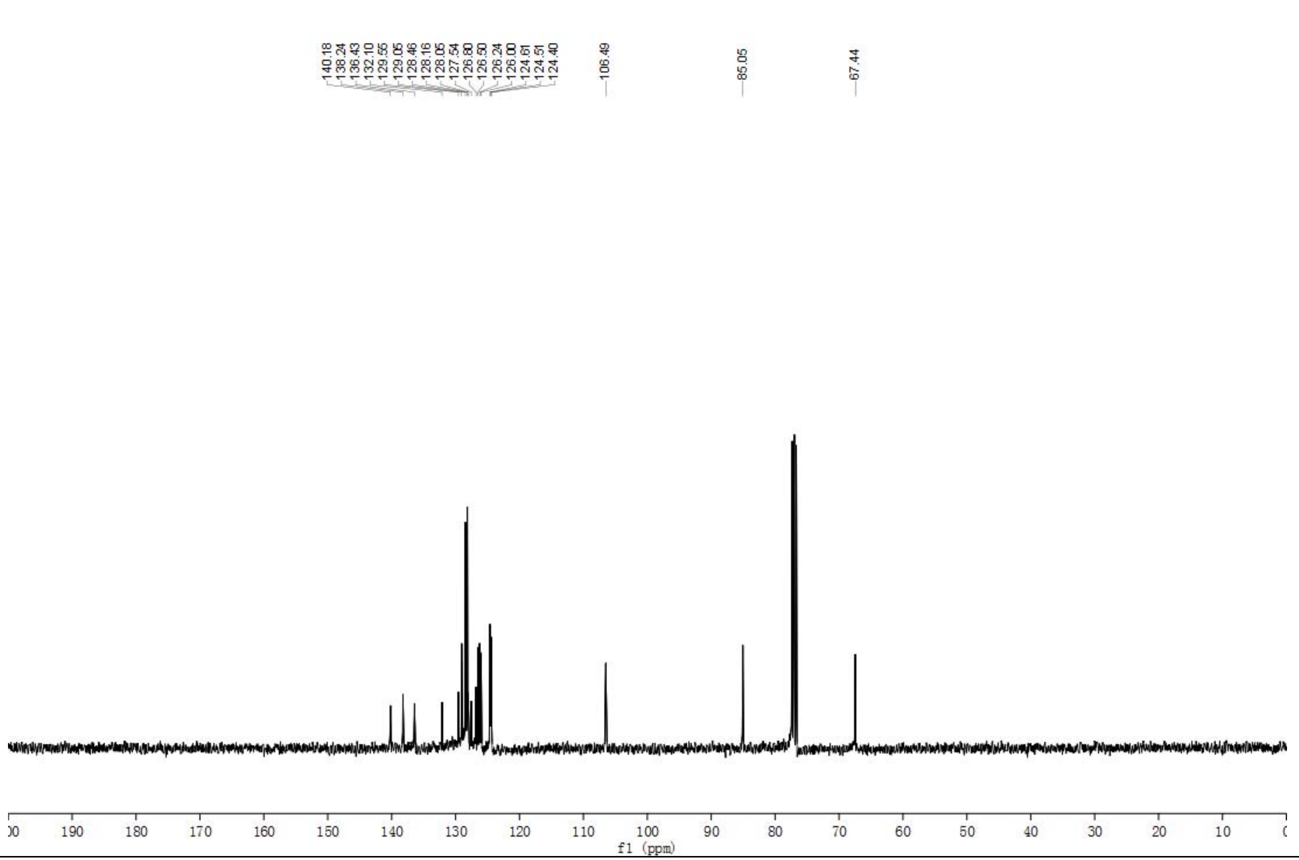

9-Chloro-12-phenyl-4b,6-dihydrobenzo[4,5][1,3]oxazino[2,3-*a*]isoquinoline (3Ad)

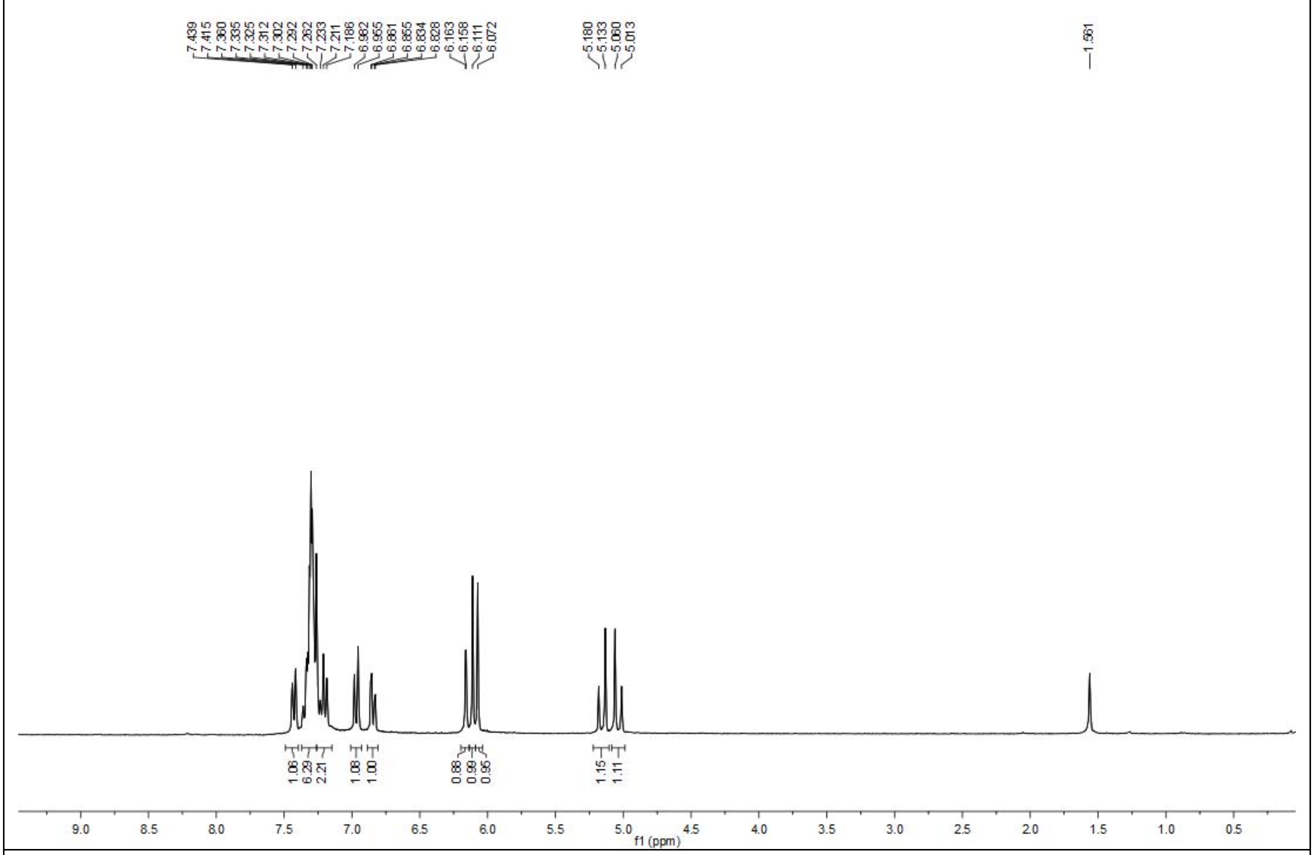

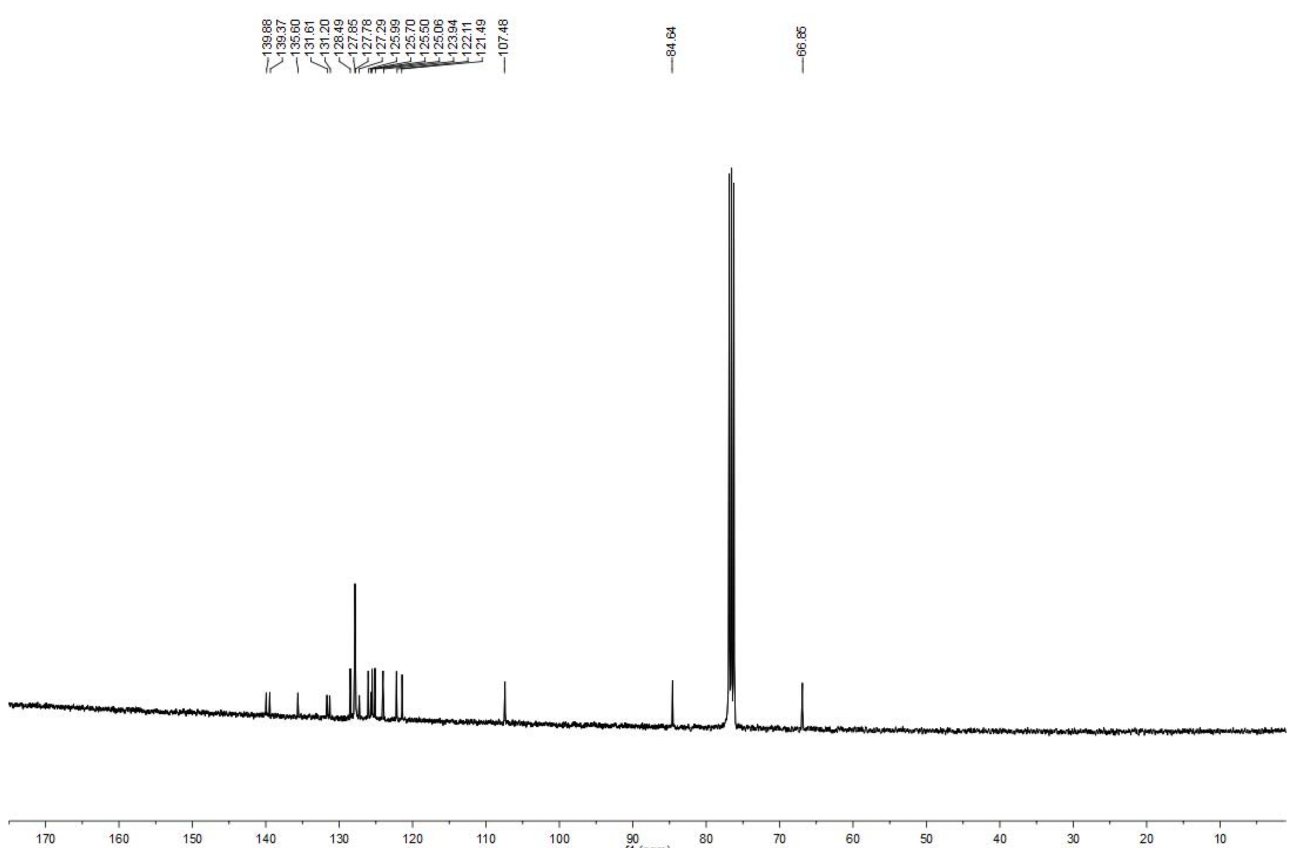

8-Bromo-12-phenyl-4b,6-dihydrobenzo[4,5][1,3]oxazino[2,3-*a*]isoquinoline (3Ae)

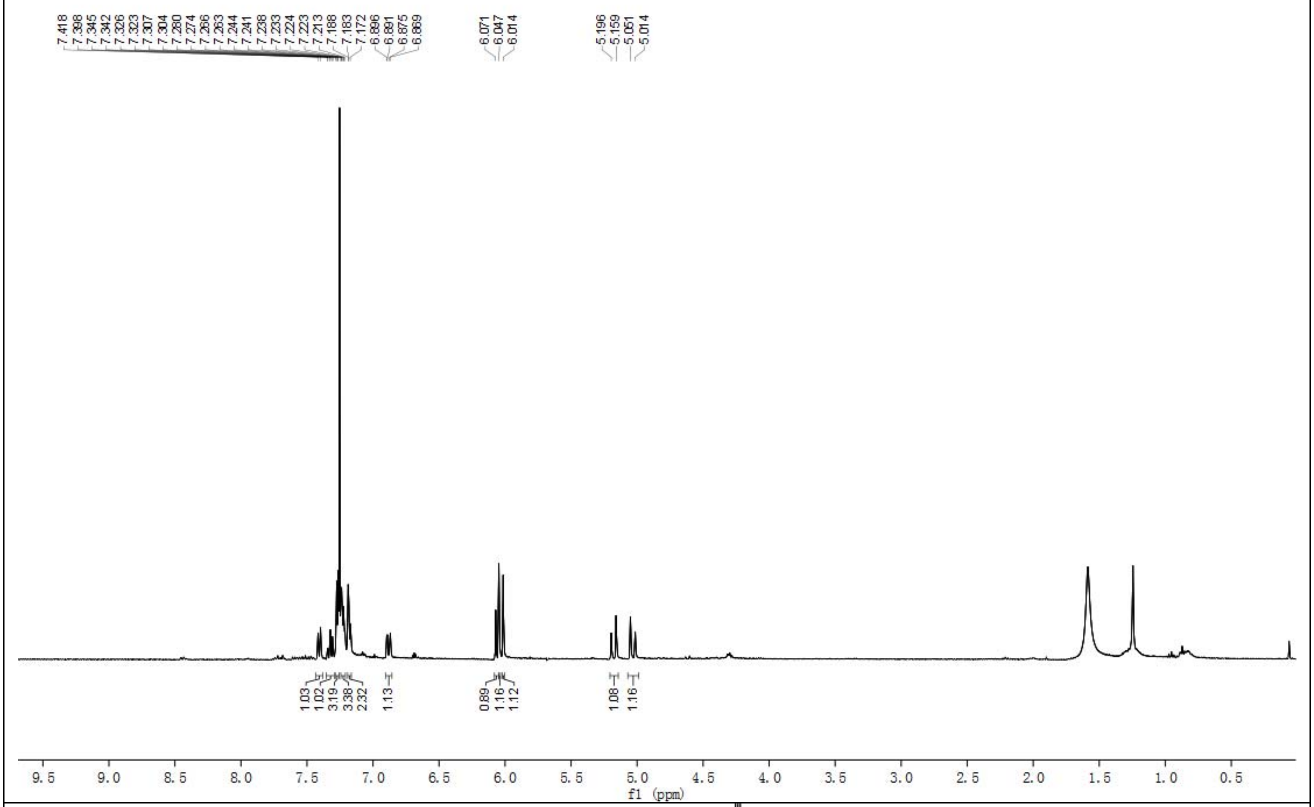

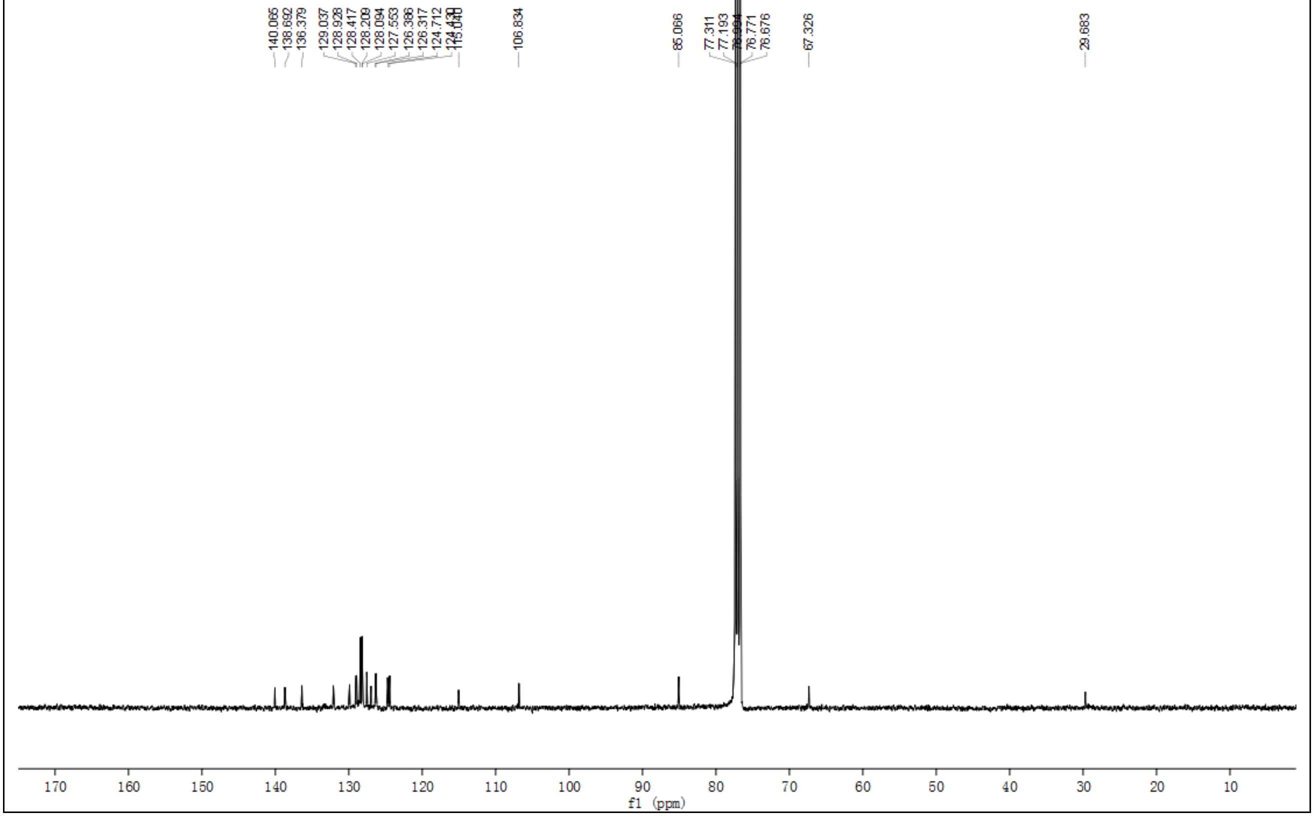

8-Fluoro-12-(4-fluorophenyl)-4b,6-dihydrobenzo[4,5][1,3]oxazino[2,3-*a*]isoquinoline (3Bb)

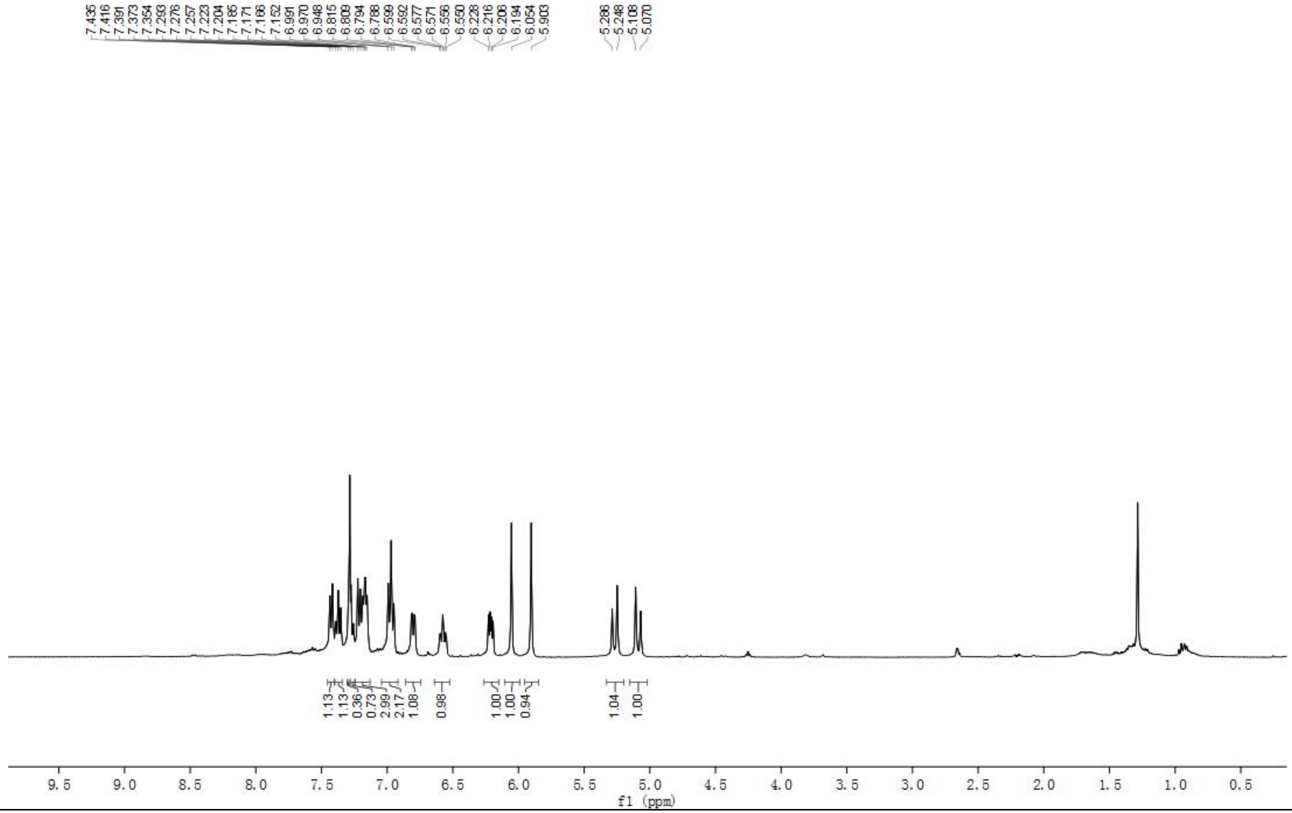

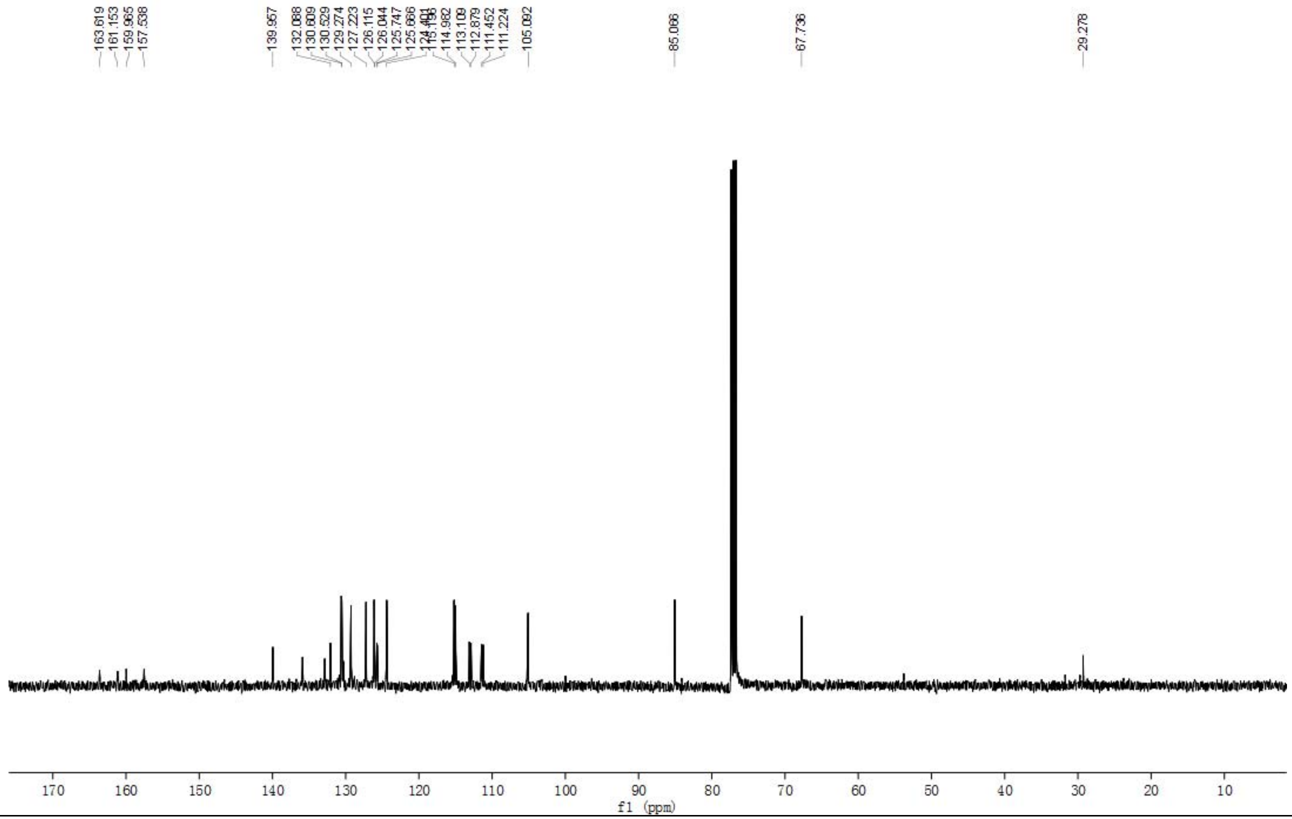

9-Chloro-12-(4-fluorophenyl)-4b,6-dihydrobenzo[4,5][1,3]oxazino[2,3-*a*]isoquinoline (3Bd)

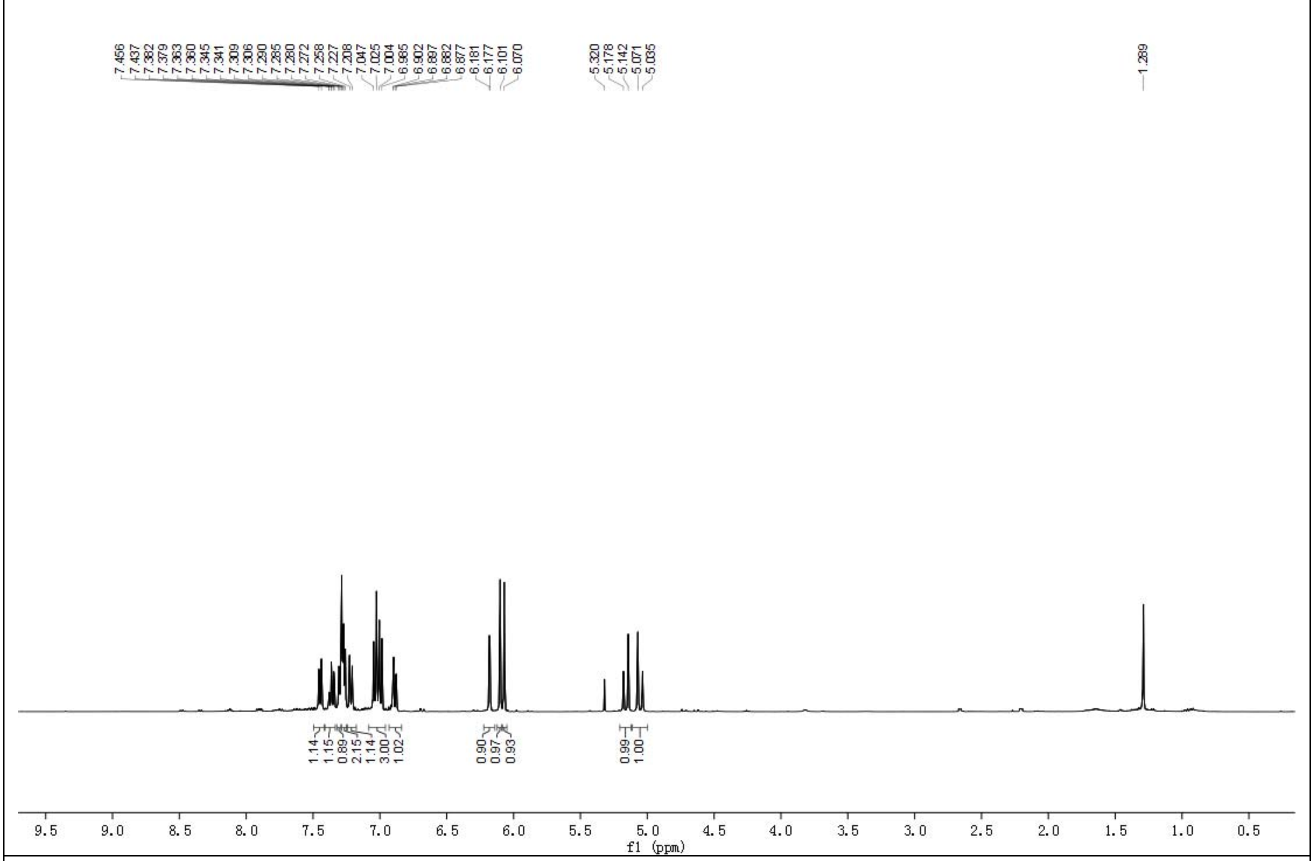

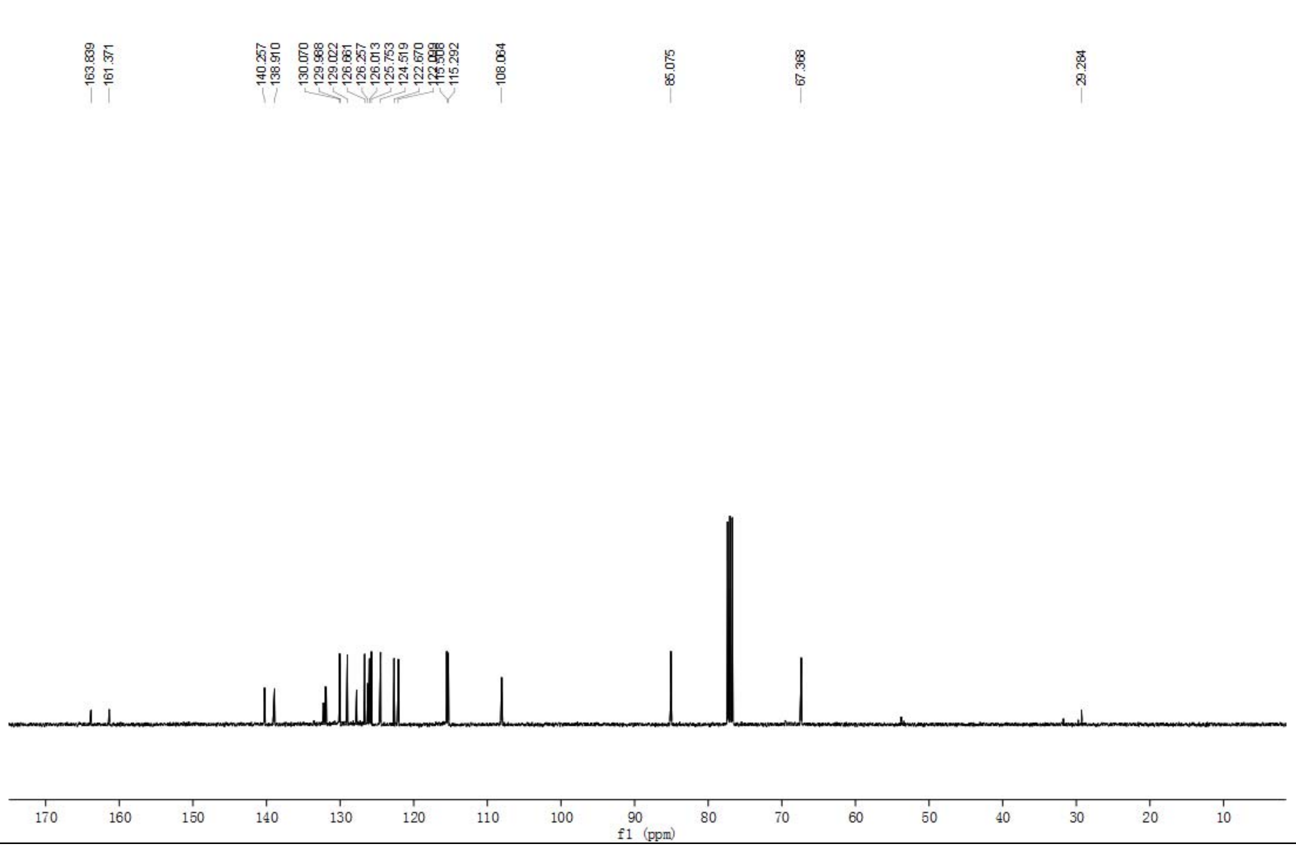

8-Bromo-12-(4-fluorophenyl)-4b,6-dihydrobenzo[4,5][1,3]oxazino[2,3-*a*]isoquinoline (3Be)

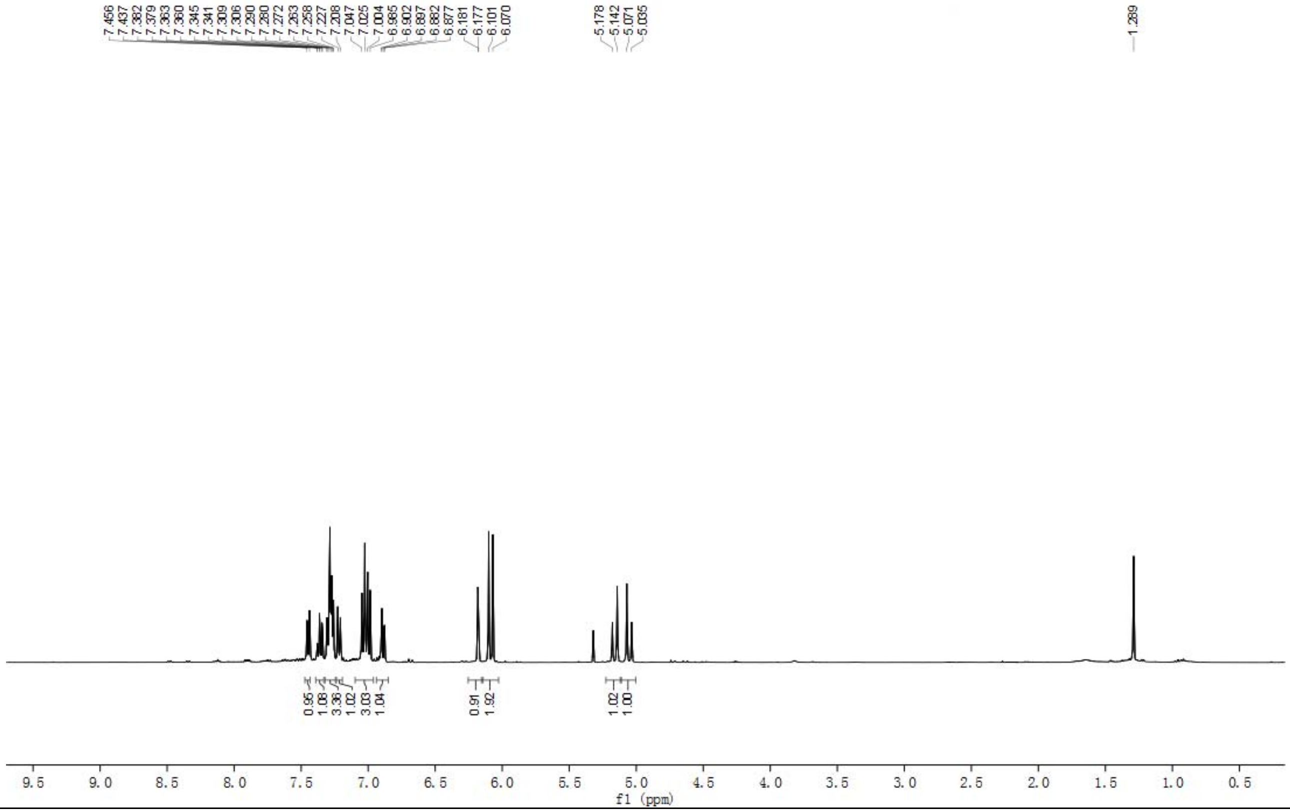

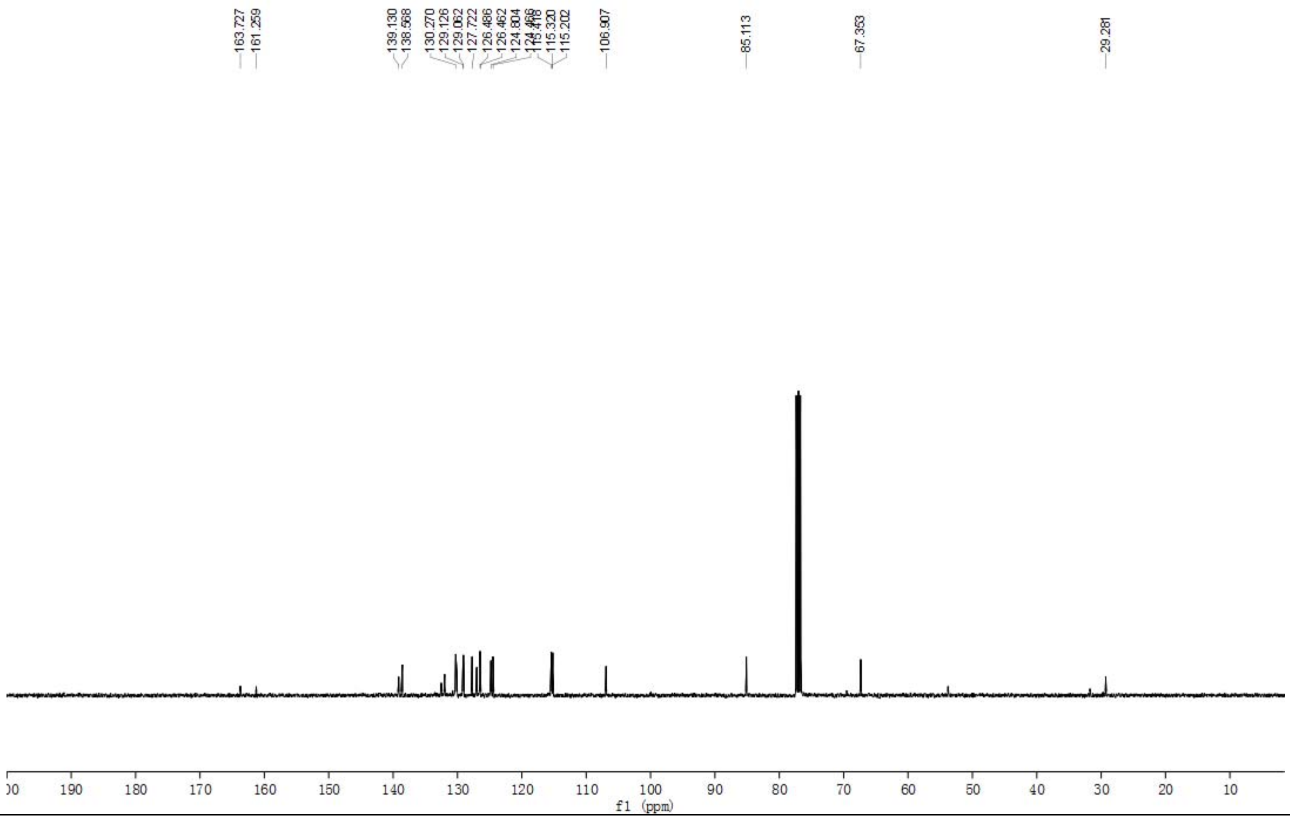

12-(4-Fluorophenyl)-8-methyl-4b,6-dihydrobenzo[4,5][1,3]oxazino[2,3-*a*]isoquinoline (3Bf)

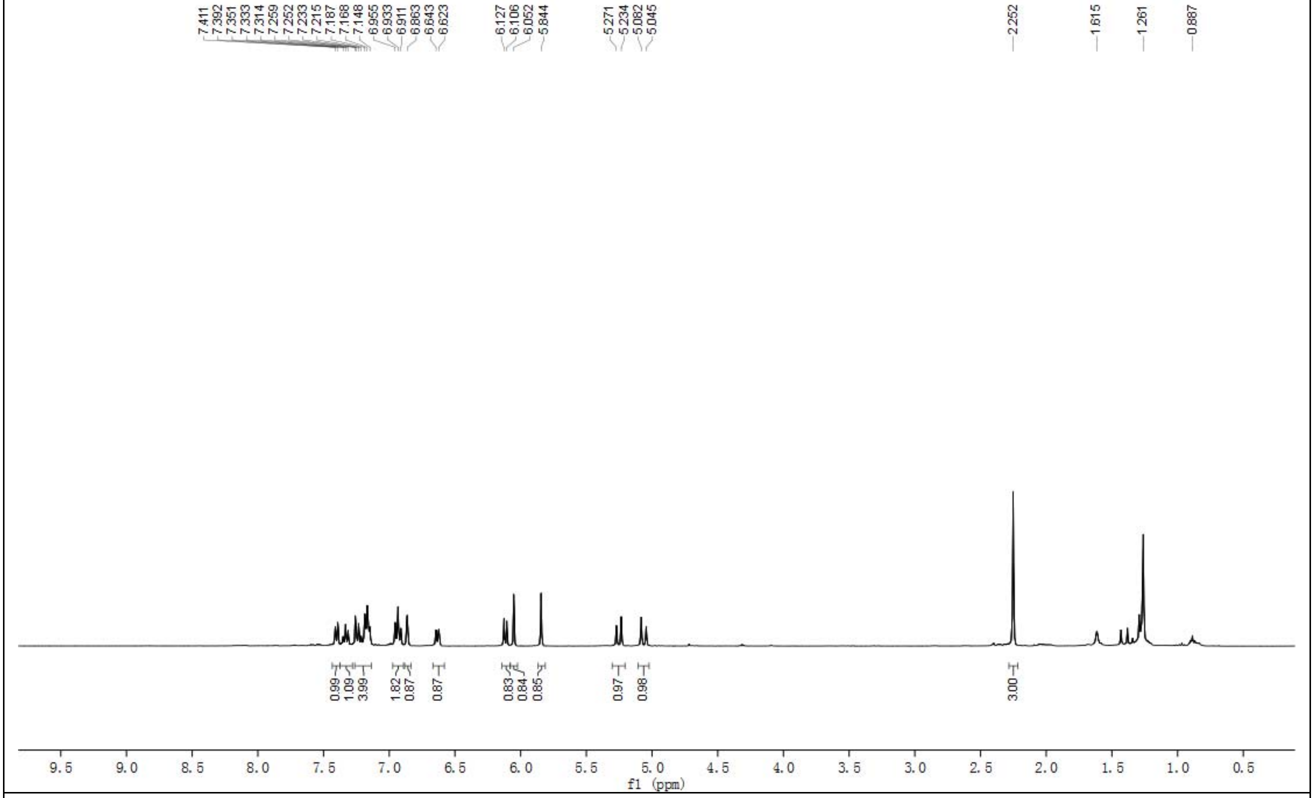

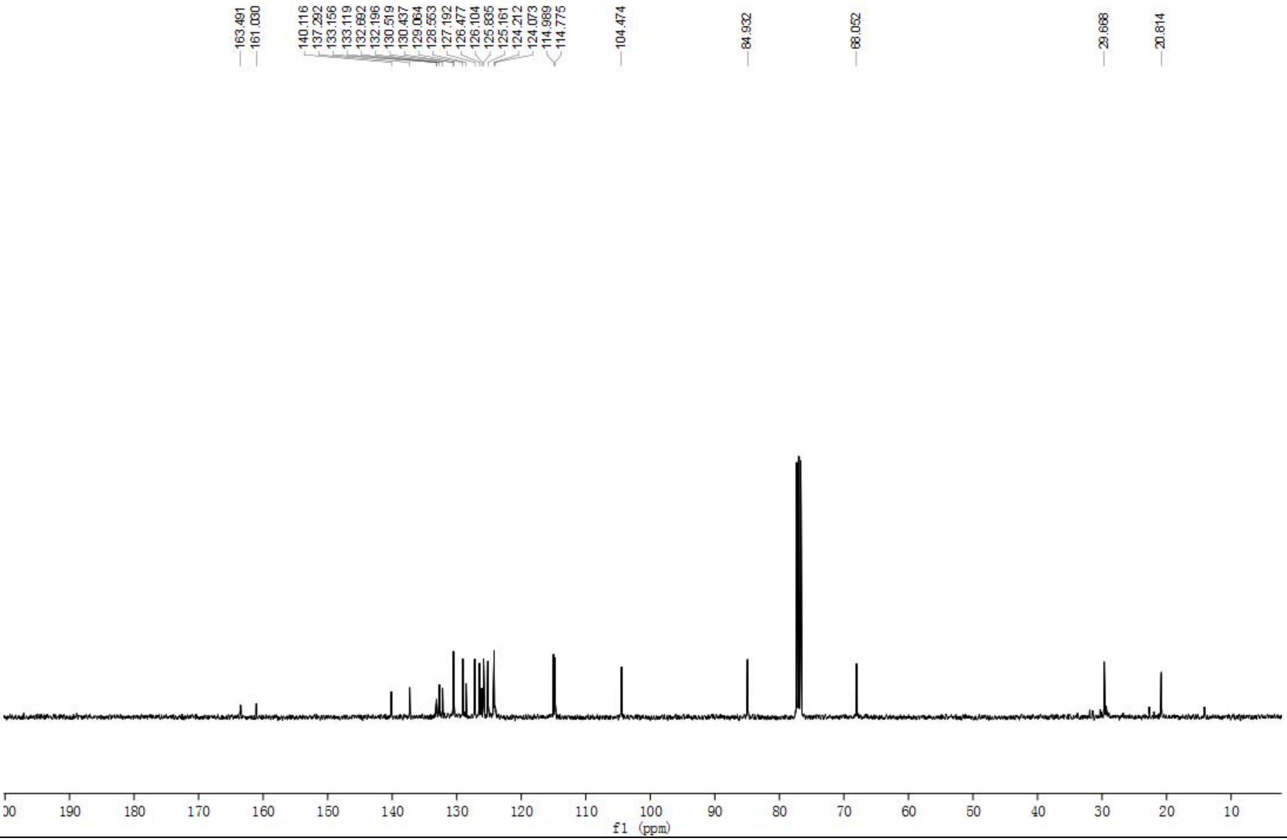

12-(4-Fluorophenyl)-10-methyl-4b,6-dihydrobenzo[4,5][1,3]oxazino[2,3-*a*]isoquinoline (3Bg)

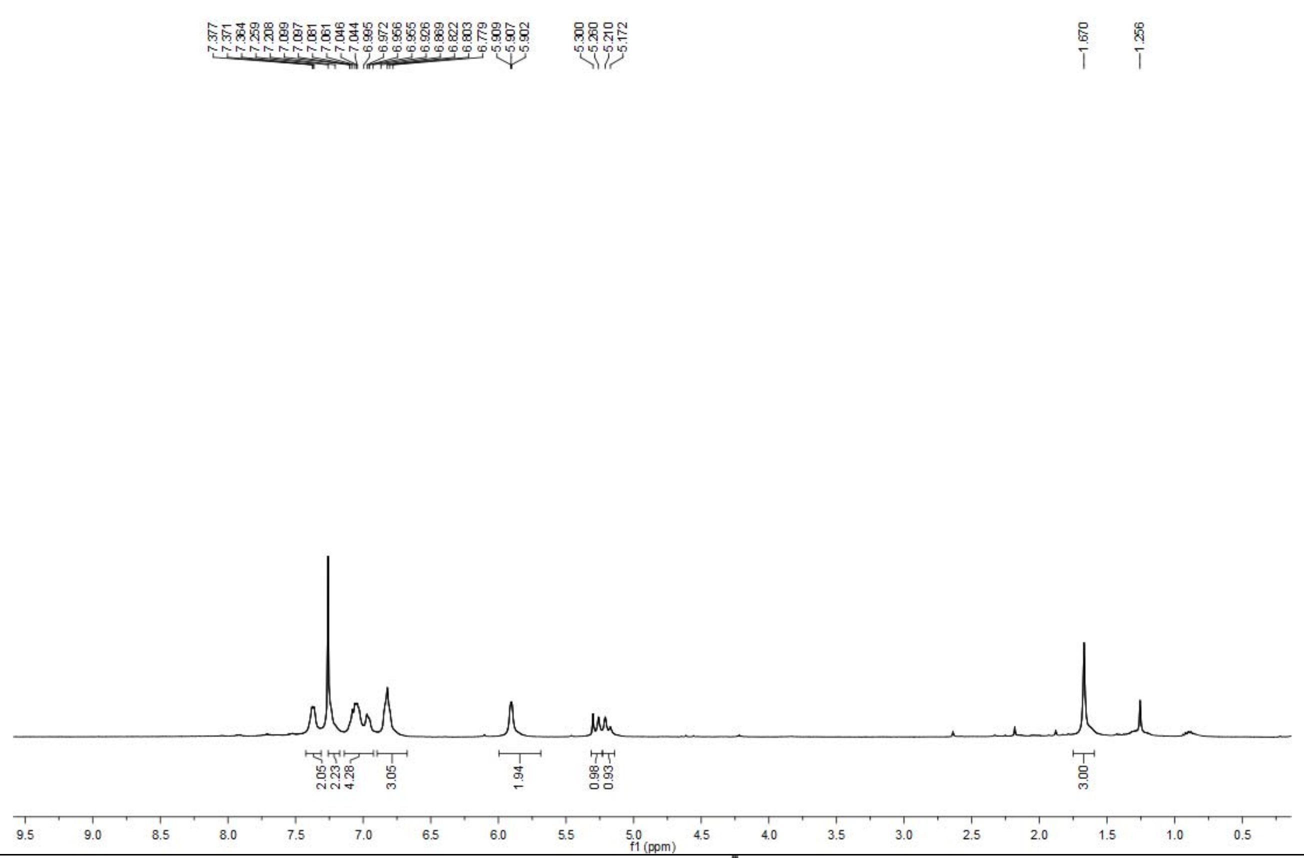

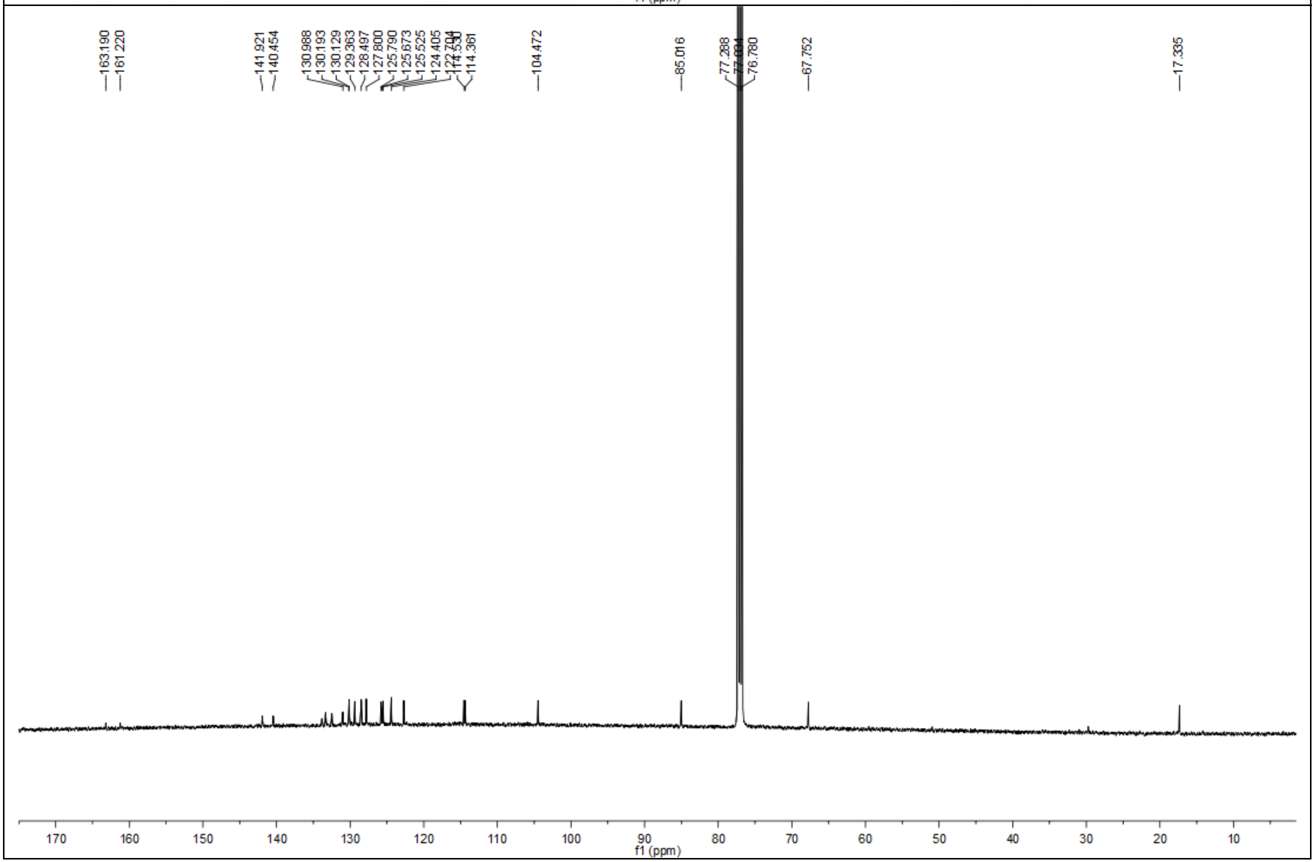

12-(4-Chlorophenyl)-4b,6-dihydrobenzo[4,5][1,3]oxazino[2,3-*a*]isoquinoline (3Ca)

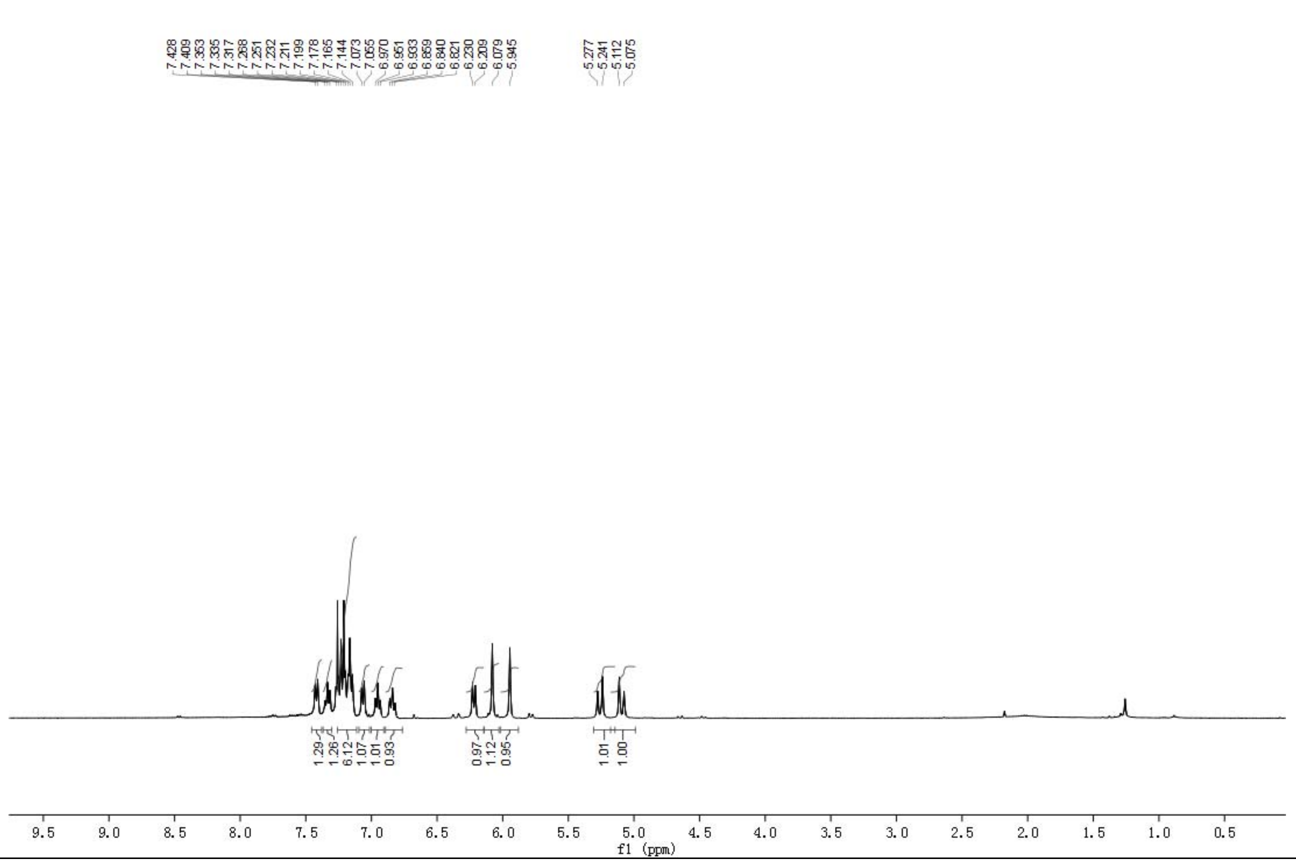

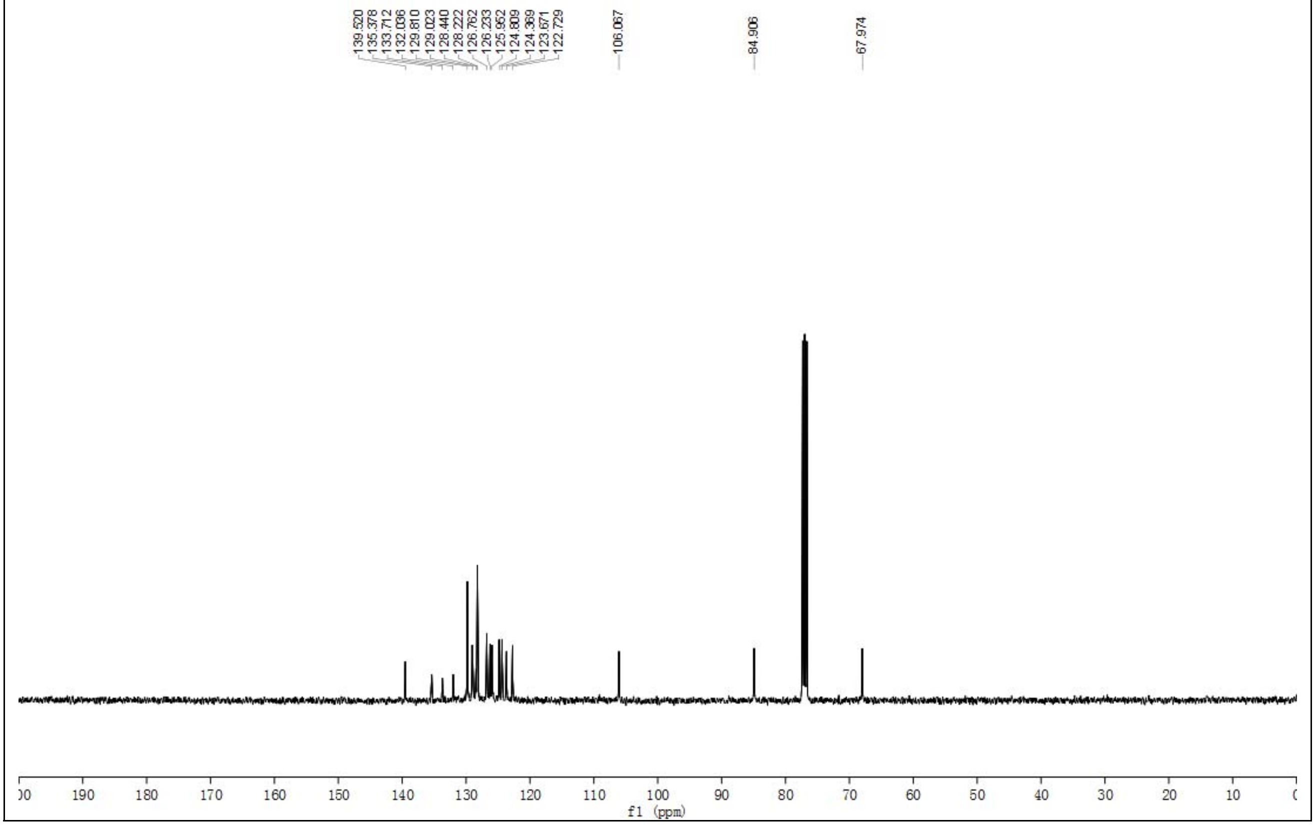

8-cChloro-12-(4-chlorophenyl)-4b,6-dihydrobenzo[4,5][1,3]oxazino[2,3-*a*]isoquinoline (3Cc)

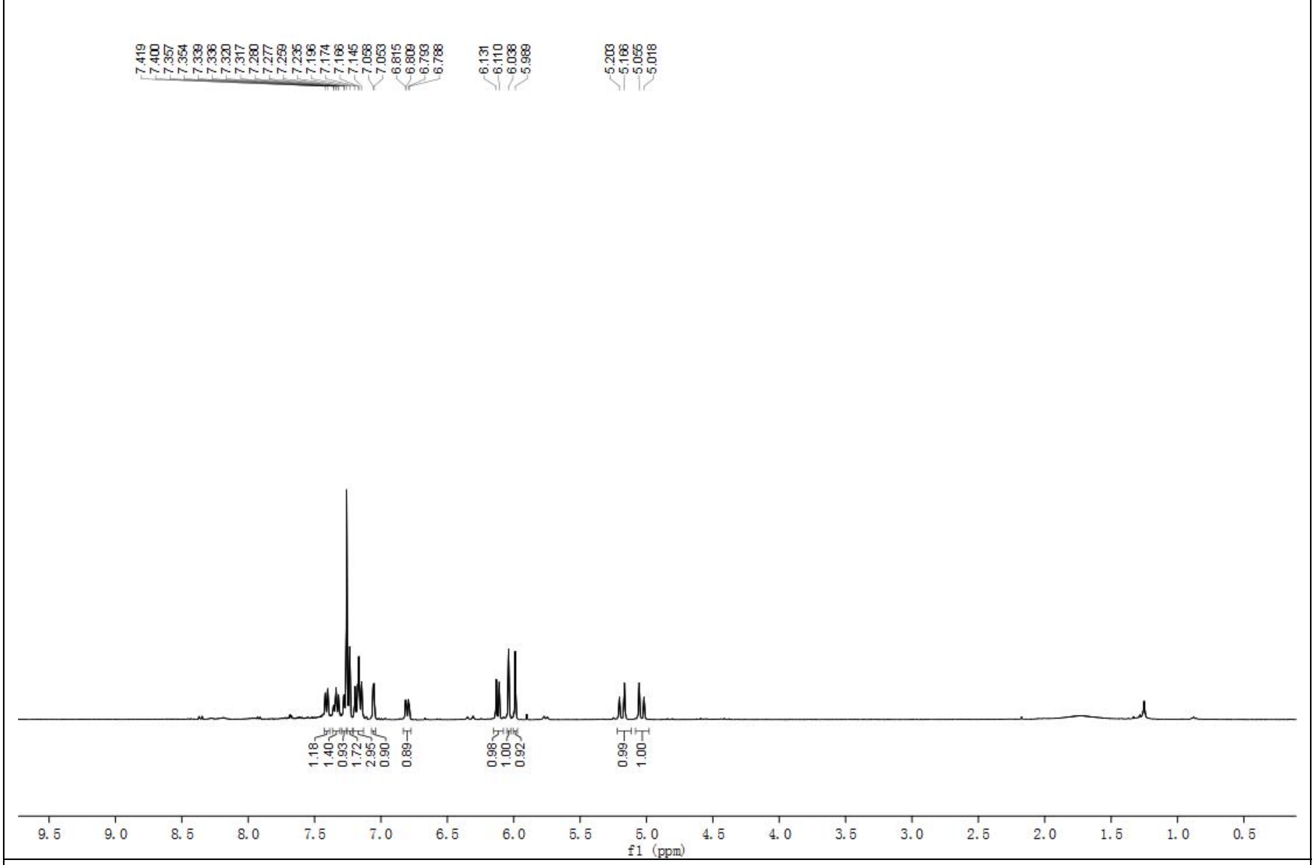

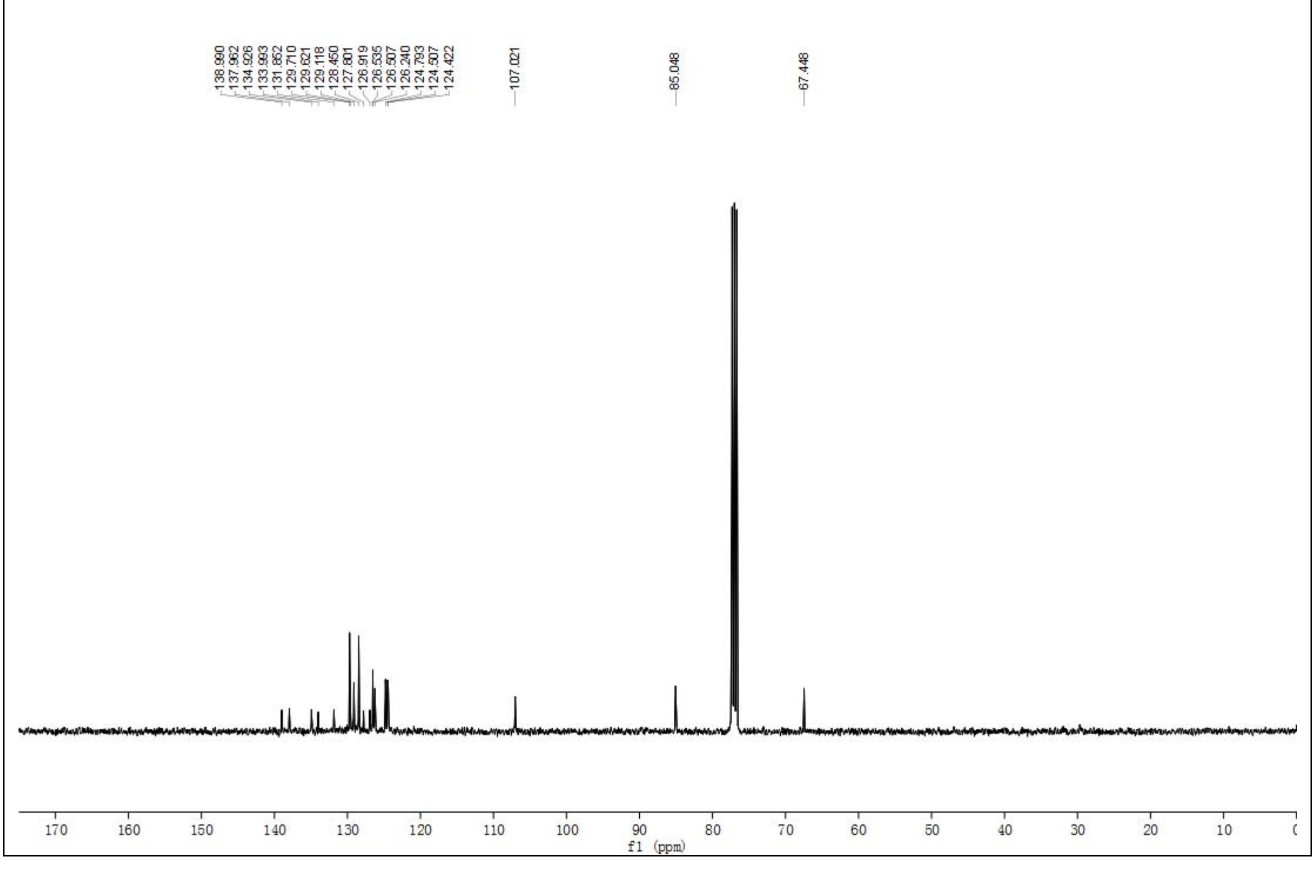

9-Chloro-12-(4-chlorophenyl)-4b,6-dihydrobenzo[4,5][1,3]oxazino[2,3-*a*]isoquinoline (3Cd)

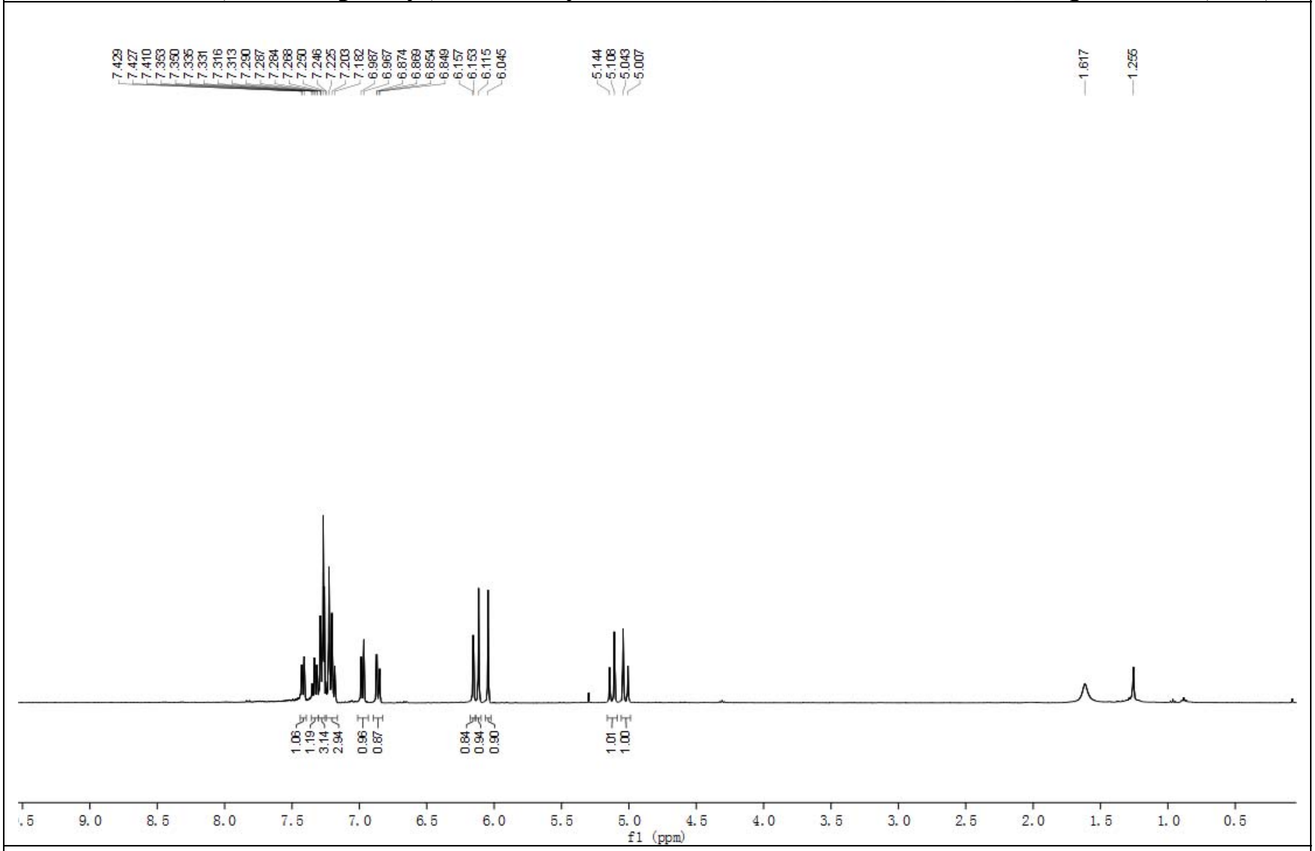

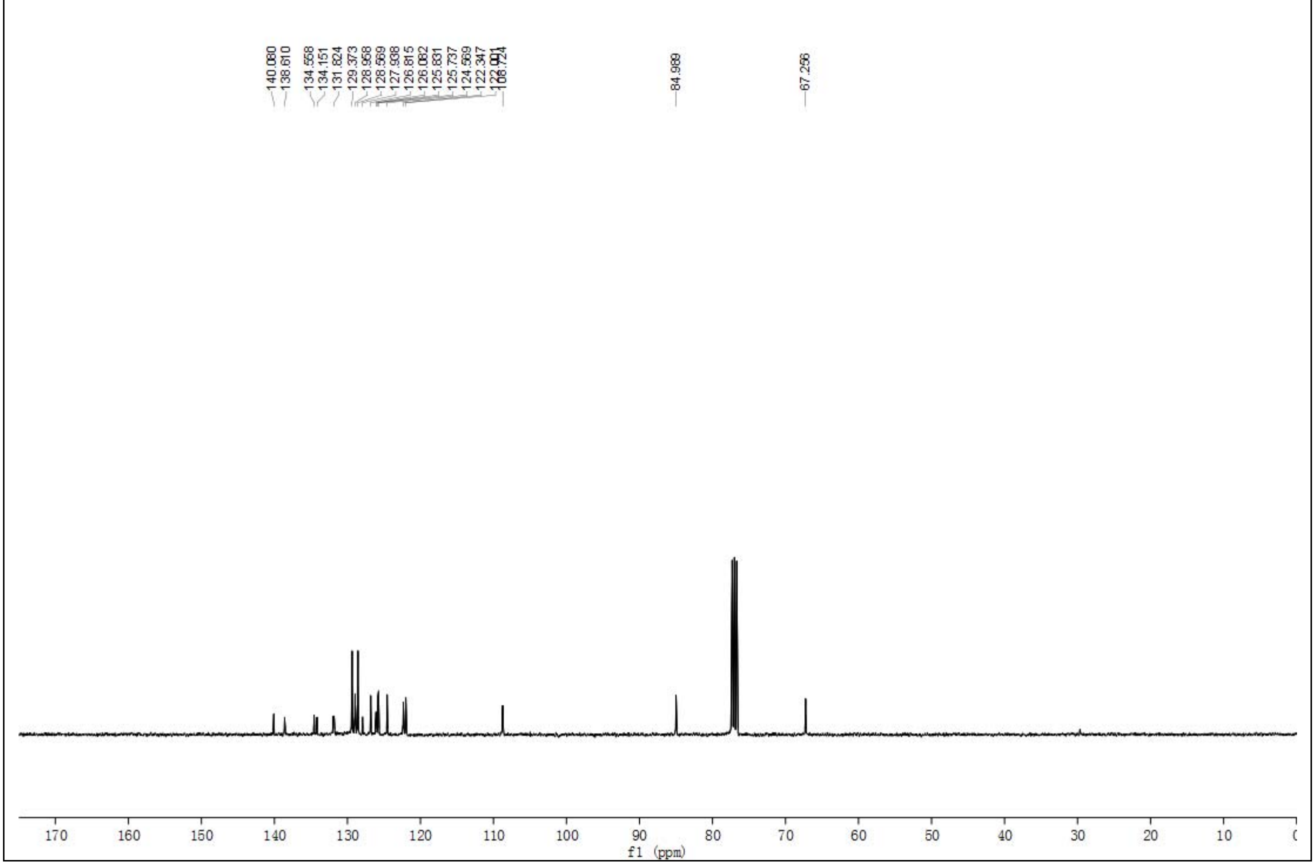

12-(*p*-Tolyl)-4b,6-dihydrobenzo[4,5][1,3]oxazino[2,3-*a*]isoquinoline (3Da)

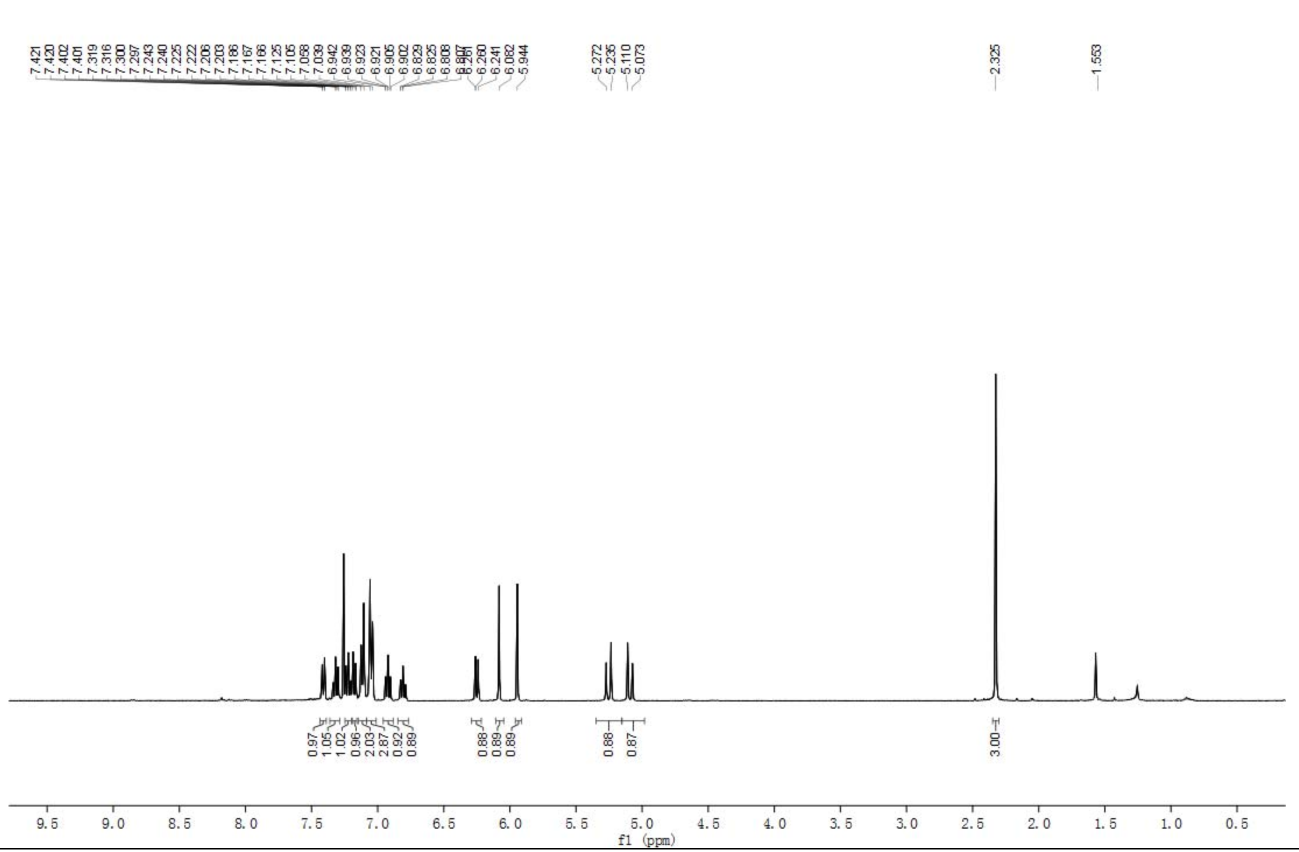

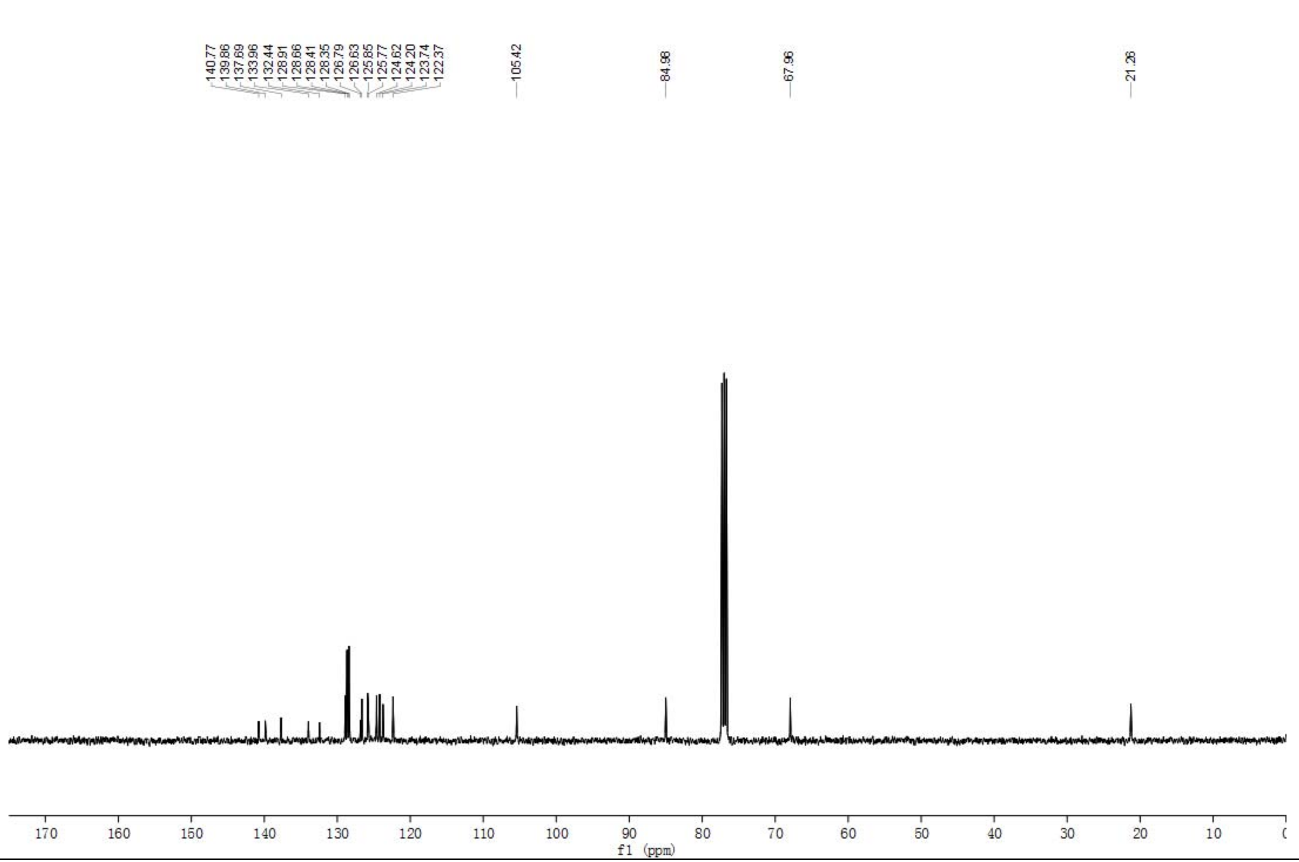

8-Chloro-12-(p-tolyl)-4b,6-dihydrobenzo[4,5][1,3]oxazino[2,3-*a*]isoquinoline (3Dc)

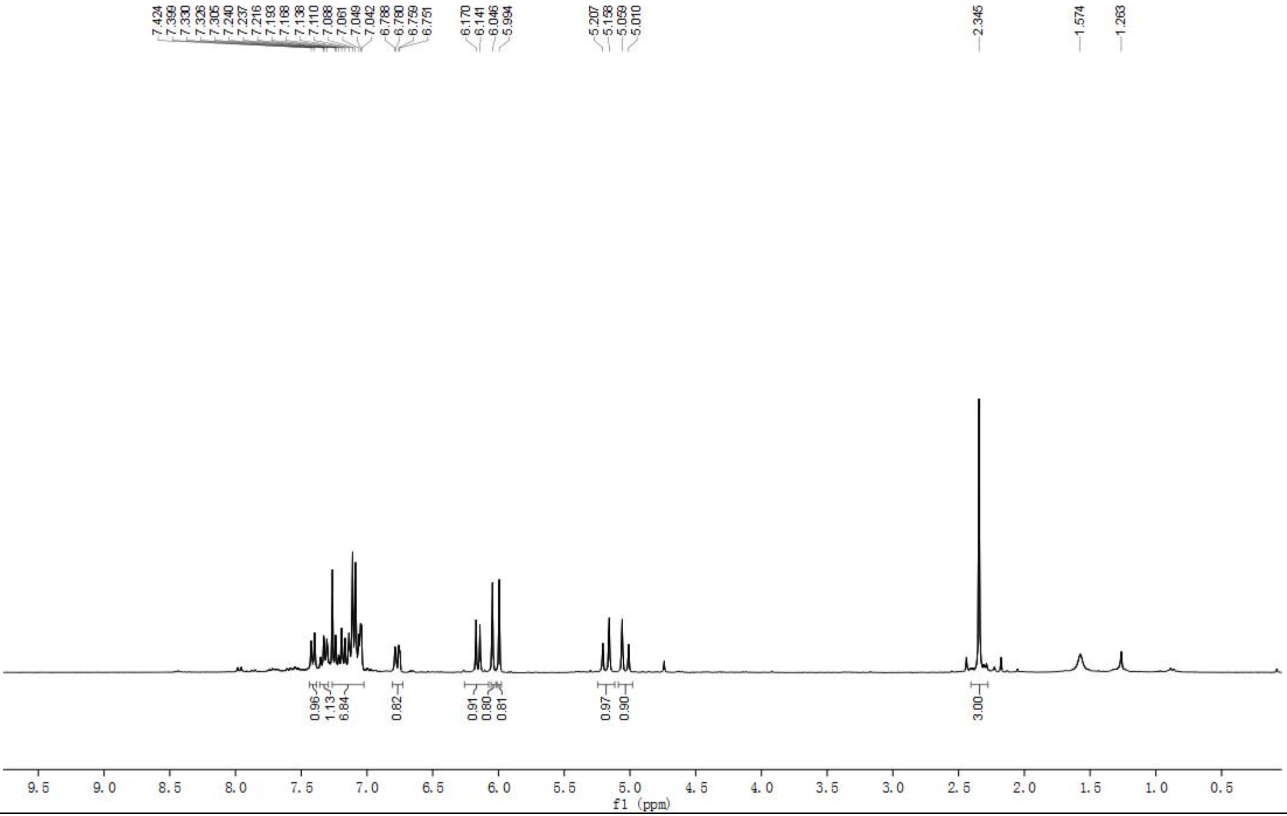

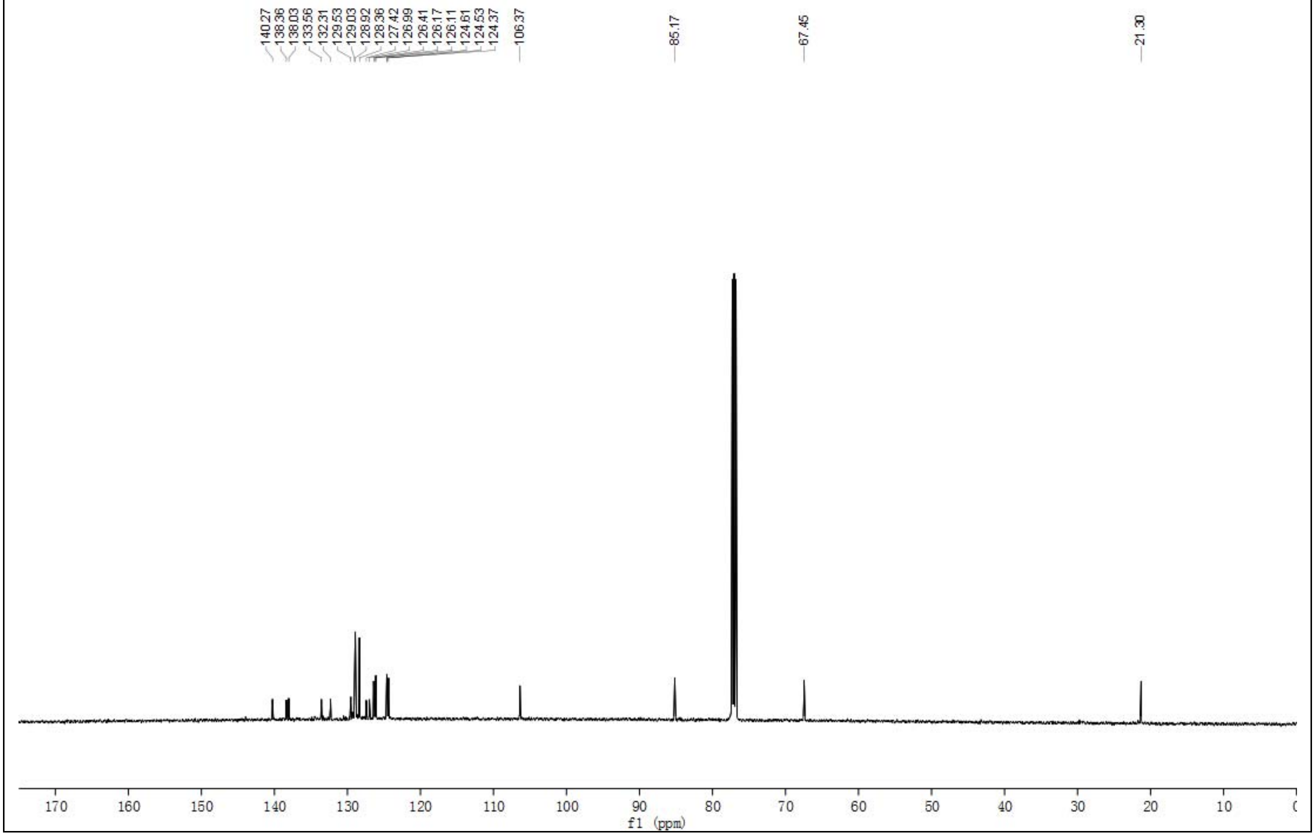

9-Chloro-12-(p-tolyl)-4b,6-dihydrobenzo[4,5][1,3]oxazino[2,3-*a*]isoquinoline (3Dd)

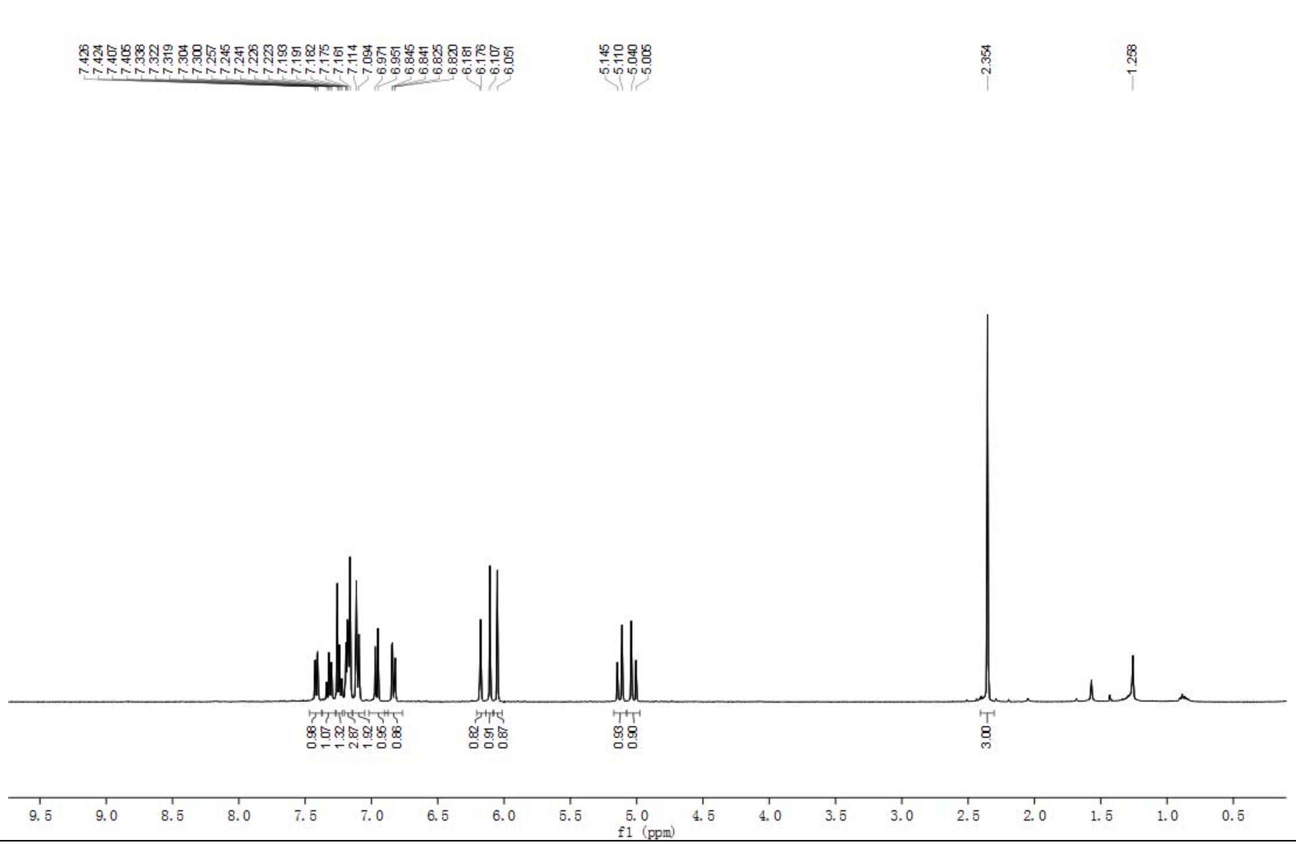

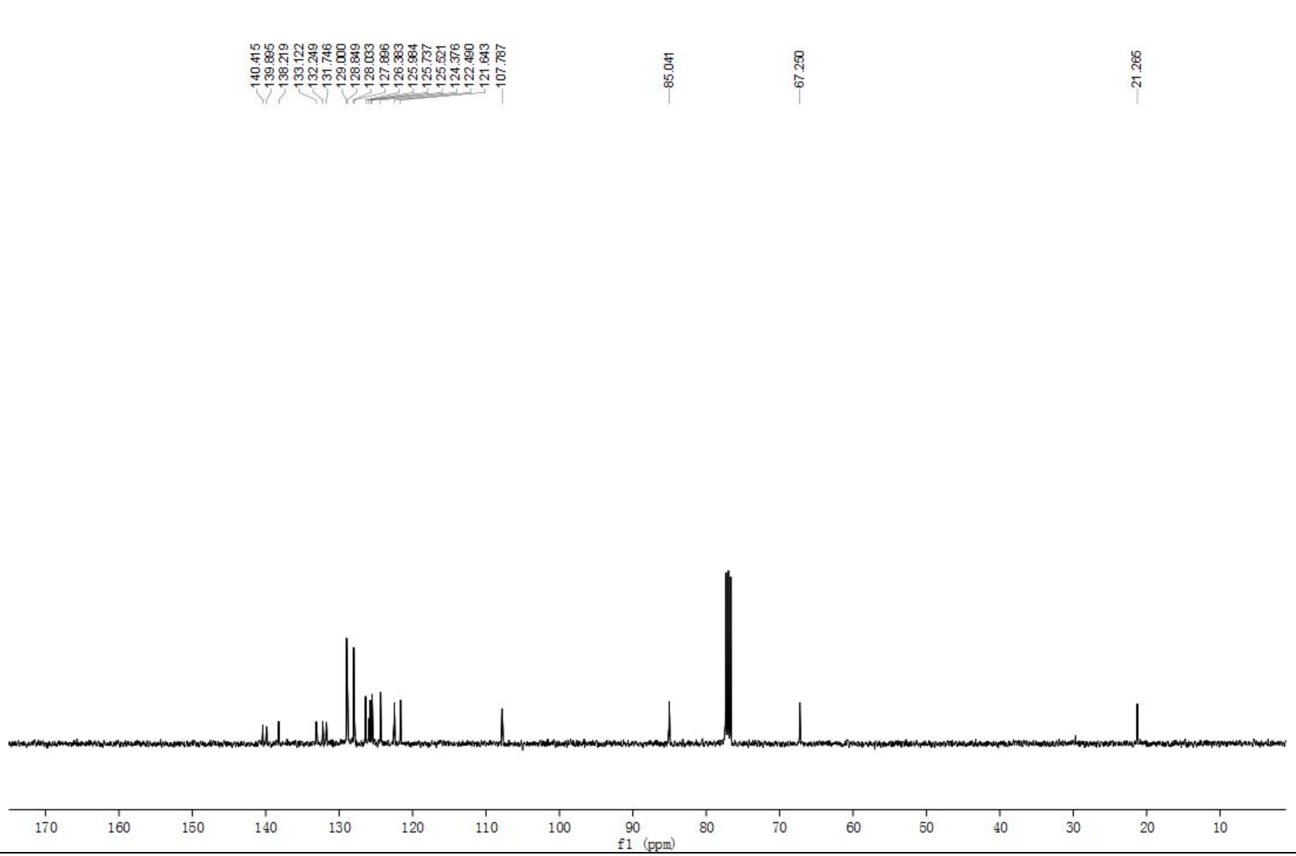

8-Bromo-12-(p-tolyl)-4b,6-dihydrobenzo[4,5][1,3]oxazino[2,3-*a*]isoquinoline (3De)

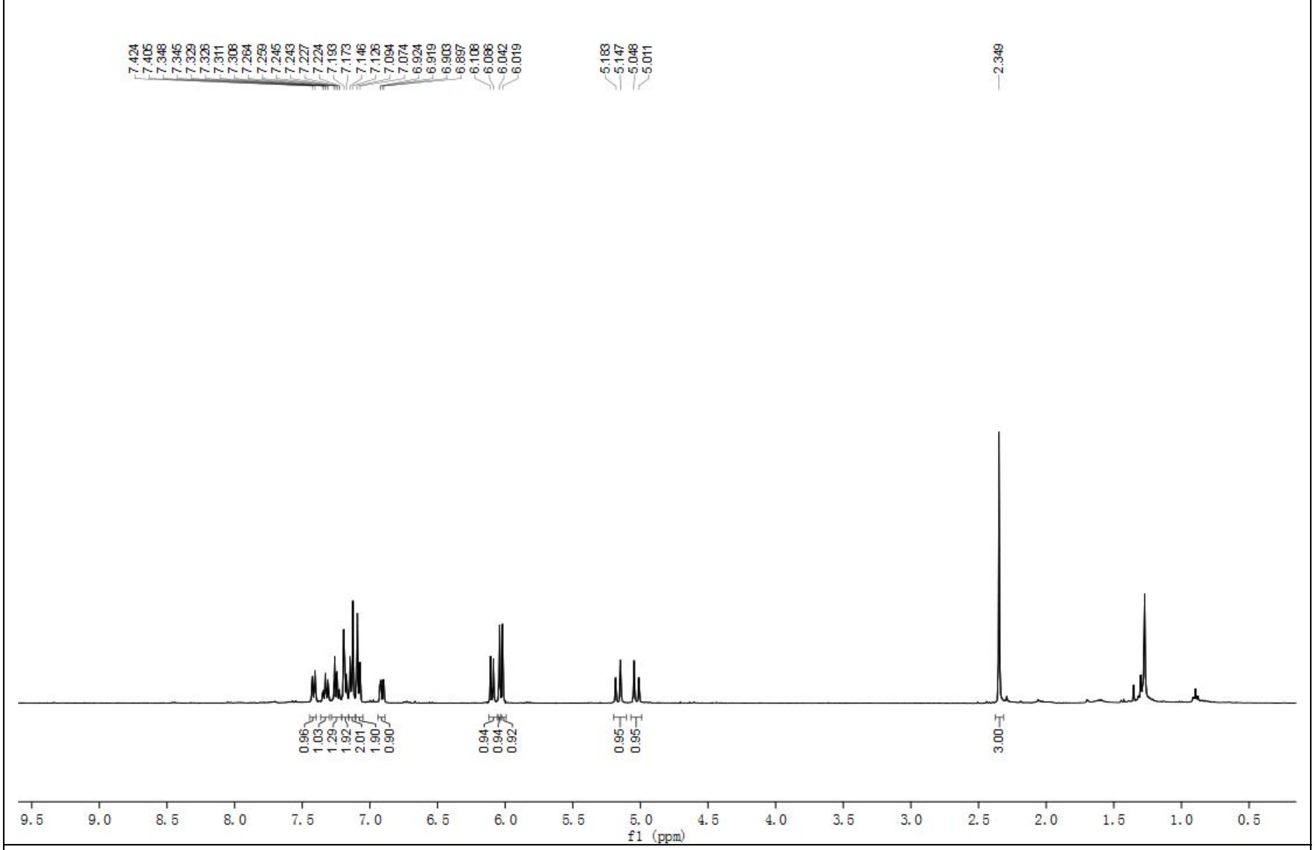

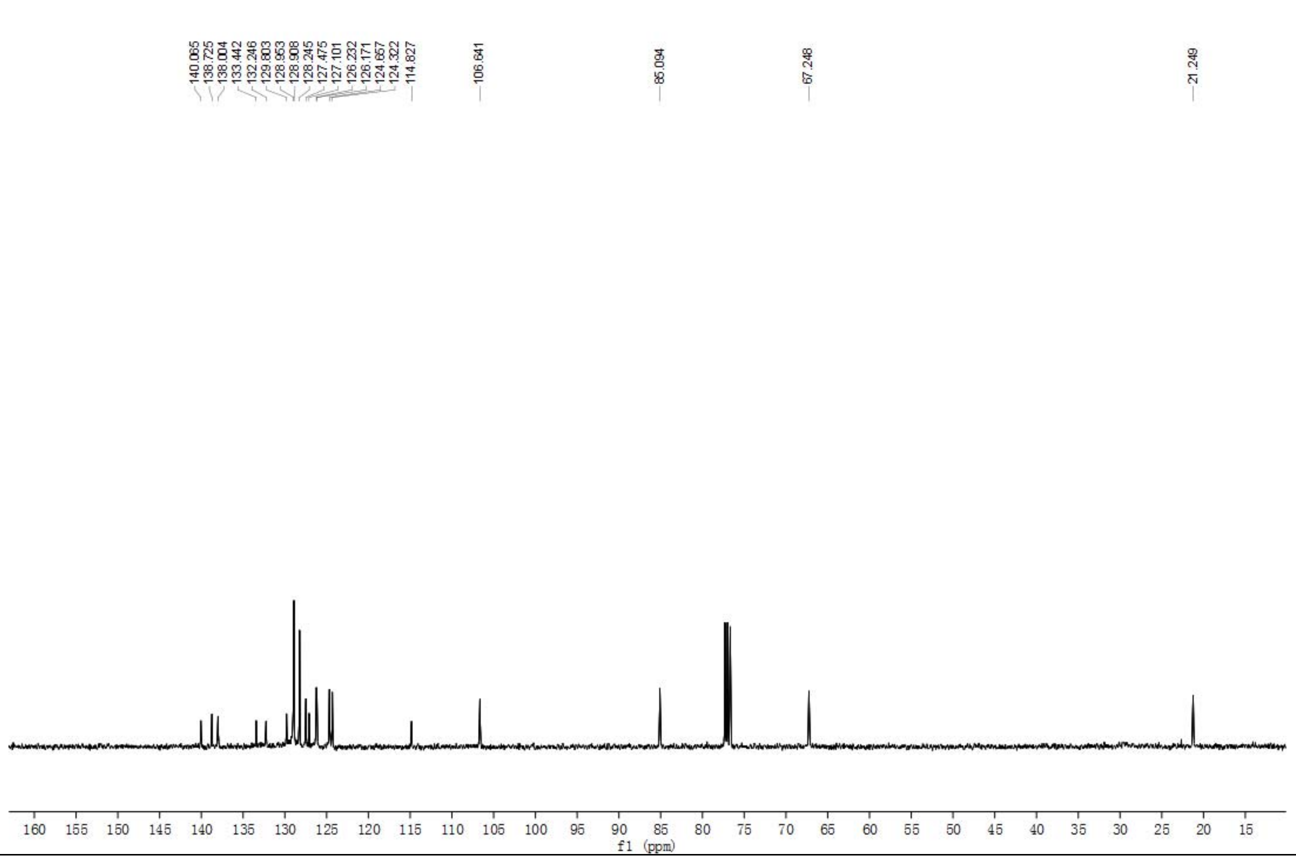

8-Methyl-12-(p-tolyl)-4b,6-dihydrobenzo[4,5][1,3]oxazino[2,3-*a*]isoquinoline (3Df)

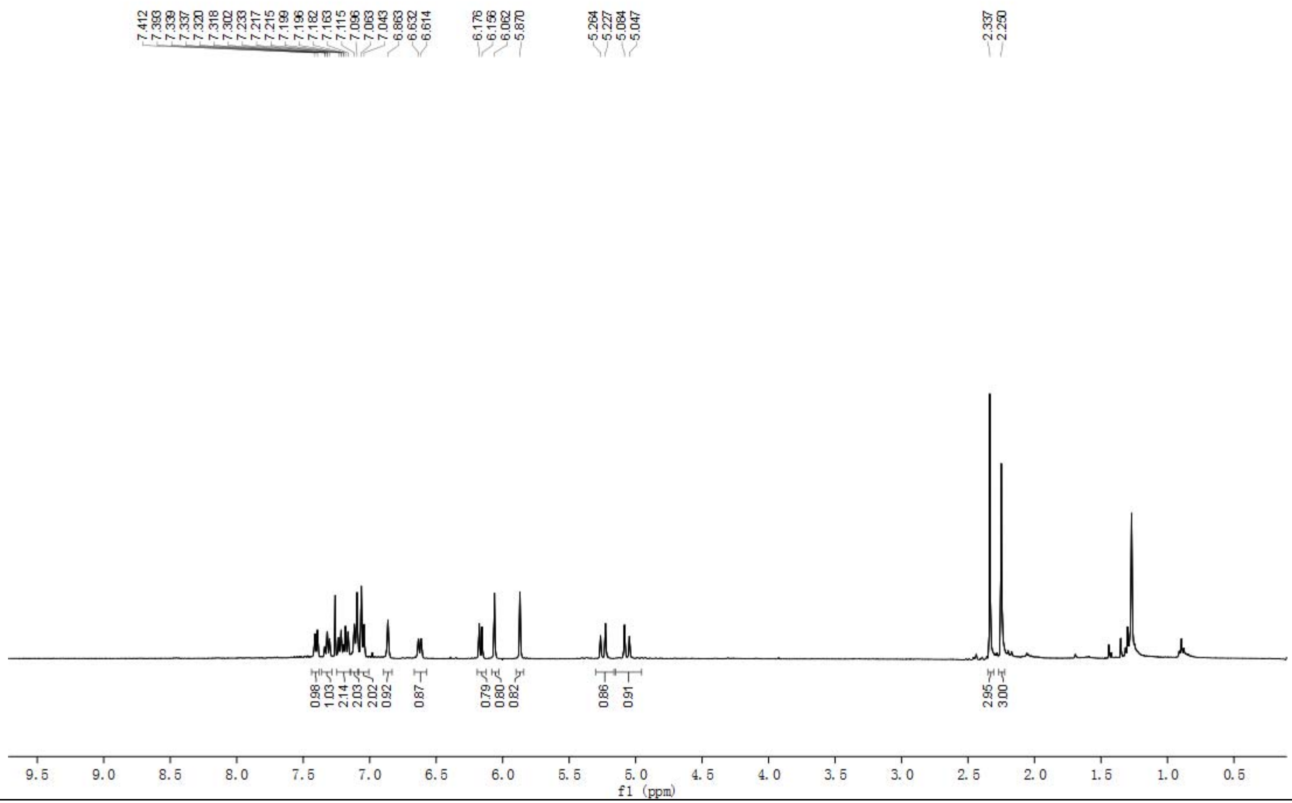

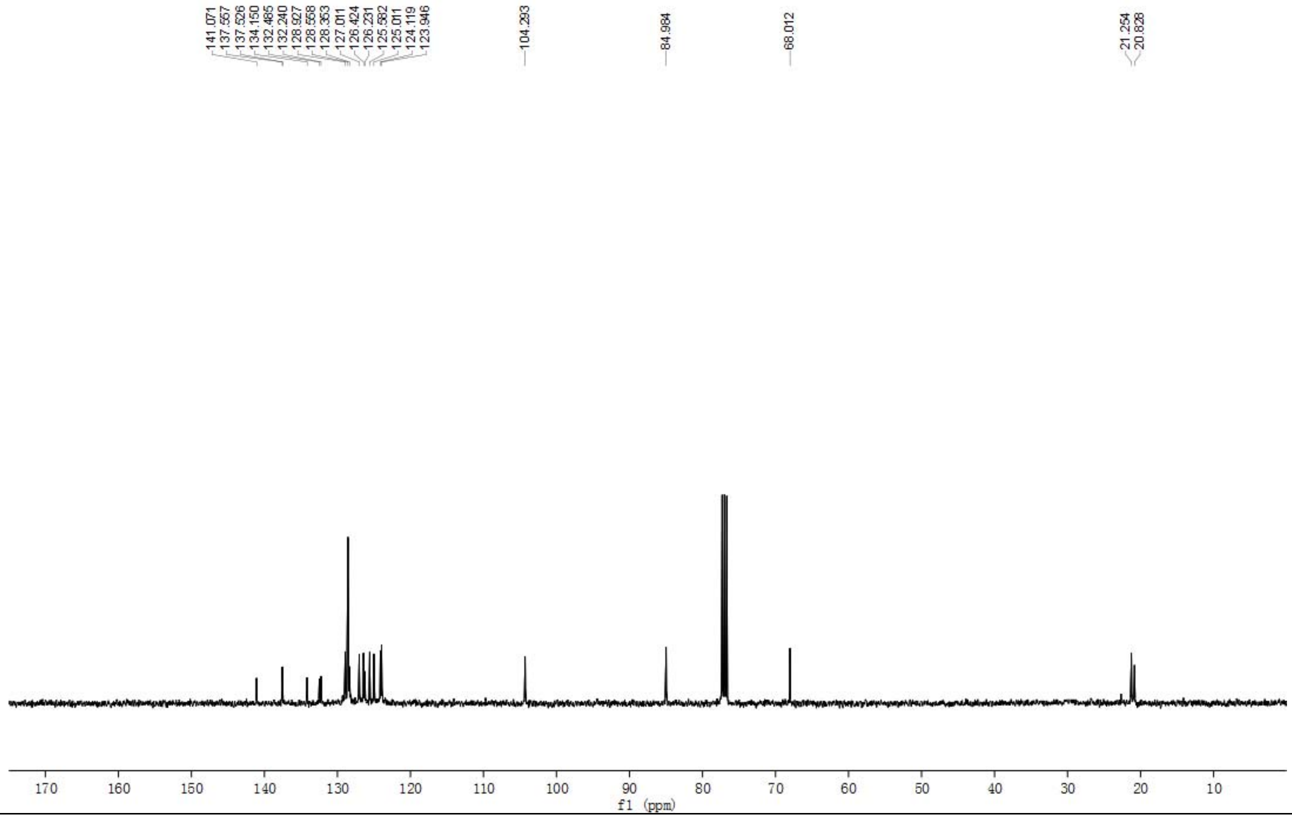

**14-(*p*-Tolyl)-4b,6-dihydronaphtho[2',3':4,5][1,3]oxazino[2,3-*a*]isoquinoline (3Dh)**

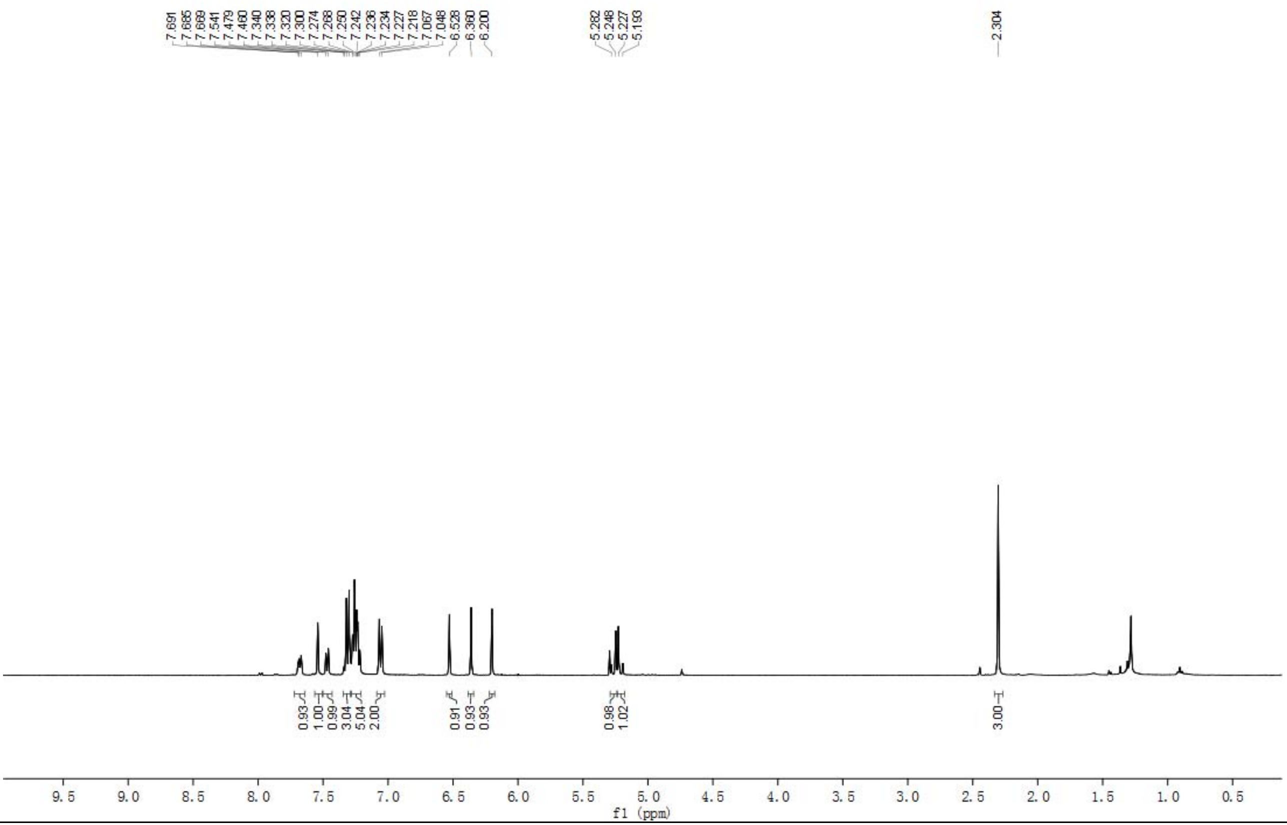

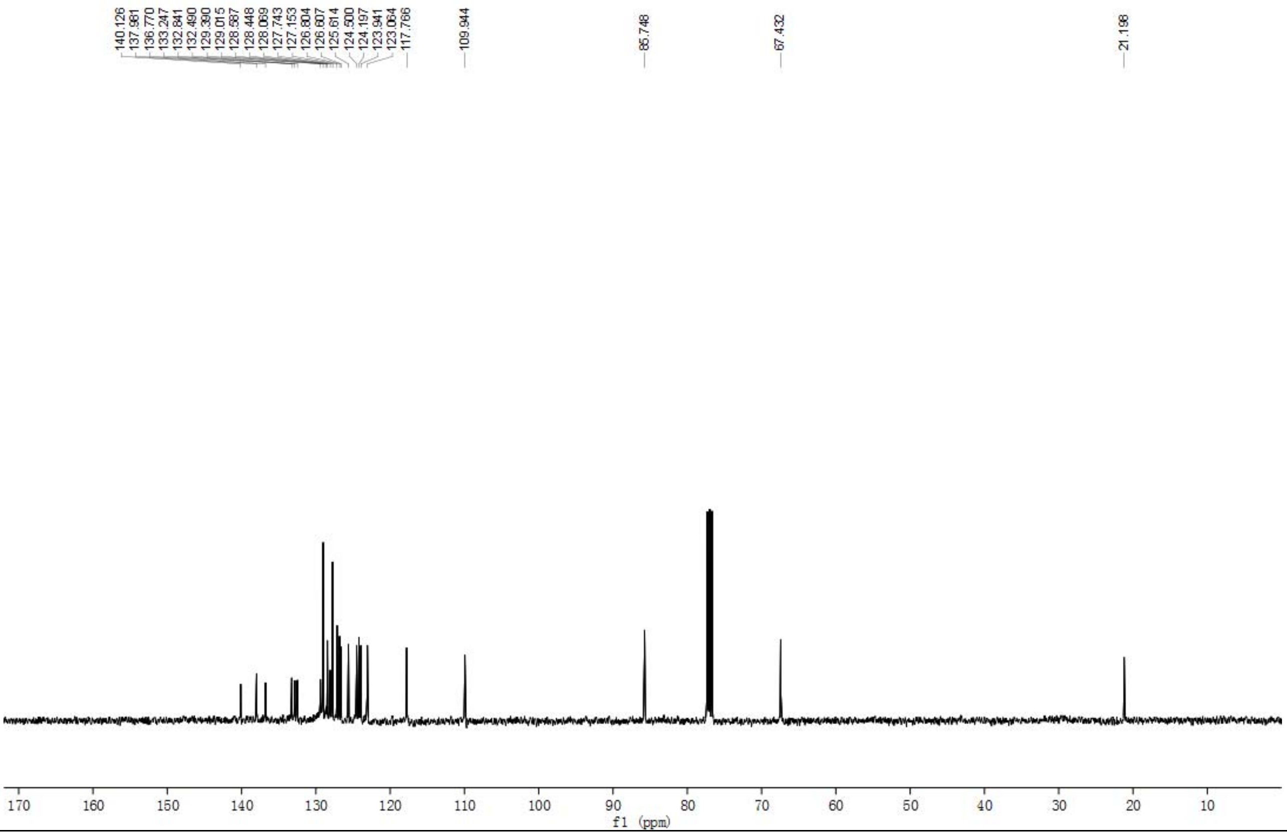

12-(*m*-Tolyl)-4b,6-dihydrobenzo[4,5][1,3]oxazino[2,3-*a*]isoquinoline (3Ea)

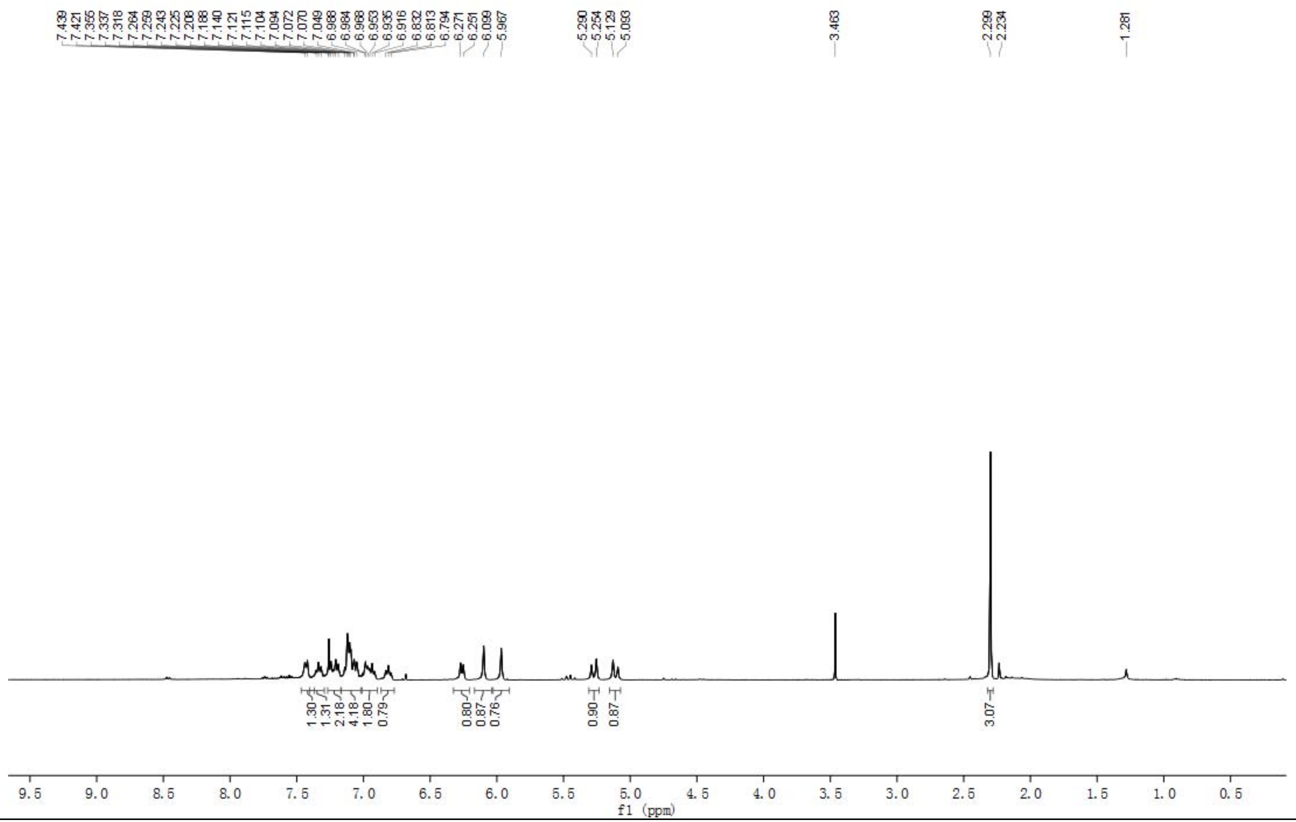

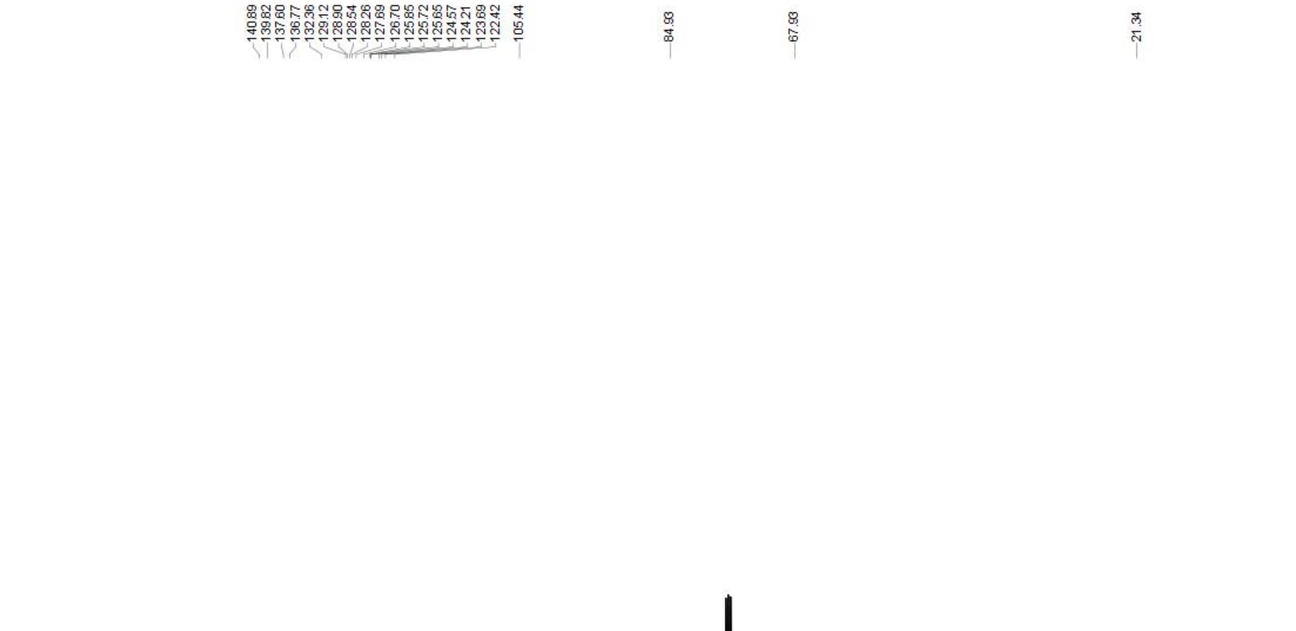

8-Chloro-12-(m-tolyl)-4b,6-dihydrobenzo[4,5][1,3]oxazino[2,3-*a*]isoquinoline (3Ec)

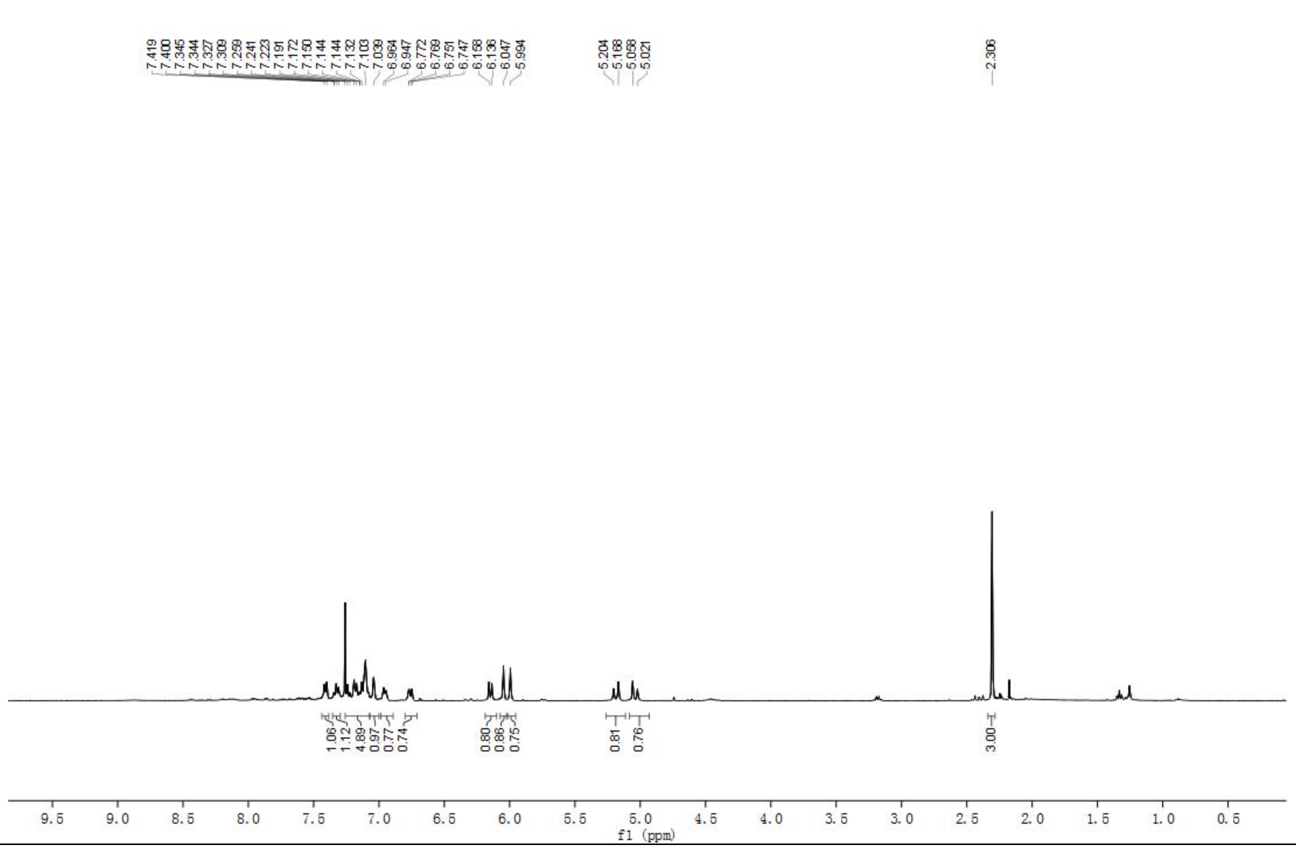

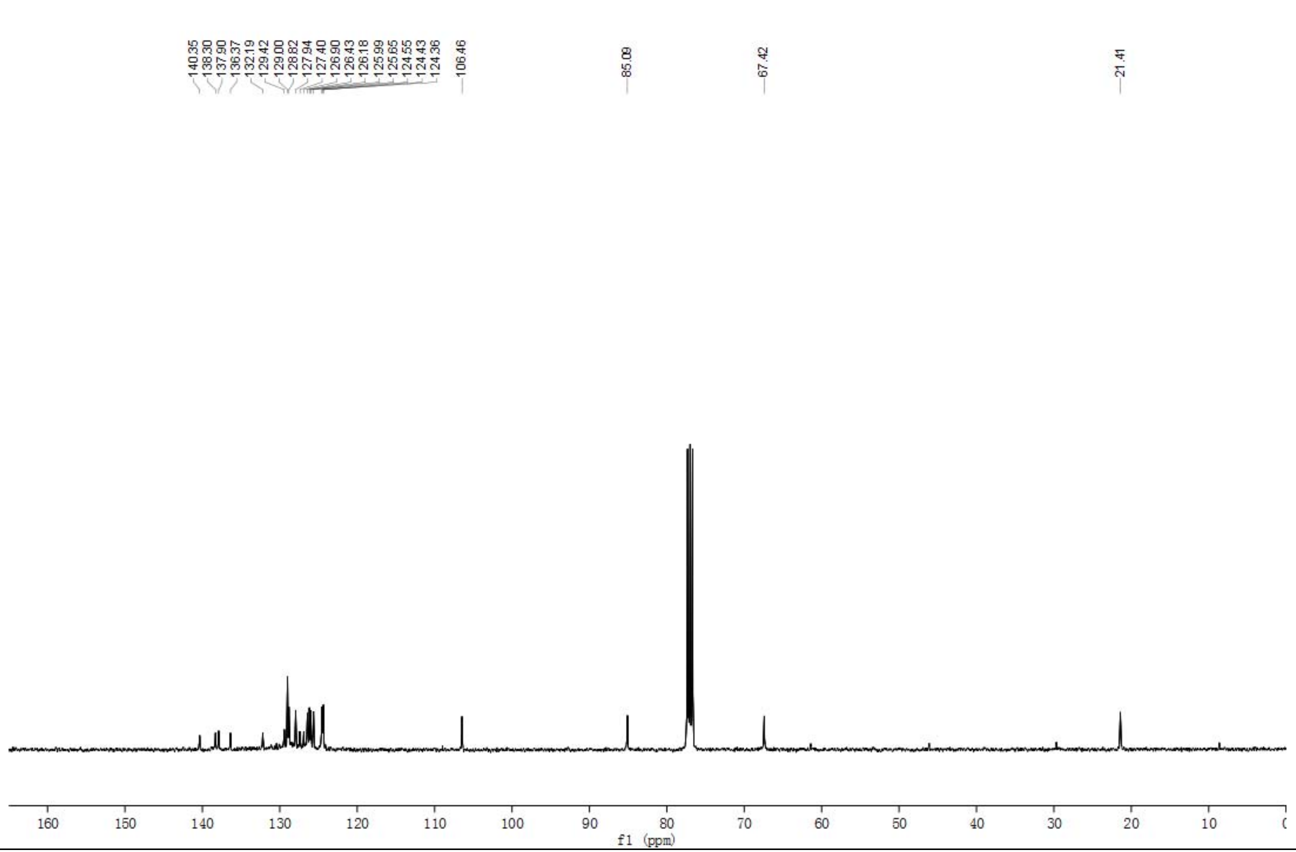

8-Bromo-12-(m-tolyl)-4b,6-dihydrobenzo[4,5][1,3]oxazino[2,3-*a*]isoquinoline (3Ee)

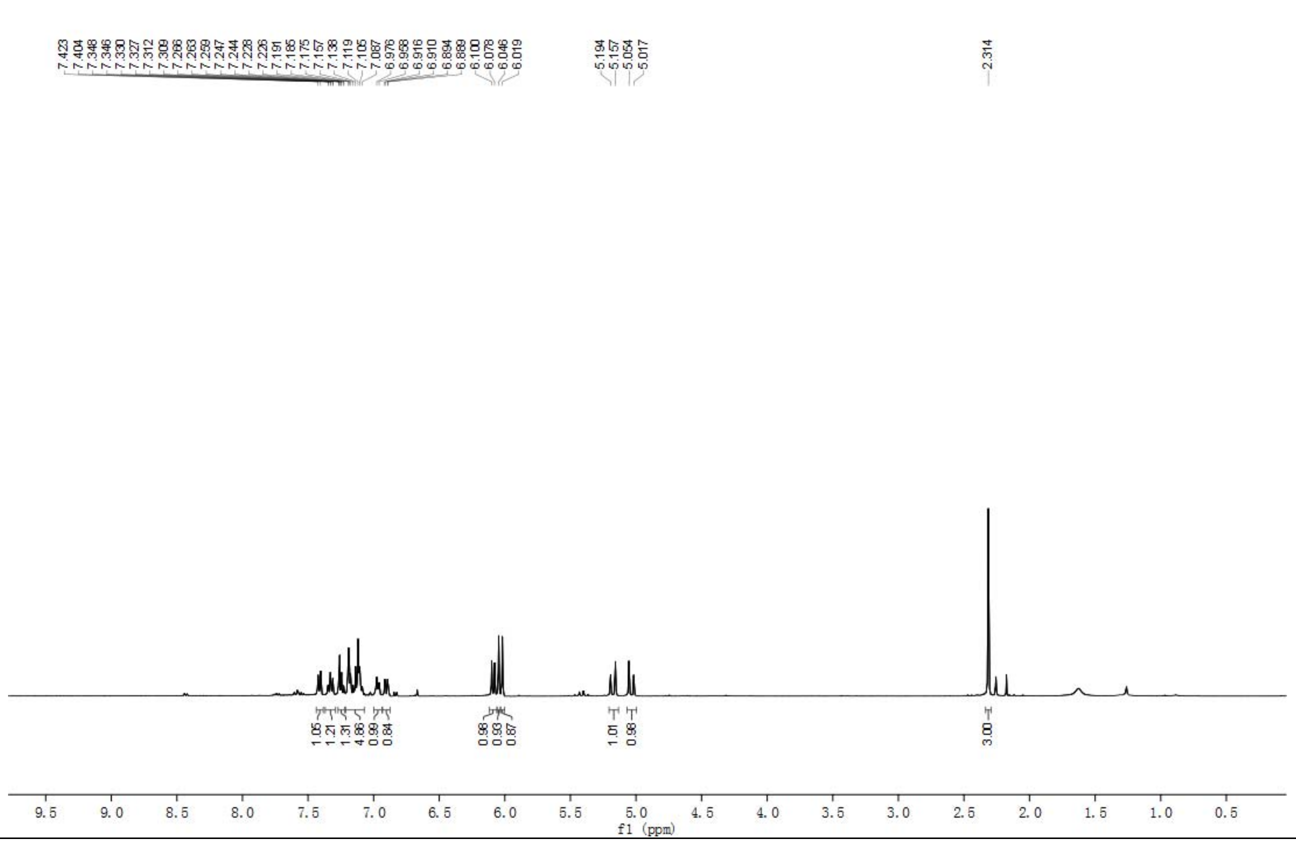

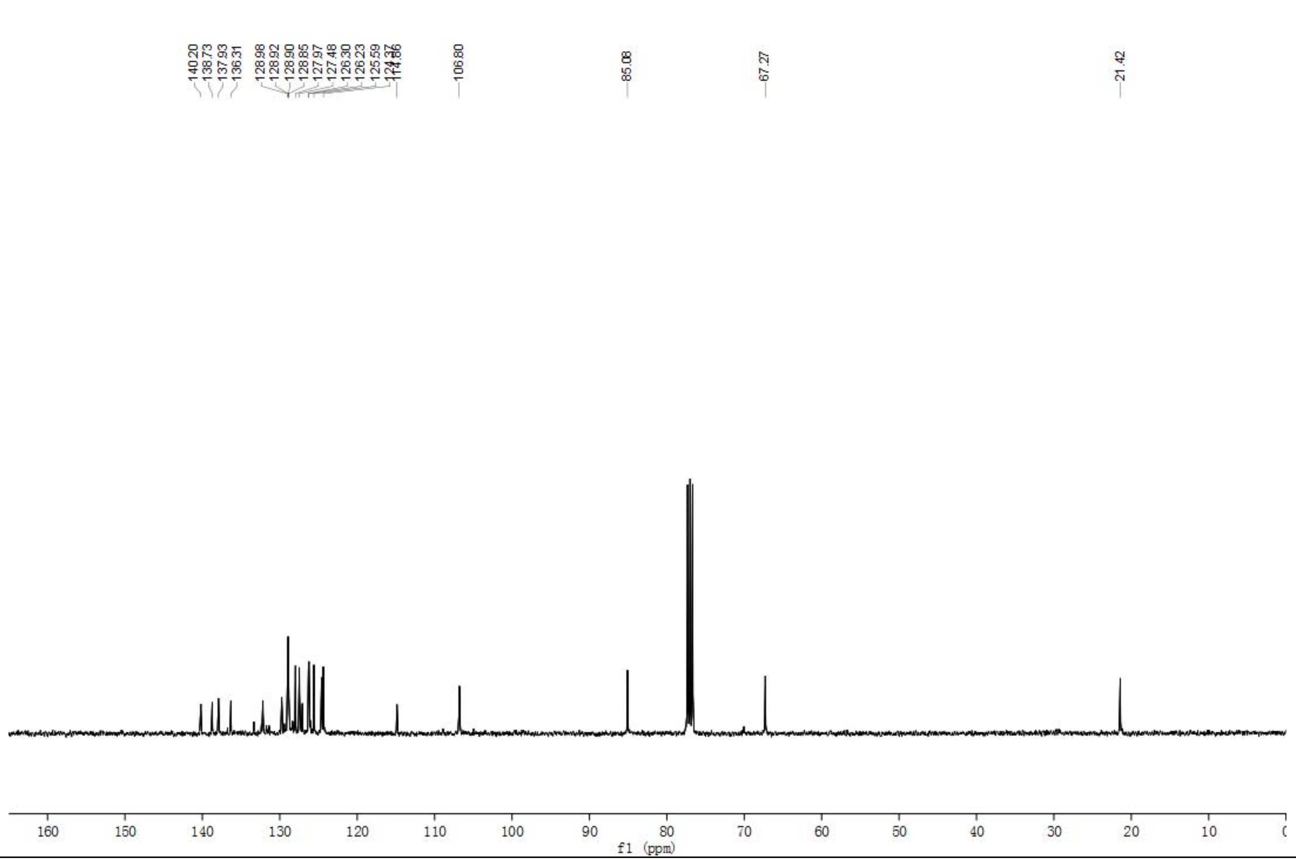

3-Fluoro-12-phenyl-4b,6-dihydrobenzo[4,5][1,3]oxazino[2,3-*a*]isoquinoline (3Fa)

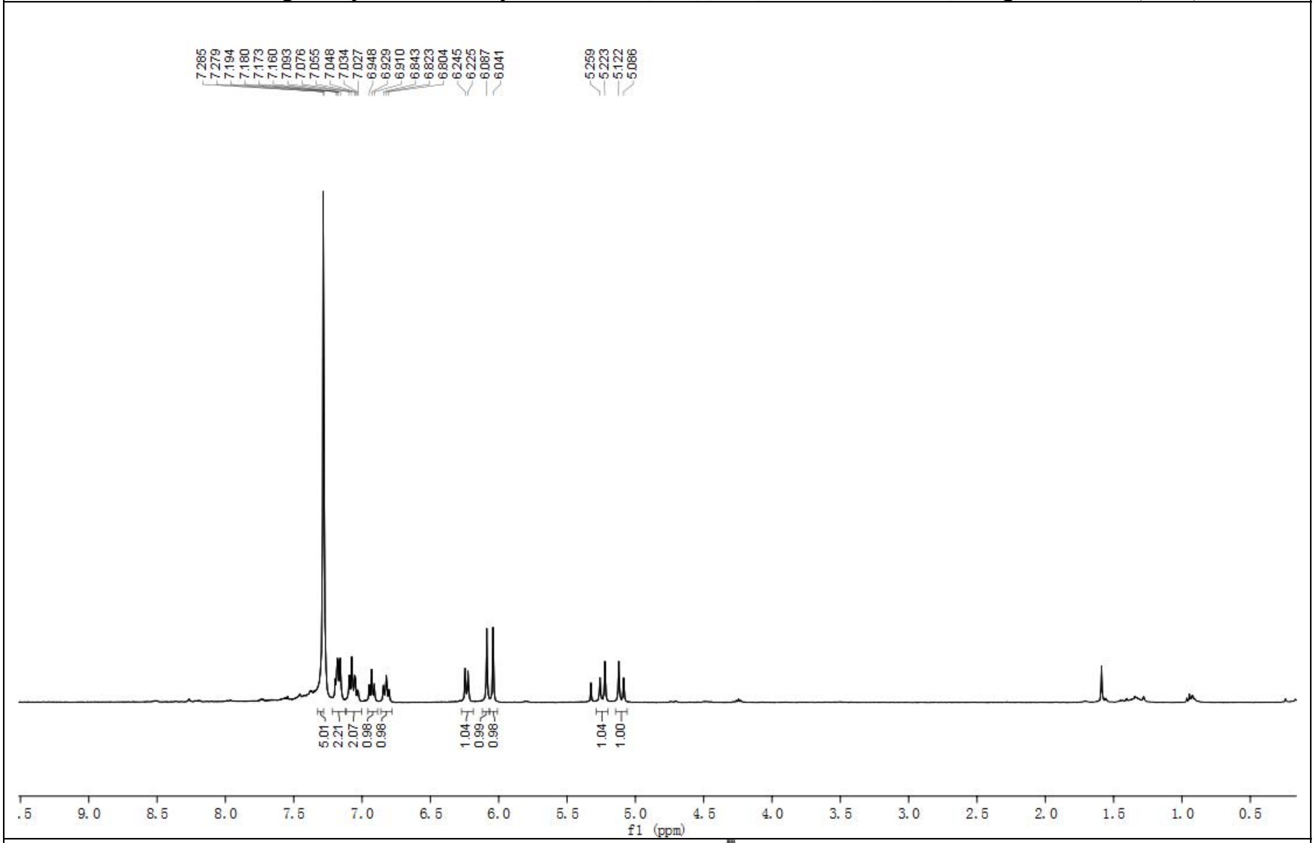

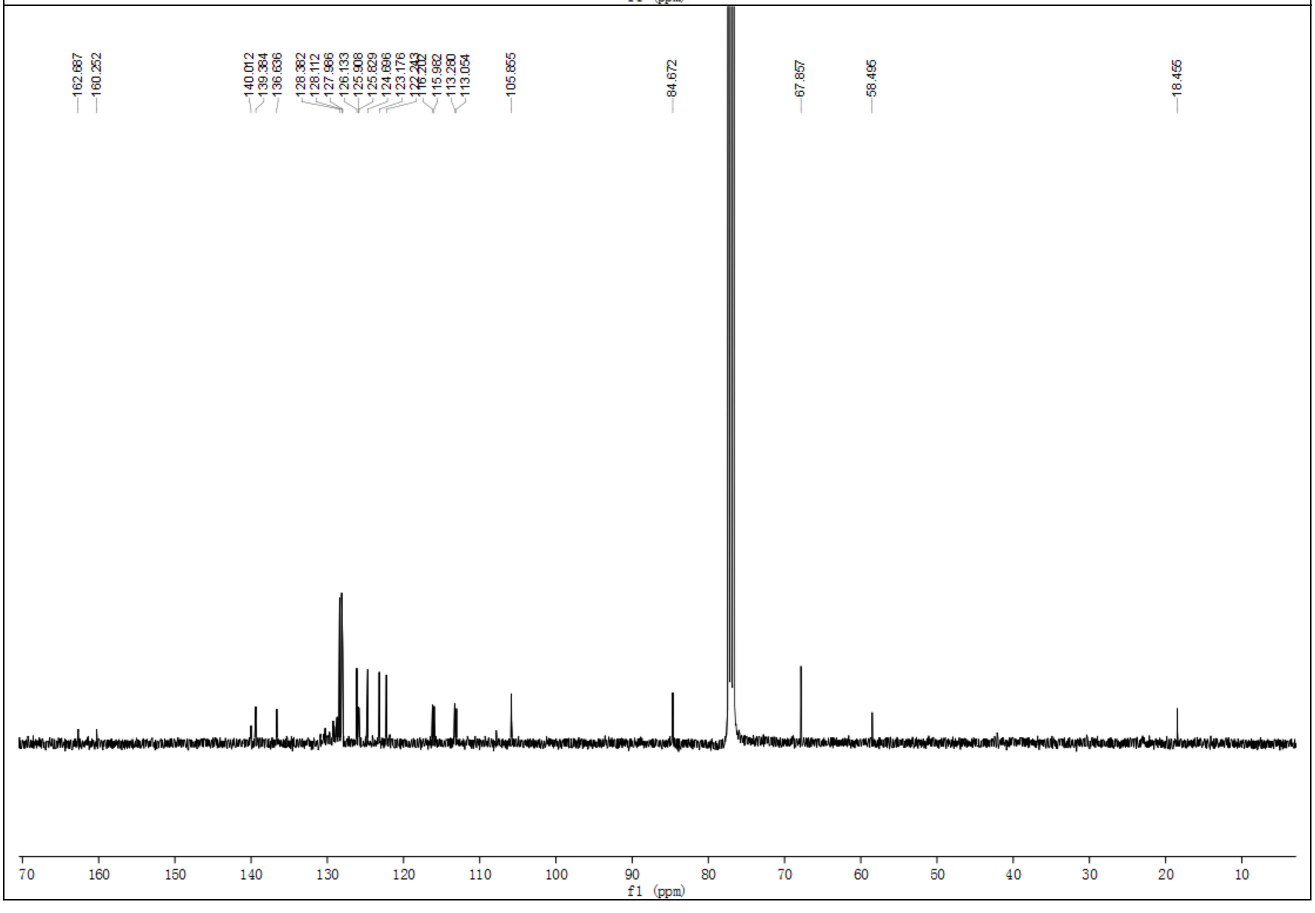

8-Chloro-3-fluoro-12-phenyl-4b,6-dihydrobenzo[4,5][1,3]oxazino[2,3-*a*]isoquinoline (3Fc)

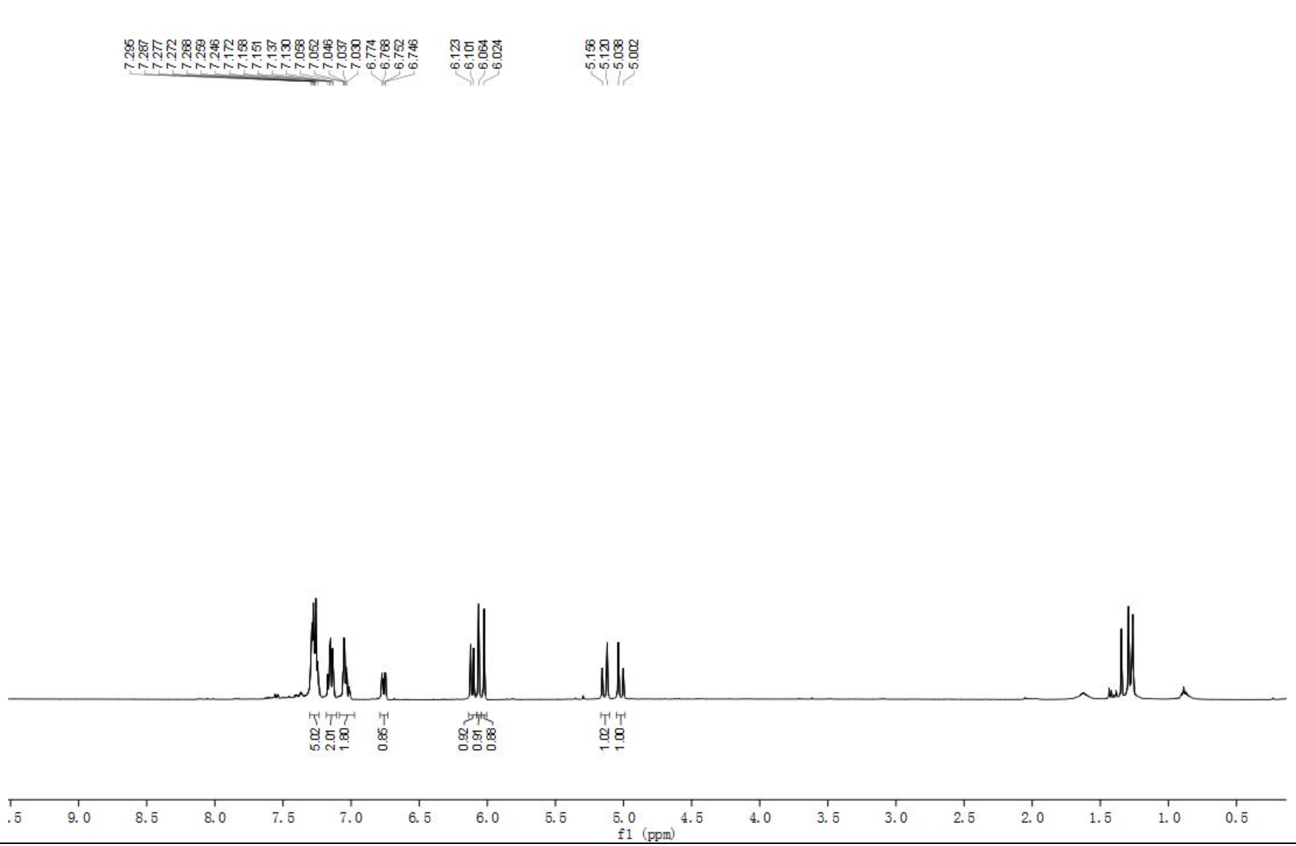

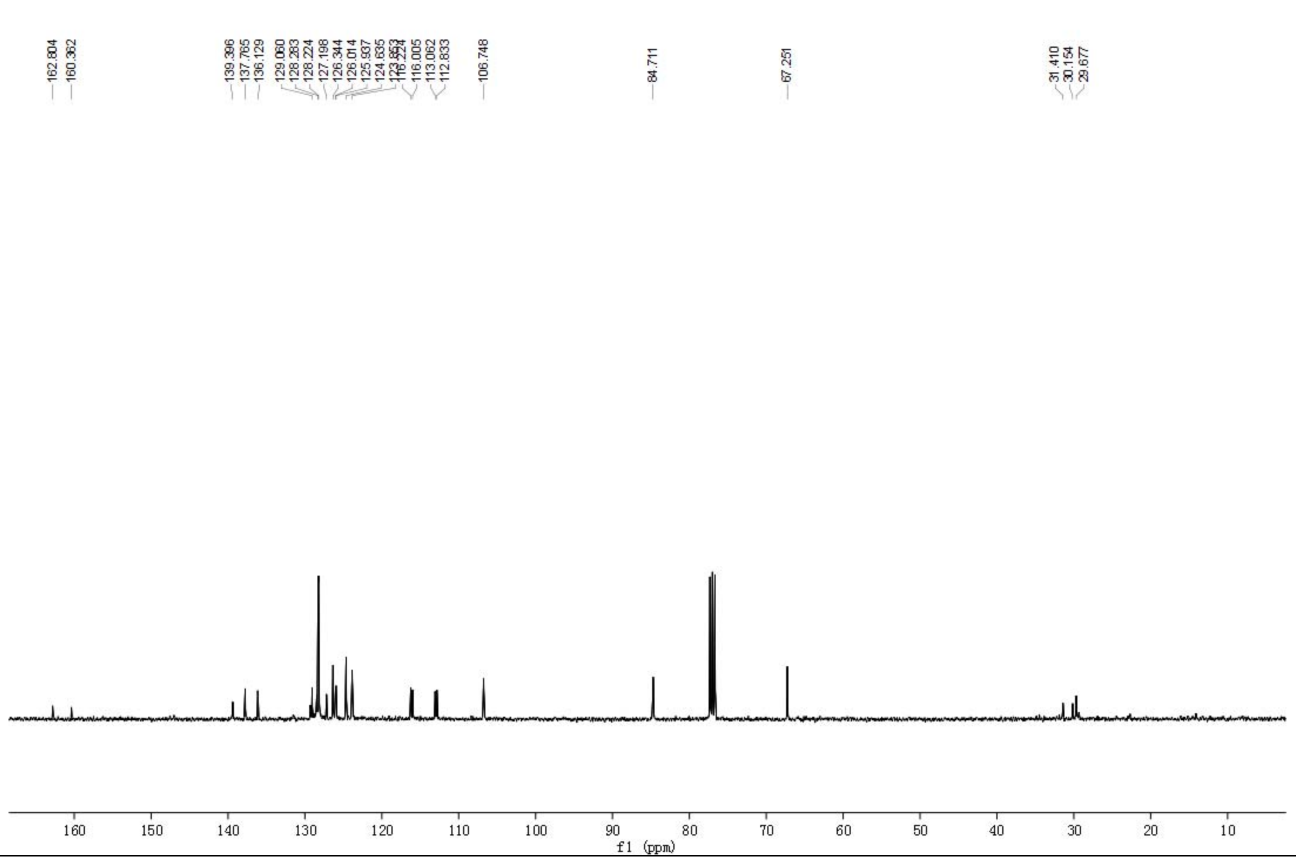

8-Bromo-3-fluoro-12-phenyl-4b,6-dihydrobenzo[4,5][1,3]oxazino[2,3-*a*]isoquinoline (3Fe)

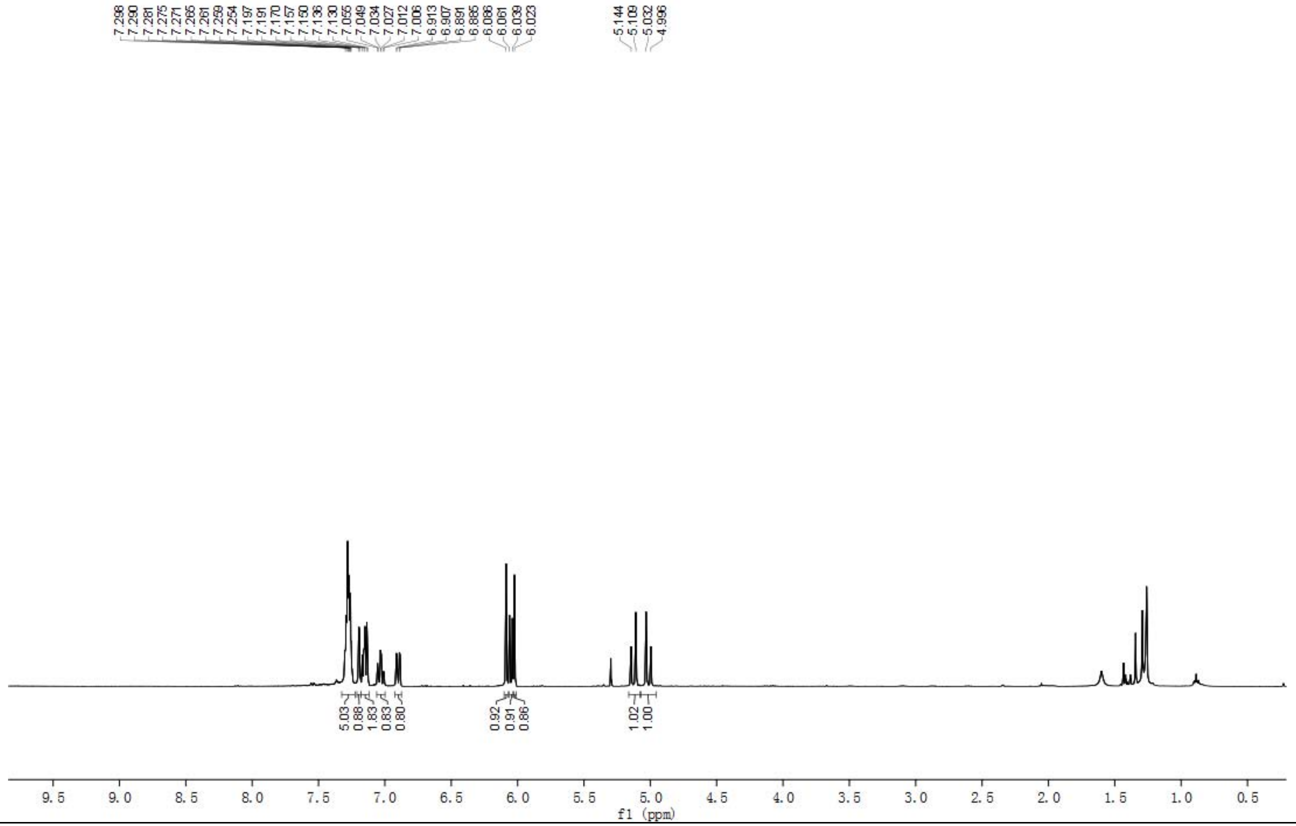

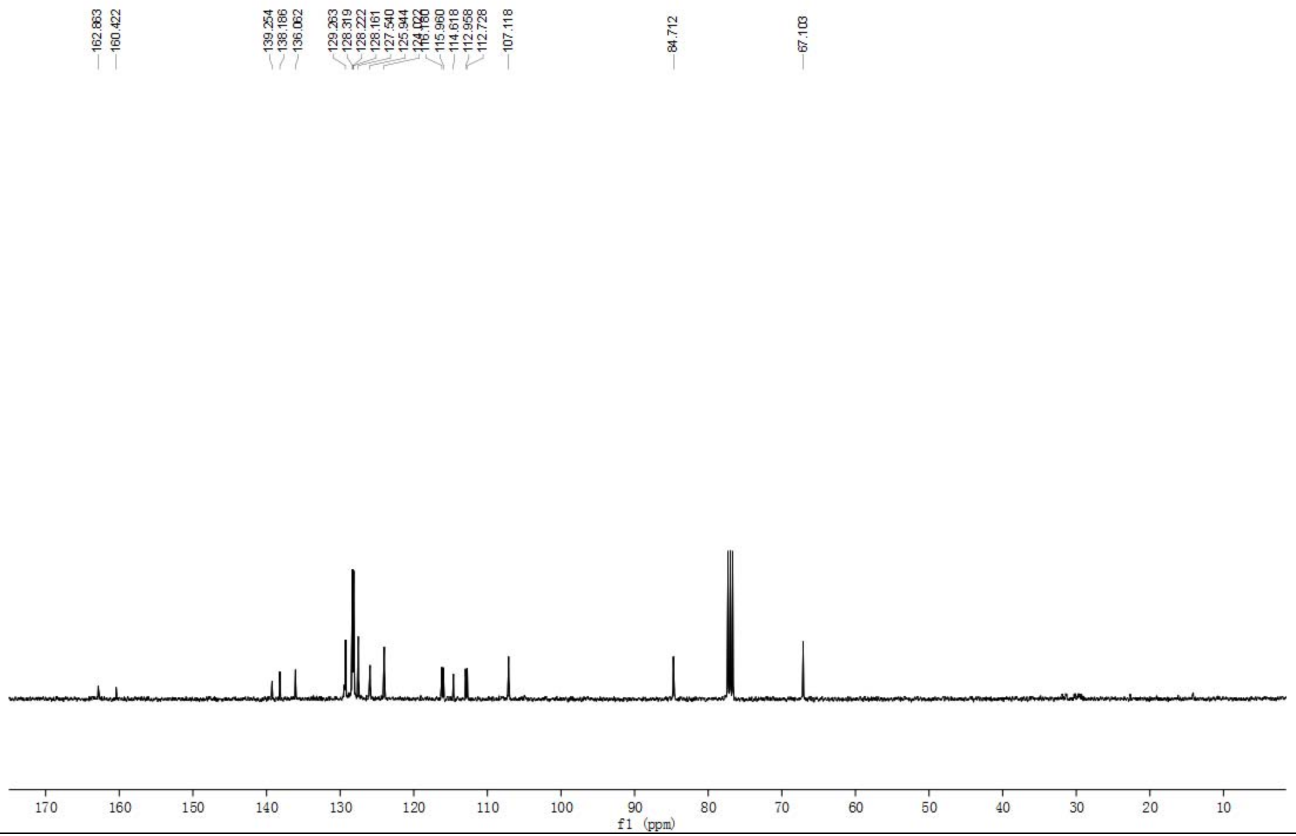

3-Fluoro-8-methyl-12-phenyl-4b,6-dihydrobenzo[4,5][1,3]oxazino[2,3-*a*]isoquinoline (3Ff)

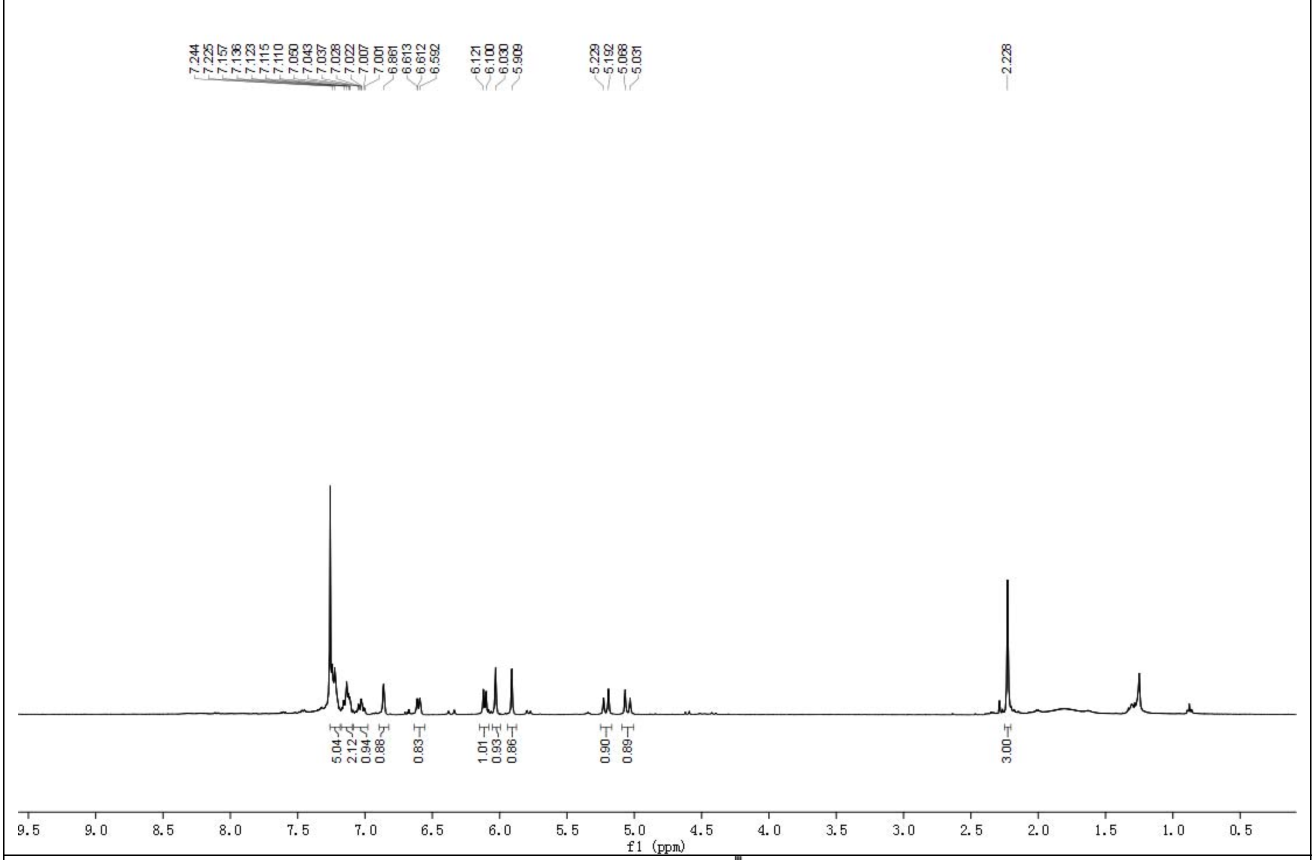

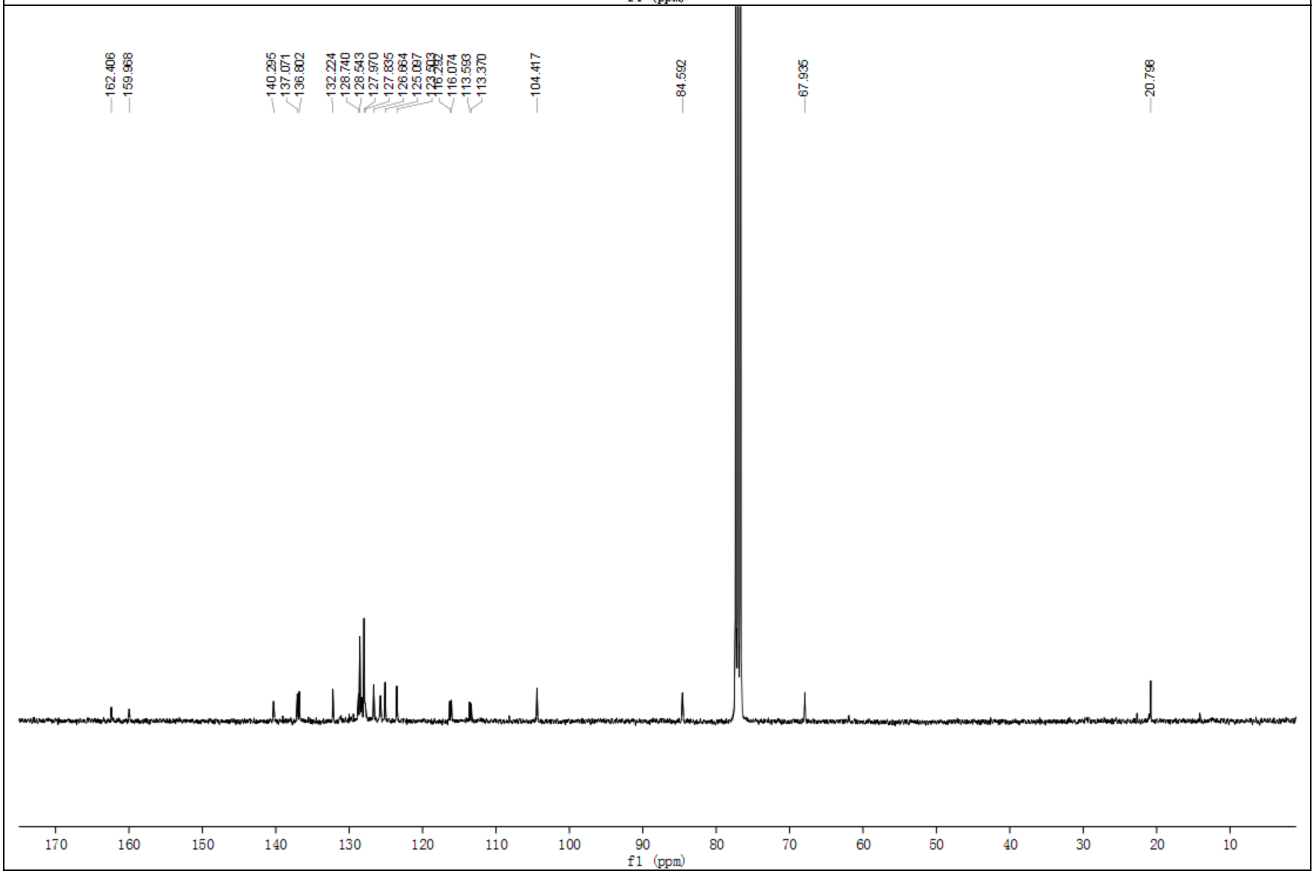

2-Methyl-12-phenyl-4b,6-dihydrobenzo[4,5][1,3]oxazino[2,3-*a*]isoquinoline (3Ga)

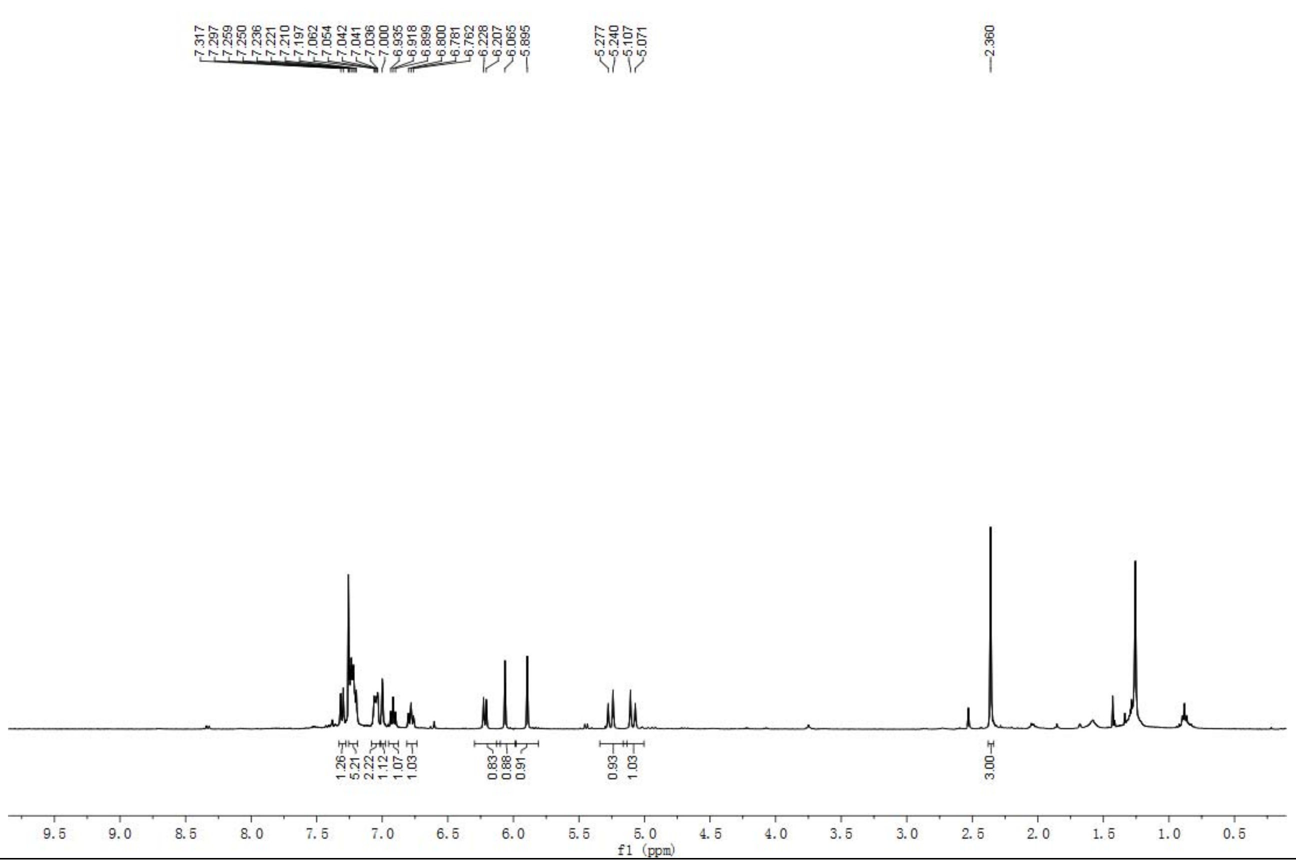

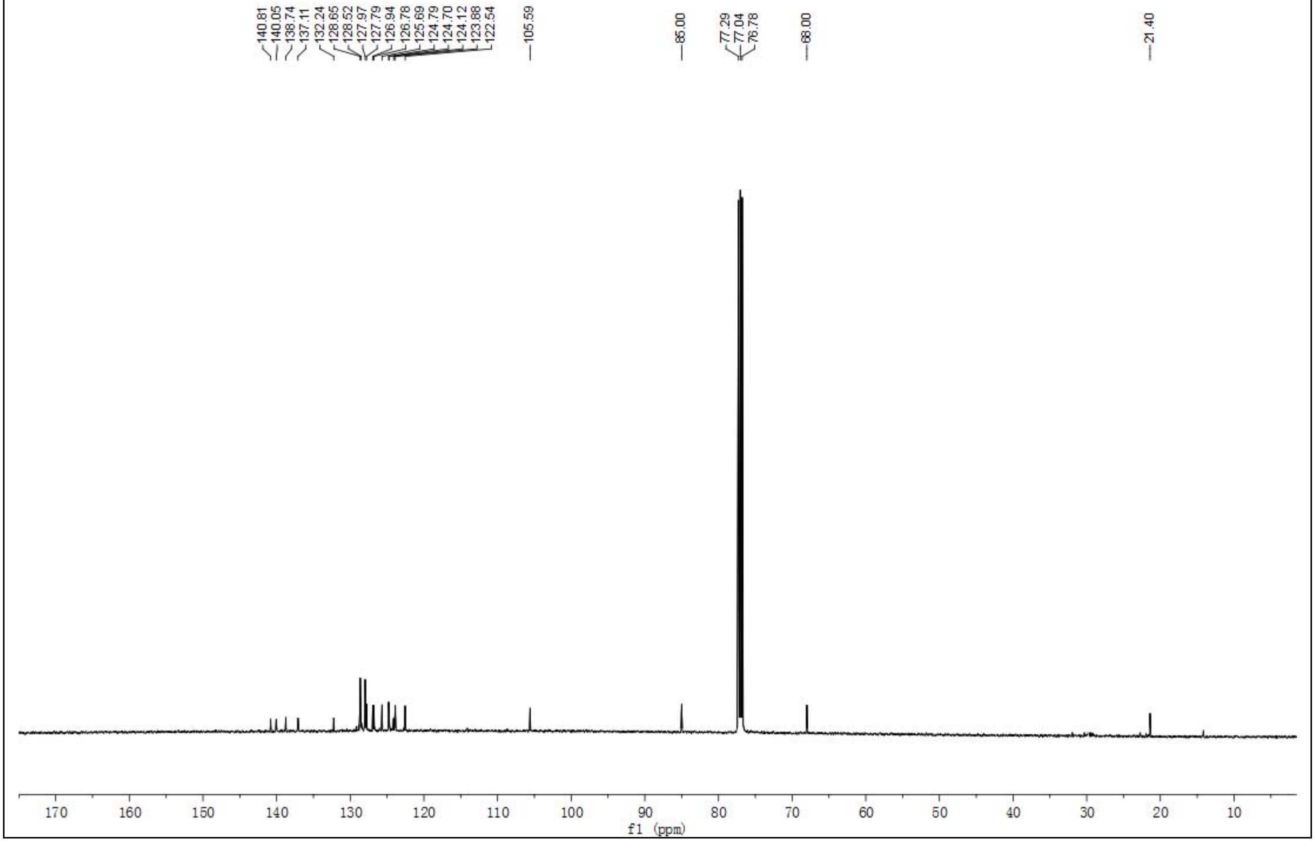

8-Chloro-2-methyl-12-phenyl-4b,6-dihydrobenzo[4,5][1,3]oxazino[2,3-*a*]isoquinoline (3Gc)

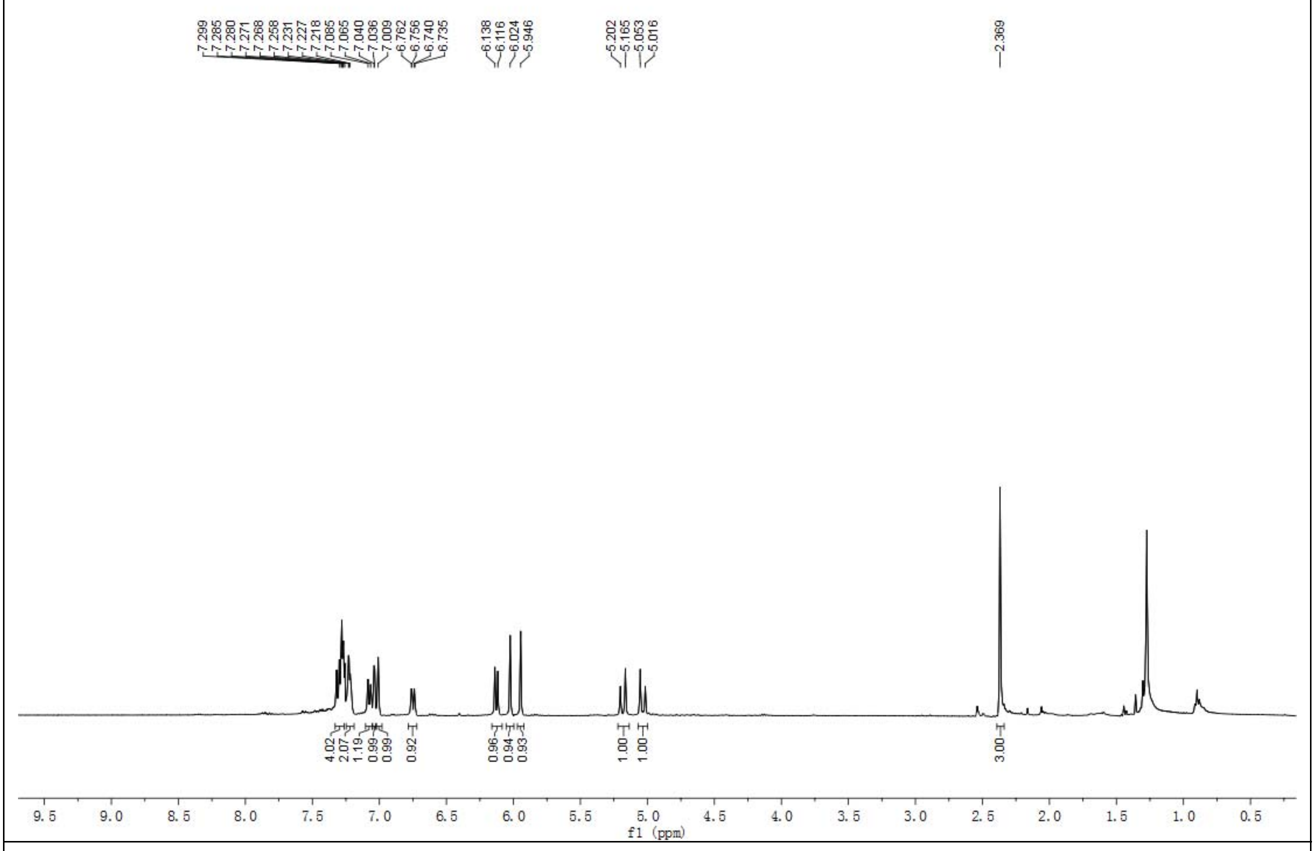

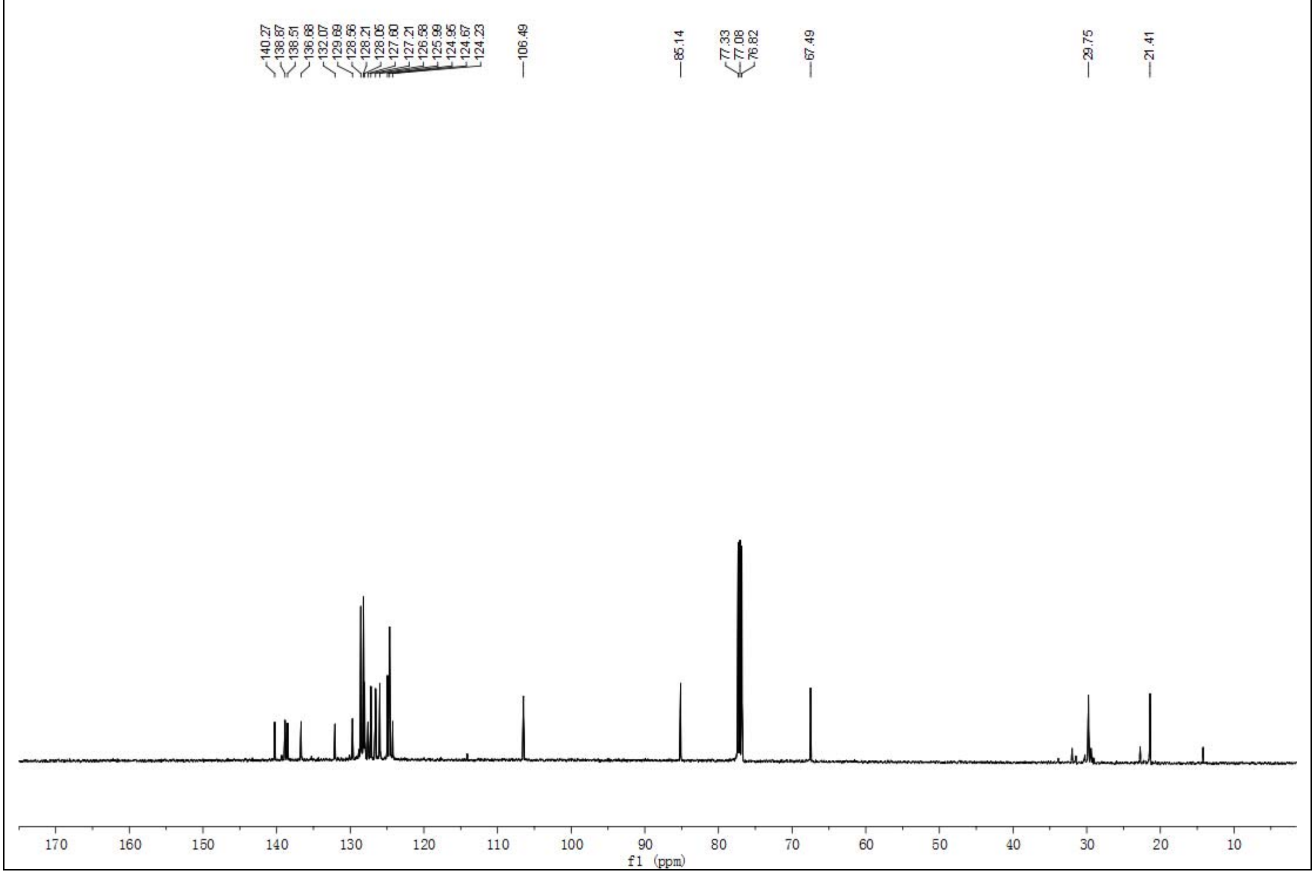

2,8-Dimethyl-12-phenyl-4b,6-dihydrobenzo[4,5][1,3]oxazino[2,3-*a*]isoquinoline (3Gf)

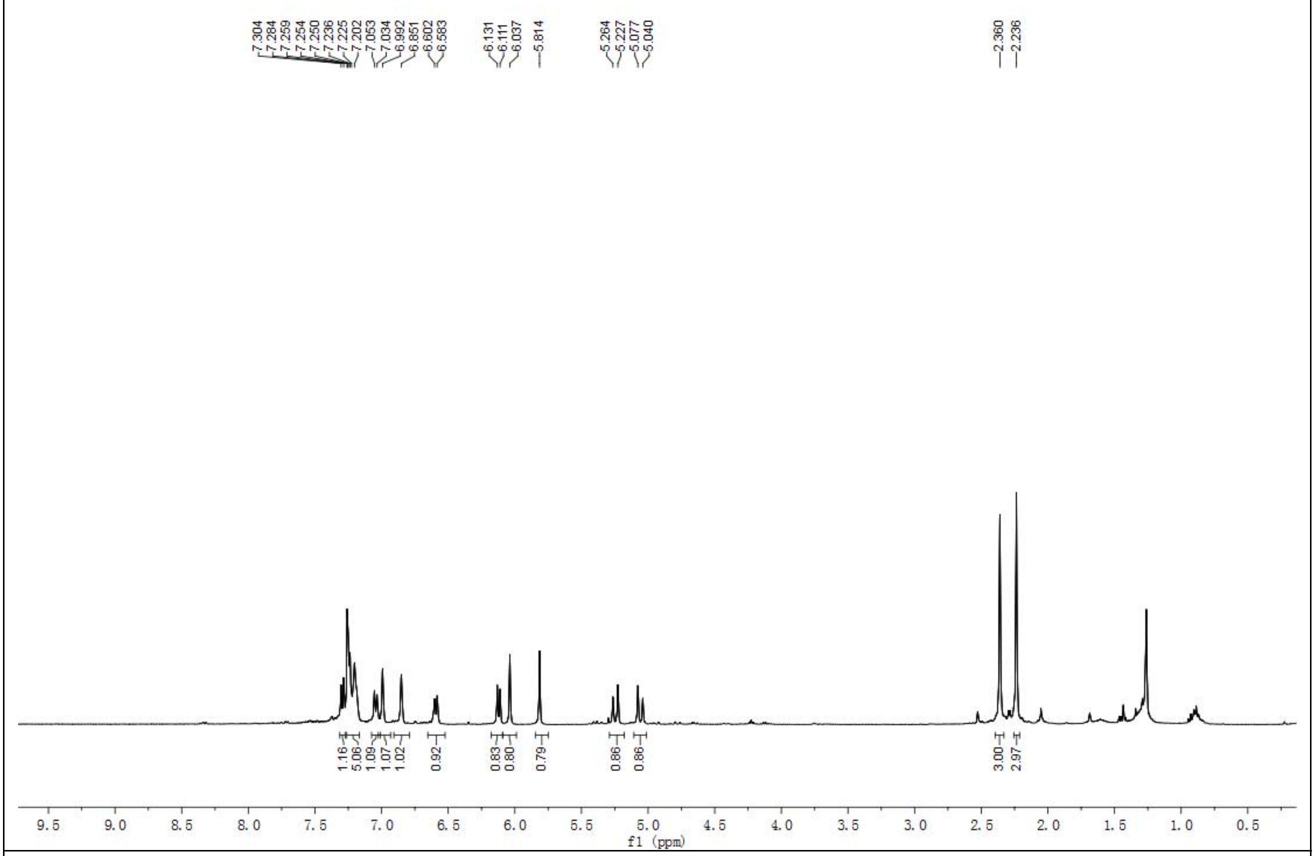

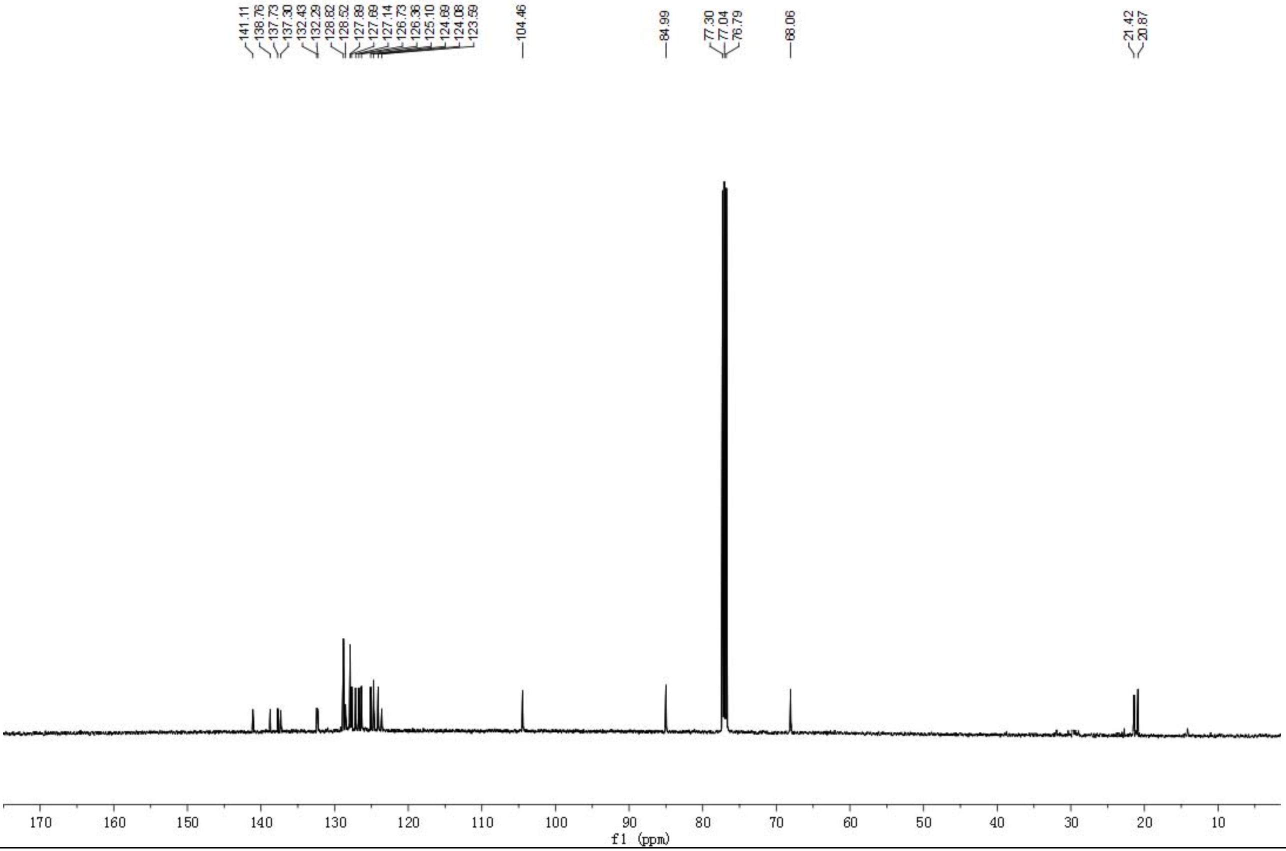

2-Methyl-14-phenyl-4b,6-dihydronaphtho[2',3':4,5][1,3]oxazino[2,3-*a*]isoquinoline (3Gh)

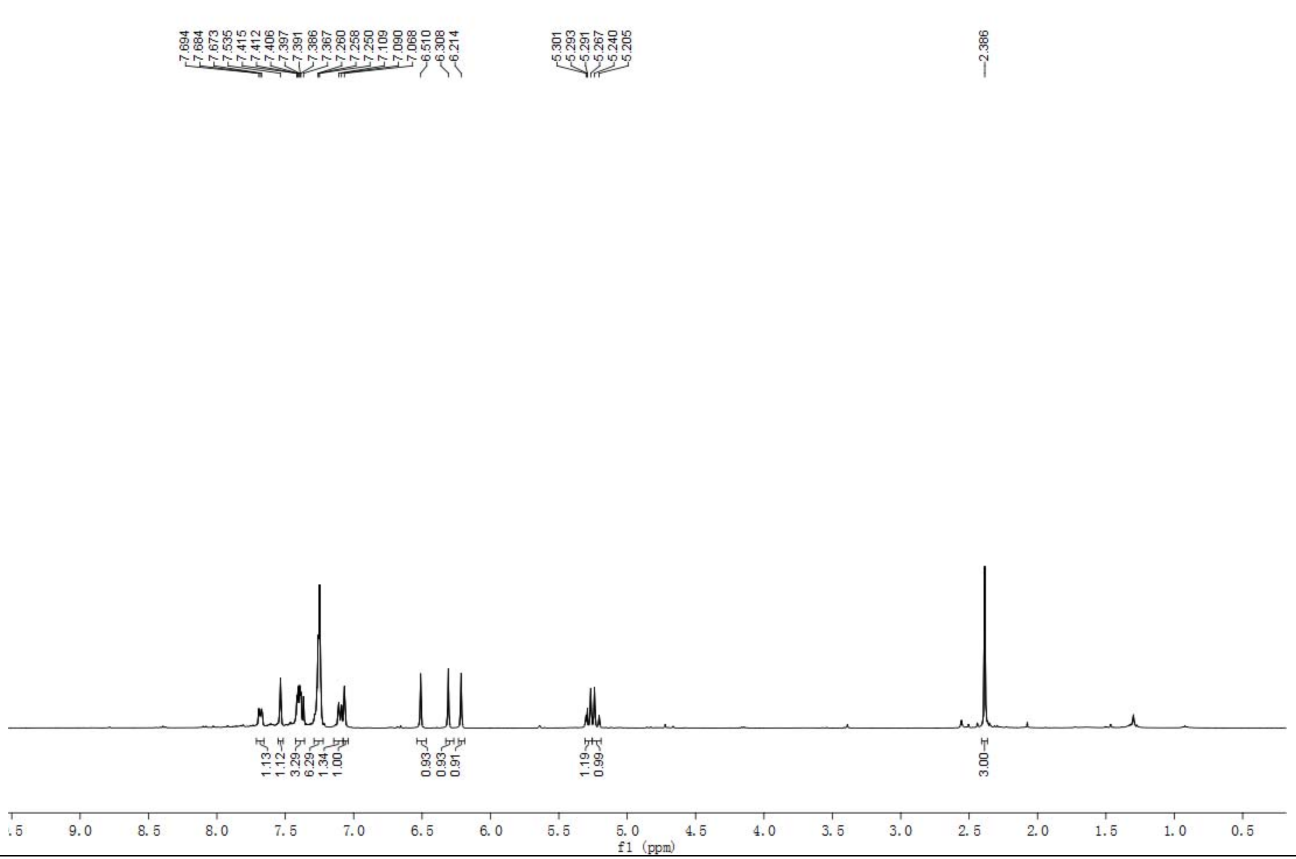

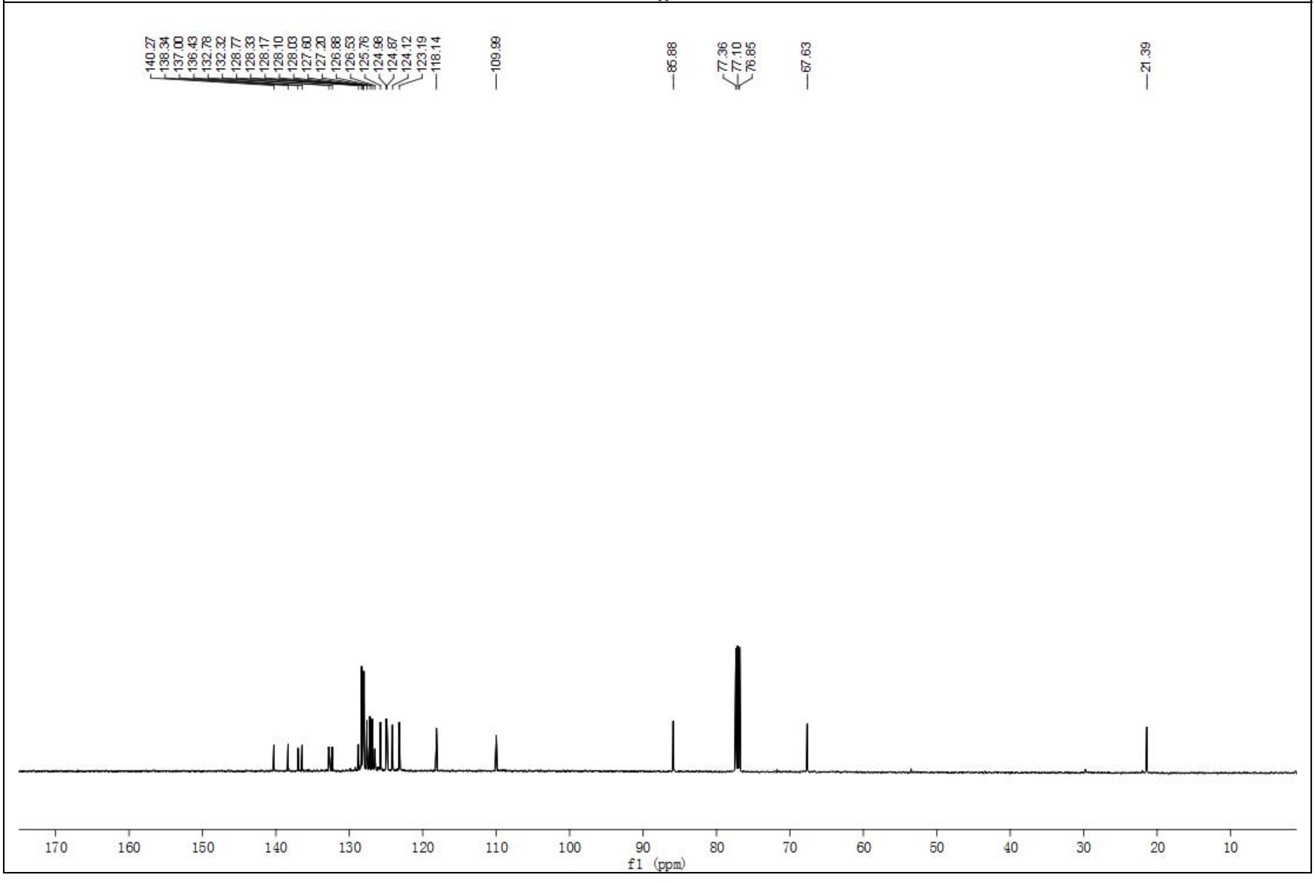

12-Hexyl-4b,6-dihydrobenzo[4,5][1,3]oxazino[2,3-*a*]isoquinoline (3Ha)

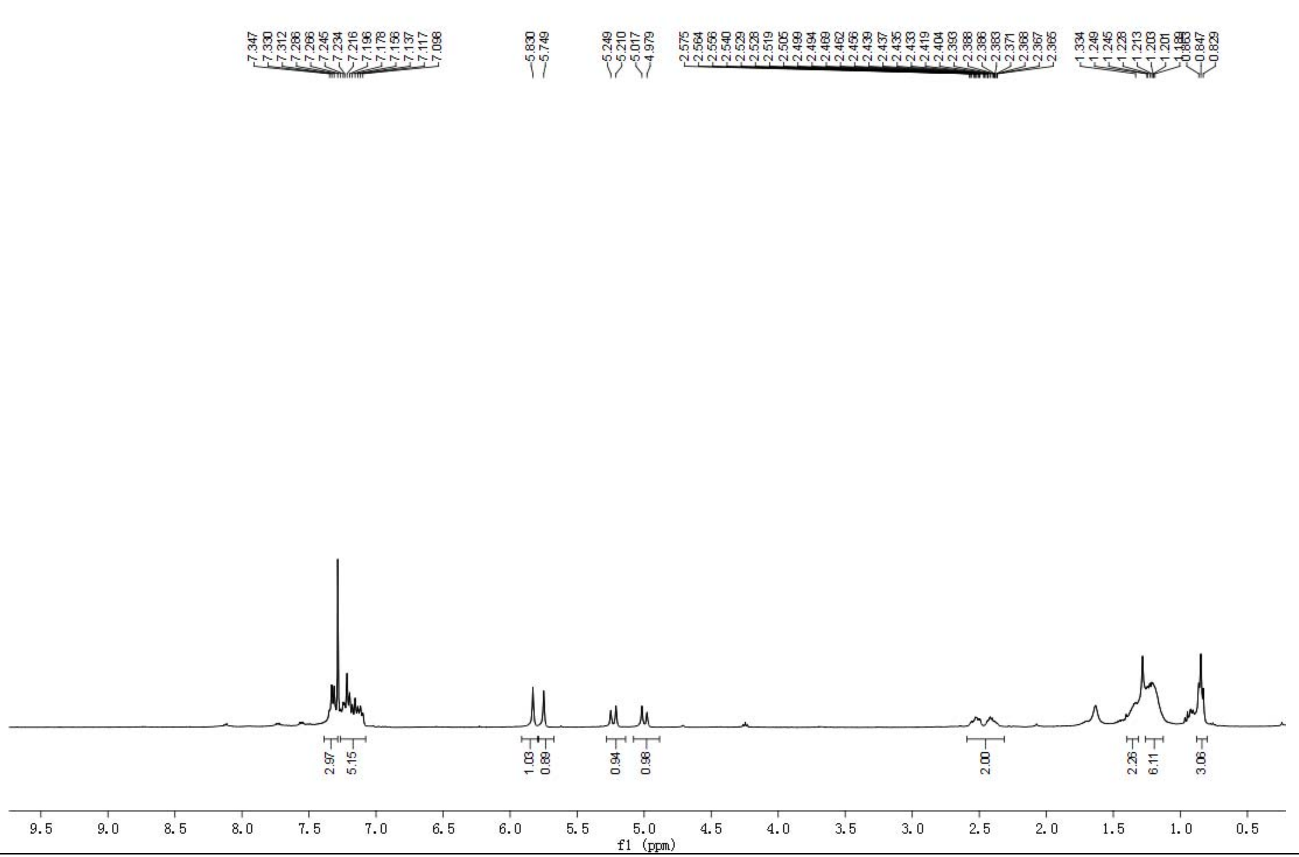

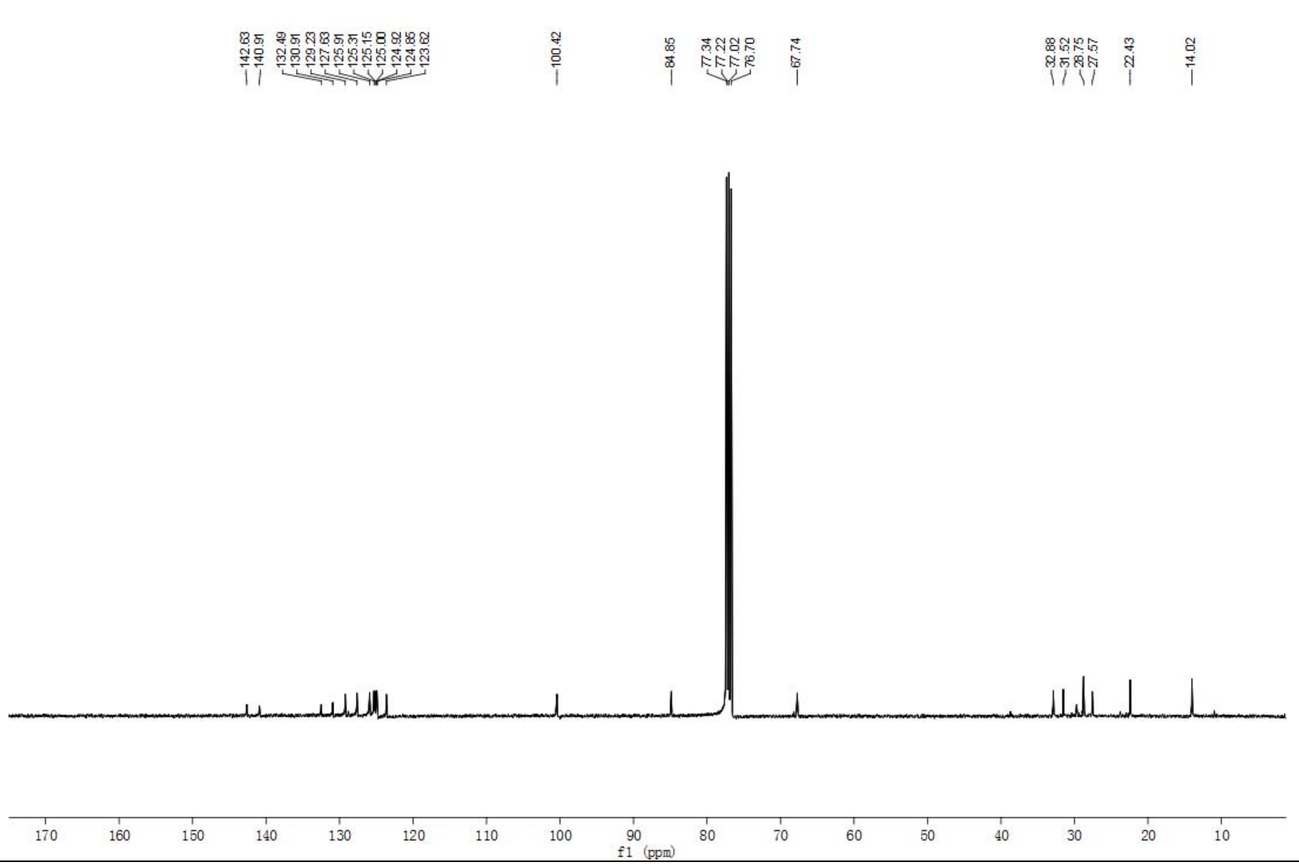

12-Hexyl-8-iodo-4b,6-dihydrobenzo[4,5][1,3]oxazino[2,3-*a*]isoquinoline (3Hi)

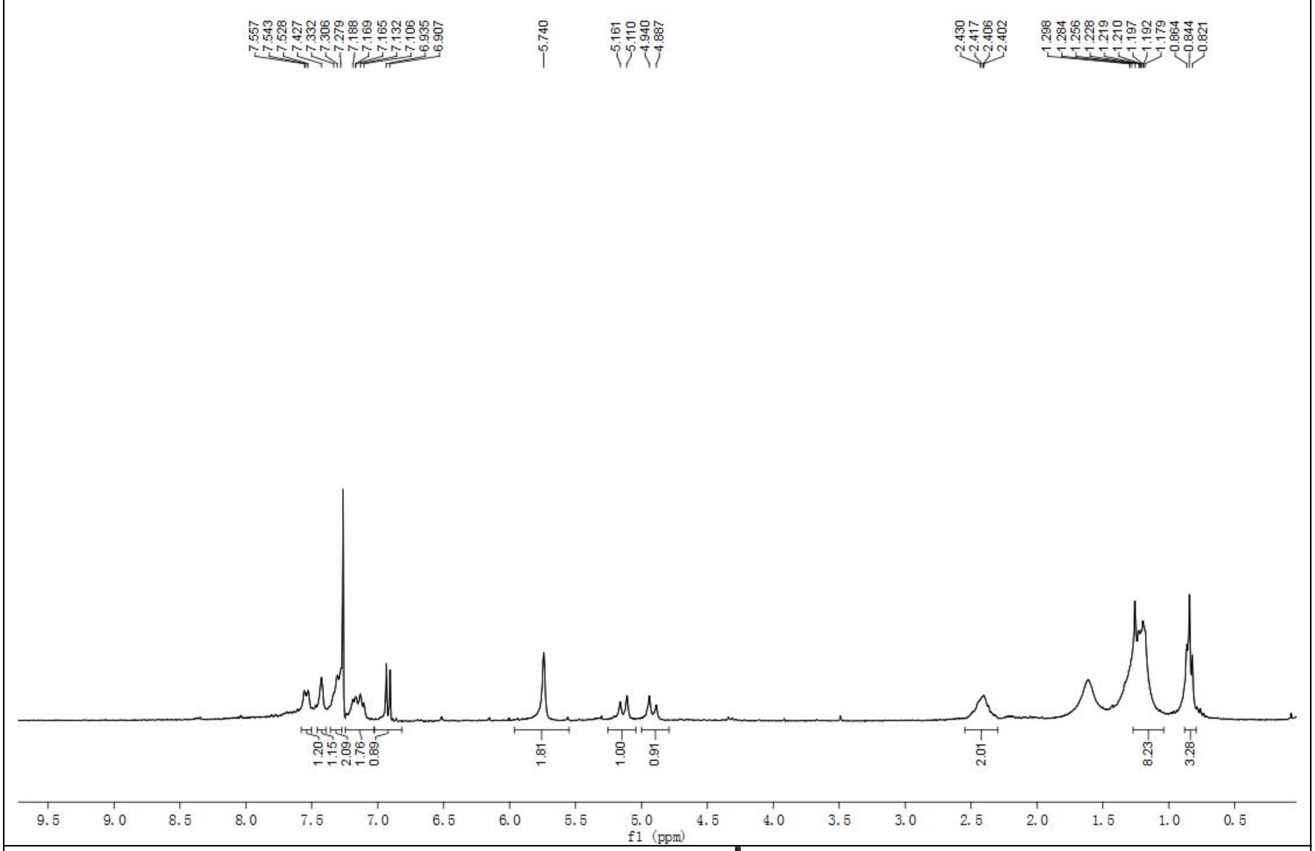

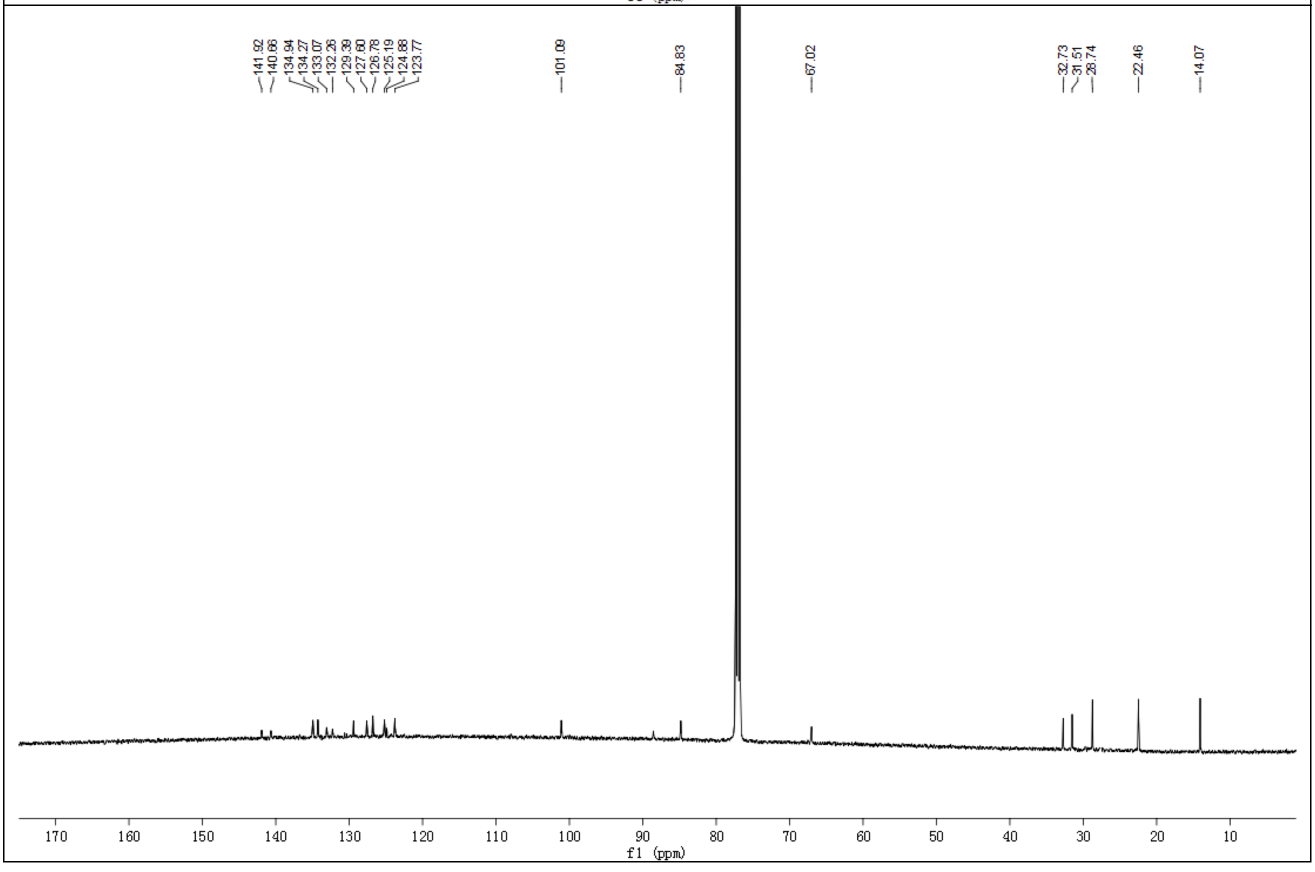

6-Phenyl-11b,13-dihydropyrido[2',3':4,5][1,3]oxazino[2,3-*a*]isoquinoline (3Aj)

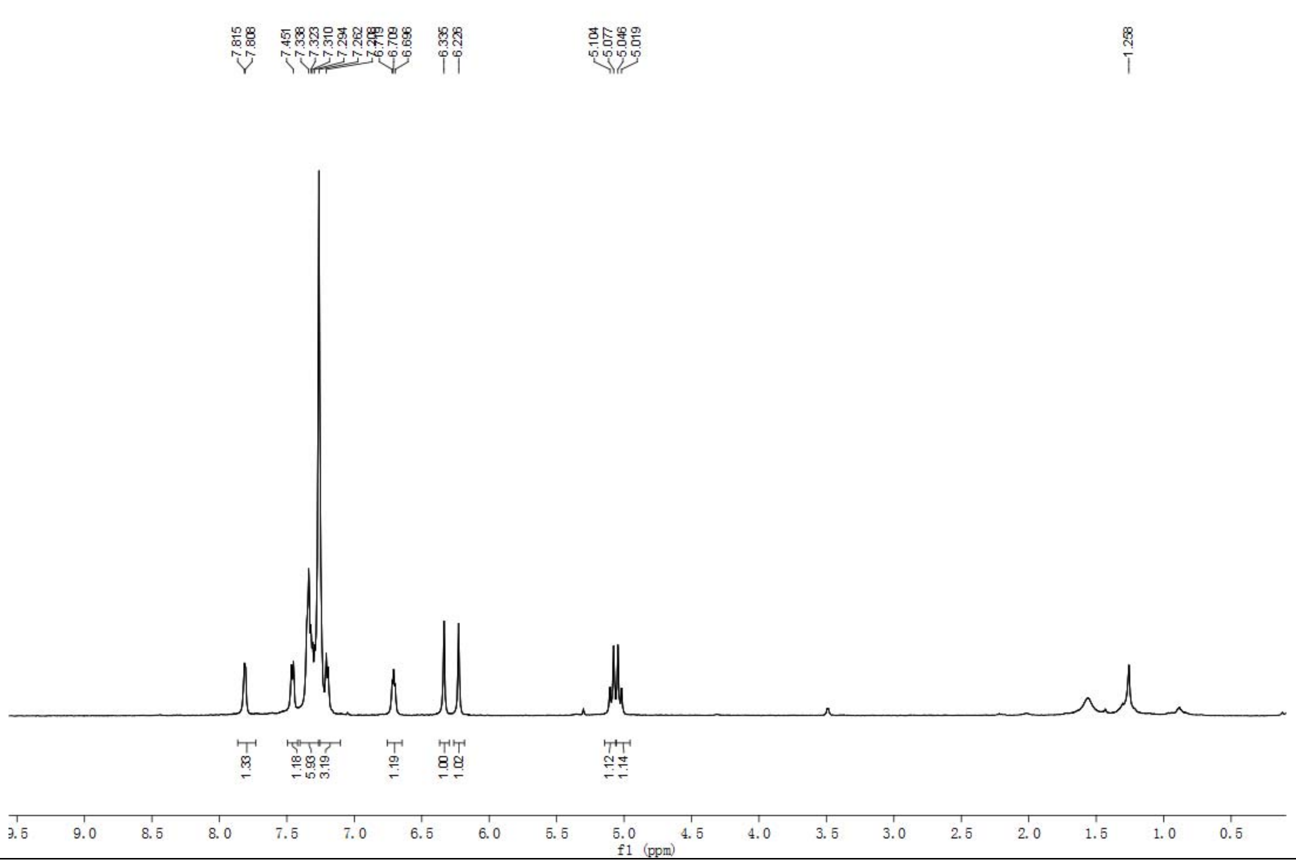

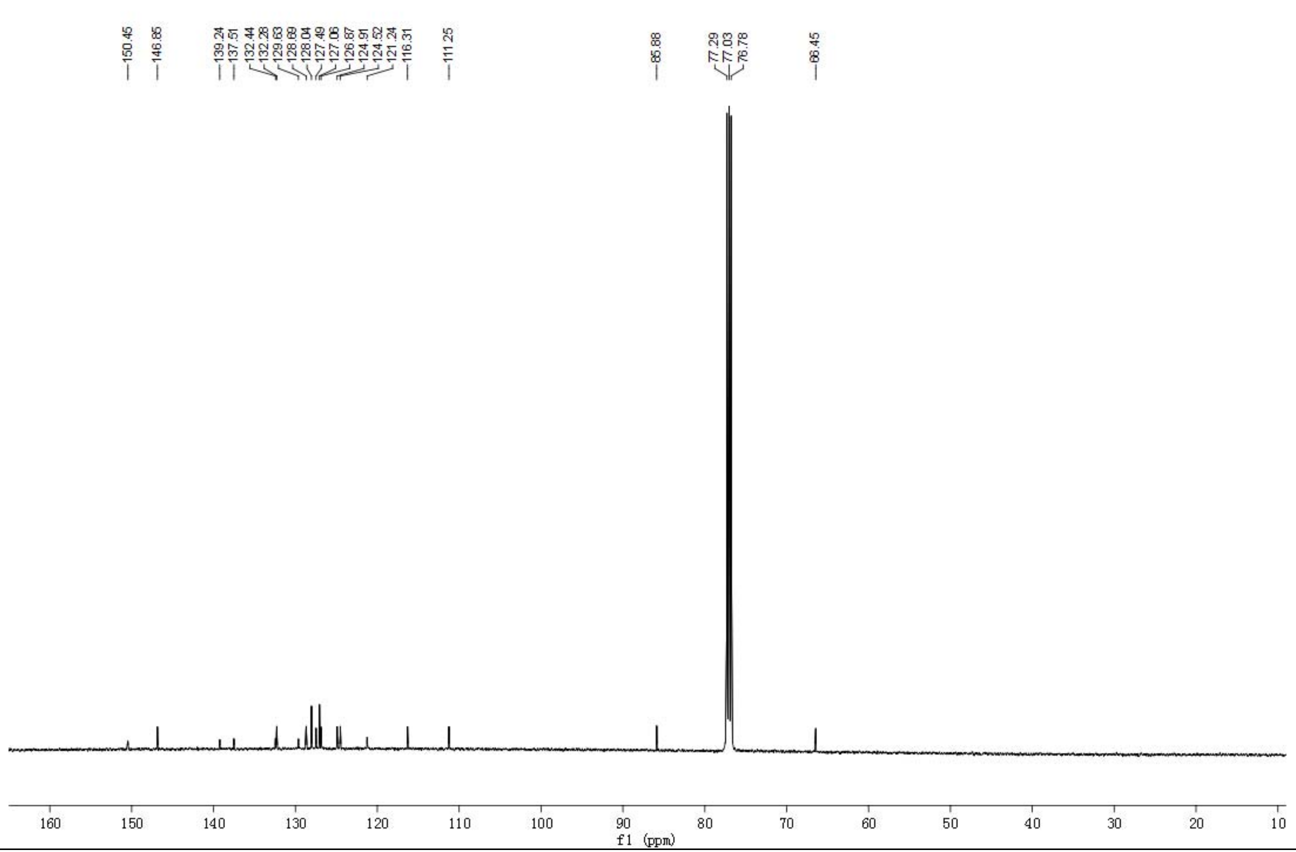

6-(4-Fluorophenyl)-11b,13-dihydropyrido[2',3':4,5][1,3]oxazino[2,3-*a*]isoquinoline (3Bj)

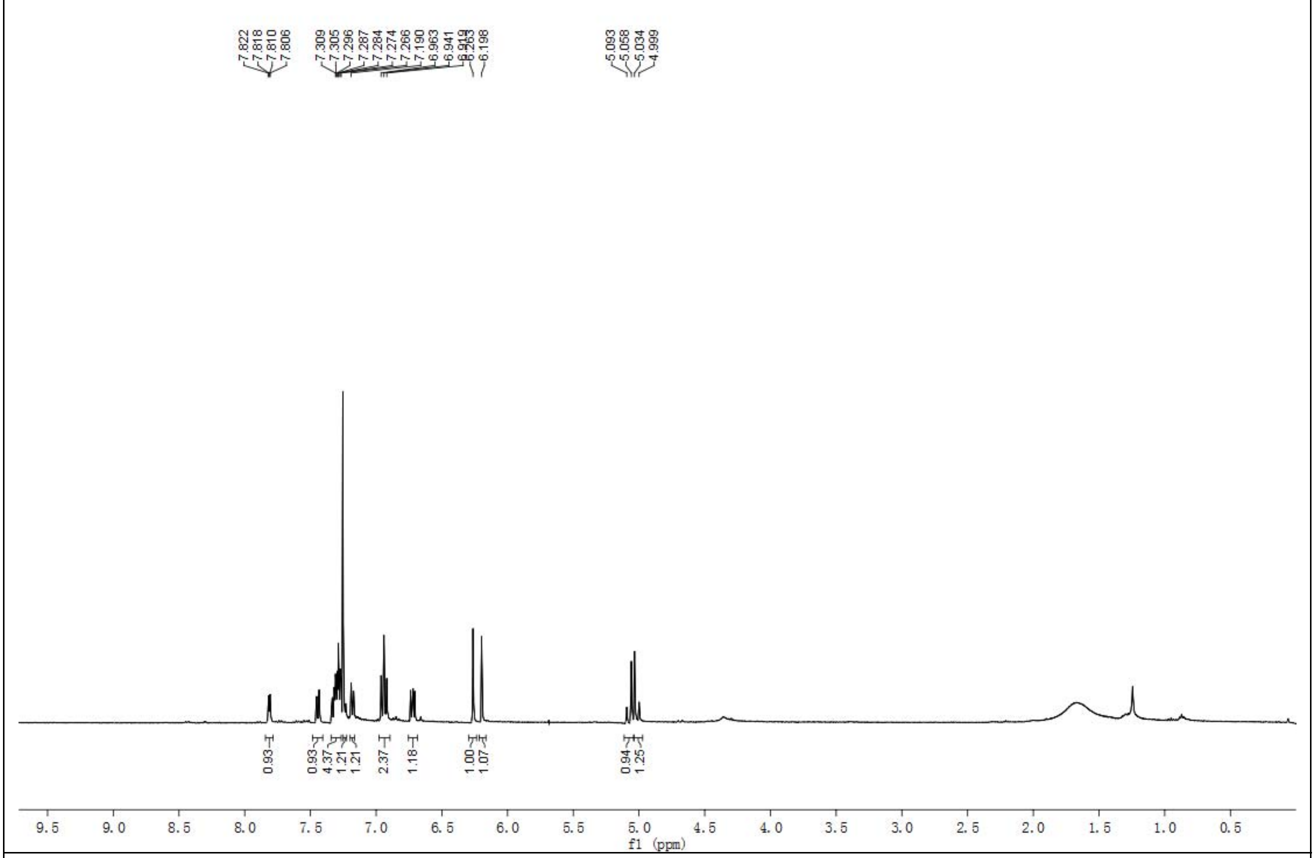

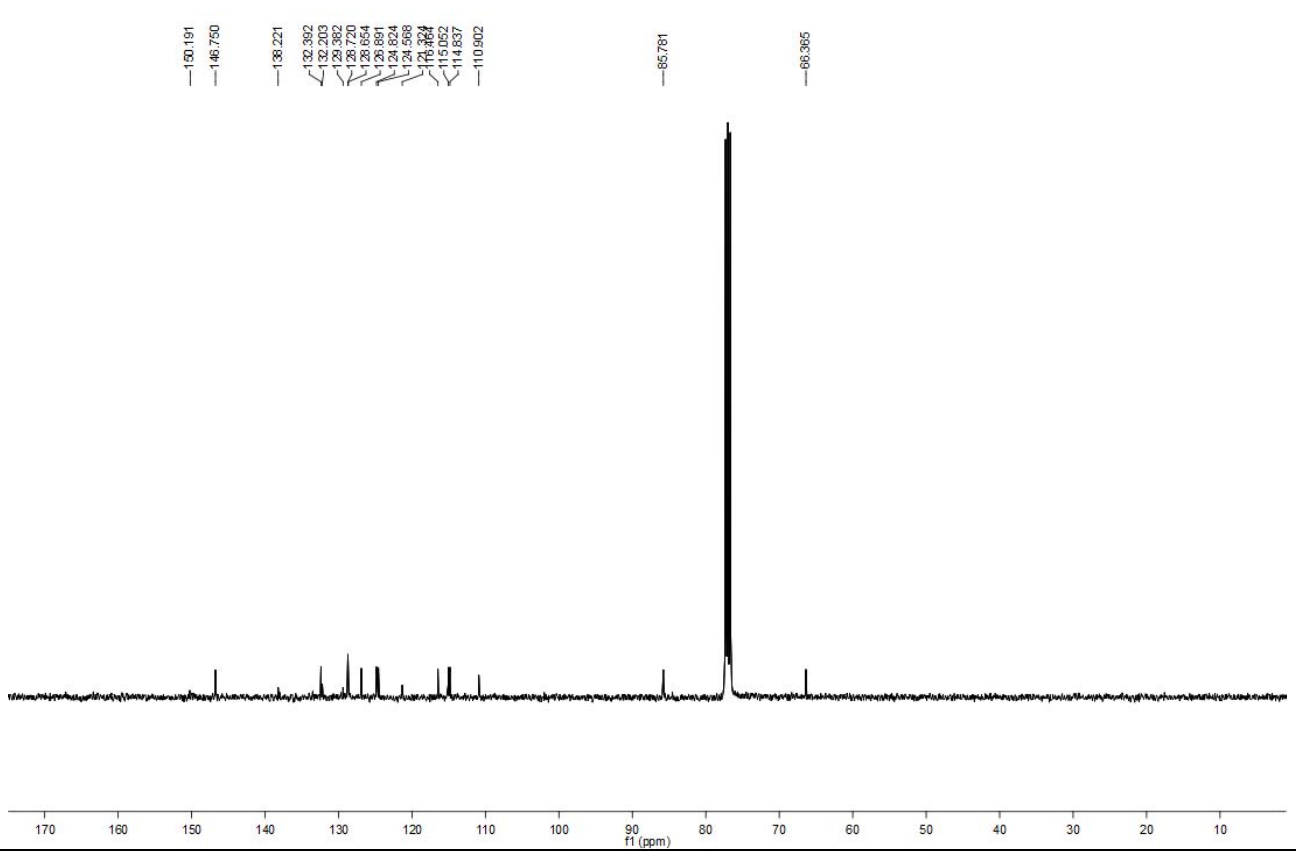

Supplement: Supplementary file 1 [file molecules-18-00814-s001.pdf]
